# Genome-wide identification and characterization of LRR-RLKs reveal functional conservation of the SIF subfamily in cotton (*Gossypium hirsutum*)

**DOI:** 10.1186/s12870-018-1395-1

**Published:** 2018-09-06

**Authors:** Ning Yuan, Krishan Mohan Rai, Vimal Kumar Balasubramanian, Santosh Kumar Upadhyay, Hong Luo, Venugopal Mendu

**Affiliations:** 1 Fiber and Biopolymer Research Institute (FBRI), Department of Plant and Soil Science, Texas Tech University, Lubbock, TX 79409 USA; 2 Department of Botany, Panjab University, Chandigarh, 160014 India; 3 Department of Genetics and Biochemistry, Clemson University, Clemson, SC 29634 USA

**Keywords:** *Gossypium hirsutum*, LRR-RLKs, Genome-wide analysis, Salt tolerance

## Abstract

**Background:**

As one of the largest subfamilies of the receptor-like protein kinases (RLKs) in plants, Leucine Rich Repeats-RLKs (LRR-RLKs) are involved in many critical biological processes including growth, development and stress responses in addition to various physiological roles. *Arabidopsis* contains 234 LRR-RLKs, and four members of Stress Induced Factor (SIF) subfamily (AtSIF1-AtSIF4) which are involved in abiotic and biotic stress responses. Herein, we aimed at identification and functional characterization of SIF subfamily in cultivated tetraploid cotton *Gossypium hirsutum*.

**Results:**

Genome-wide analysis of cotton LRR-RLK gene family identified 543 members and phylogenetic analysis led to the identification of 6 cotton LRR-RLKs with high homology to *Arabidopsis* SIFs. Of the six SIF homologs, GhSIF1 is highly conserved exhibiting 46–47% of homology with AtSIF subfamily in amino acid sequence. The *GhSIF1* was transiently silenced using Virus-Induced Gene Silencing system specifically targeting the 3’ Untranslated Region. The transiently silenced cotton seedlings showed enhanced salt tolerance compared to the control plants. Further, the transiently silenced plants showed better growth, lower electrolyte leakage, and higher chlorophyll and biomass contents.

**Conclusions:**

Overall, 543 LRR-RLK genes were identified using genome-wide analysis in cultivated tetraploid cotton *G. hirsutum*. The present investigation also demonstrated the conserved salt tolerance function of SIF family member in cotton. The *GhSIF1* gene can be knocked out using genome editing technologies to improve salt tolerance in cotton.

**Electronic supplementary material:**

The online version of this article (10.1186/s12870-018-1395-1) contains supplementary material, which is available to authorized users.

## Background

In order to sense outside environment and efficiently communicate between cells, both animals and plants use plasma membrane and/or cell wall localized receptors, which perceive and transduce signals to modulate gene expression. Toll-like receptors represent the most important kinase receptors involved in signal transduction process [1]. Plant receptor-like protein kinases (RLKs), on the other hand, is the most important membrane protein family involved in growth and development, stress response and various other biological processes [2]. Based on the structure of an extracellular domain, plant receptor-like protein kinases have been classified into various subfamilies such as S-RLK (S-domain RLK), LRR-RLK (Leucine-Rich Repeat RLK), CR4-class (CRINKLY4 RLK), WAK (Wall Associated Kinase), PR5-RLK (PR5-Like RLK), and Lectin class [3–9]. Among them, LRR-RLK is one of the largest subfamilies of the receptor-like protein kinases in plants with 234 members in *Arabidopsis* [2, 10–12] (Table 1). LRR domain specifically identifies and interacts with a wide variety of extracellular signaling ligands, conferring LRR-RLK’s ability to perceive apoplastic signals [13]. Studies on the FLS2 (Flagellin Sensitive 2)-BAK1 (Brassinosteroid Insensitive 1-associated receptor kinase 1) complex showed that the interaction between ligand and LRR domain induces a conformational change of kinase domain in the cytoplasm, which allows the kinase domain to transfer phosphates to downstream proteins, promoting the signal transduction from apoplast to symplast [13]. LRR-RLKs regulate various biological processes in plants, including steroid perception, cell proliferation, photomorphogenesis, biotic and abiotic stress responses [14–19]. For instance, SERKs (Somatic Embryogenesis Receptor Kinase) are essential receptors mediating brassinosteroid signal perception in *Arabidopsis* [20, 21]. Furthermore, SERK3/BAK1 and SERK4/BKK1 (BAK1-Like 1) are involved in defense signal transduction triggered by FLS2 or EFR [22]. In *Medicago spp.*, the *LRR-RLK* gene, *SRLK* has been shown to regulate the root response to salt stress [18]. Similarly, rice *Xa21D* gene encodes a membrane-anchored protein responsible for the pathogen recognition in disease resistance signaling pathway [23].

**Table 1:** Gene distribution comparison of *Arabidopsis* and cotton LRR-RLK subclades

Subclade	*G. hirsutum*	Ratio in the total LRR-RLKs	Subclade	*A. thaliana*	Ratio in the total LRR-RLKs
I	13	2.4%	I	44	18.8%
II	28	5.2%	II	15	6.4%
III	89	16.4%	III	45	19.2%
IV (1 &2)	12	2.2%	IV	4	1.7%
V	18	3.3%	V	10	4.3%
VI (1 &2)	30	5.5%	VI (1 & 2)	14	6.0%
VII	17	3.1%	VII	7	3.0%
VIII (1 &2)	40	7.4%	VIII	21	9.0%
IX	18	3.3%	IX	5	2.1%
X (1, 2, 3 & 4)	43	7.9%	X (a & b)	18	7.7%
XI (1, 2, 3 & 4)	93	17.1%	XI	30	12.8%
XII (1 & 2)	128	23.6%	XII	10	4.3%
XIII	13	2.4%	XIII (a & b)	10	4.3%
Others	1	0.2%	XVI	1	0.4%
Total	543			234	

Due to the significant importance of the LRR-RLK family members, genome-wide analysis has been performed in *Arabidopsis*, soybean, wheat, citrus, vernicia, maize, rice and poplar, facilitating identification and functional characterization of LRR-RLK genes in these species [12, 24–30]. LRR-RLKs in *Arabidopsis* are grouped into 14 subclades (LRR-I to LRR-XIV, which are distributed among all five chromosomes [12]. A total of 309, 467 and 531 LRR-RLKs have been identified in rice, soybean and allohexaploid wheat, respectively [24, 28, 30]. Despite the large numbers, the LRR-RLKs are highly conserved within the clades. However, differences in extracellular domains and the associated structure resulted in the functional specialization of individual members within the clades. For instance, *Arabidopsis* LRR-RLKs from subclade I harbor a malectin-like domain responsible for *N*-glycosylation and ER localization, which is not detected in other subclades [31]. Hence, phylogenetic analysis and functional characterization of each gene are important to understand their specific role in various organisms. We have recently identified and characterized a sub-family of LRR-RLK genes involved in biotic and abiotic stress signaling pathway in *Arabidopsis* [32]. The Stress Induced Factor (SIF) sub-family contains four members (SIF1–4), which respond to abiotic and biotic stresses. Further characterization of SIF2 protein demonstrated its role in stress signal transduction pathway in *Arabidopsis*.

*Gossypium hirsutum* is one of the widely cultivated crops in the world, which accounts for more than 95% annual global cotton production [33]. Globally, cotton is cultivated under diverse environmental conditions and exposed to various biotic and abiotic stresses. Individual cotton *LRR-RLK*s genes, such as *GhLRR-RL*, *GhBRI1*, *GhRLK1*, and *GbRLK*, have been characterized and demonstrated to play important roles in cotton development and stress resistance [34–37]. However, there is no comprehensive analysis of the LRR-RLK gene family in cotton. In the present study, we performed genome-wide analysis of LRR-RLK gene family in *G. hirsutum* using the recently released cotton full genome sequence (https://www.cottongen.org/data/download/genome). A total of 543 GhLRR-RLK proteins were identified, and 542 of them were grouped into 13 clades in a phylogenic tree. Chromosomal distribution, gene duplication, gene and protein structure analysis, functional annotation, and expression profiling of these genes further led to the identification of *Arabidopsis* SIF subfamily of homologs in cotton. Transient silencing of *GhSIF1* using virus-induced gene silencing (VIGS) system conferred salt tolerance in cultivated tetraploid cotton. Overall, the present study demonstrates the functional conservation of SIF sub-family in cotton, suggesting its potential use for crop improvement through molecular breeding, biotechnology or genome editing approaches.

## Results

### Identification of *LRR-RLK* gene family in *Gossypium hirsutum* TM-1

We have downloaded publicly available *G. hirsutum* TM-1 accession reference genome data and performed genome-wide similarity search to identify the LRR-RLK gene family using the sequences of *Arabidopsis* LRR-RLK proteins as query [12]. A stringent filtration of the Blast identified sequences for the presence of a minimum of one LRR repeat, a kinase domain and a transmembrane region resulted in identification of a total of 543 *G. hirsutum LRR-RLK* family members (Additional file 1: Table S1). Full-length genomic, coding and amino acid sequences for all the validated *G. hirsutum* LRR-RLK family members were fetched from the reference genome sequence with their original gene ID and used for further characterization.

### Phylogenetic analysis of cotton LRR-RLKs

**Fig. 1:**
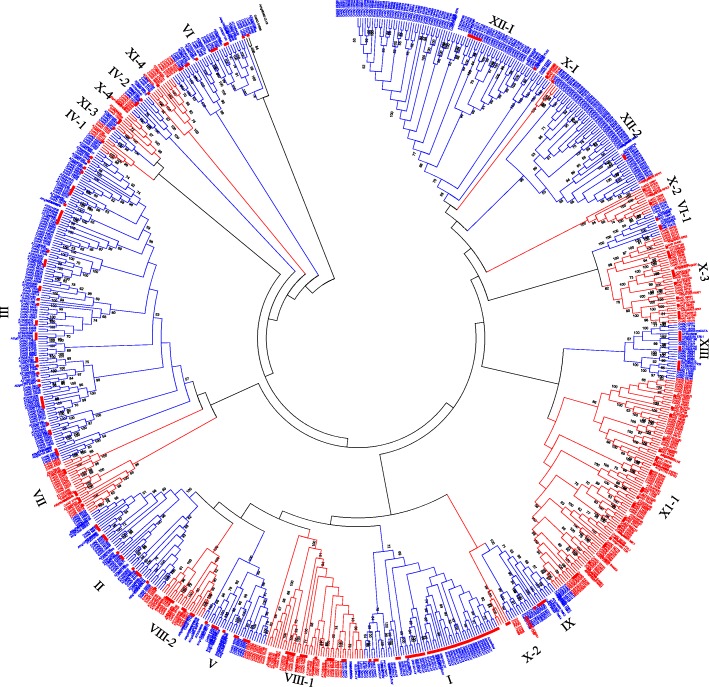
Phylogenetic analysis of *Gossypium hirsutum* LRR-RLK protein sequences. The evolutionary history was inferred using the Neighbor-Joining method with 1000 bootstrap replication. The evolutionary distances were computed using the p-distance method and are in the units of the number of amino acid substitutions per site. The analysis involved 543 *G. hirsutum* LRR-RLK protein sequences and 234 *Arabidopsis thaliana* LRR-RLK protein sequences. All positions containing gaps and missing data were eliminated. Evolutionary analyses were conducted in MEGA6

Protein sequence alignment and phylogenetic analysis were performed using 543 GhLRR-RLK and 234 *Arabidopsis* LRR-RLK protein sequences to study the evolutionary relationships [11, 12]. *G. hirsutum* protein sequences that were grouped with AtLRR-RLK were defined as members of the corresponding *Arabidopsis* subclade. Using the *Arabidopsis* LRR-RLKs as references, 542 GhLRR-RLKs were grouped into 13 subclades in the Neighbor-Joining phylogenetic tree, while remaining one protein, CotAD_01838, was clustered together with an *Arabidopsis* LRR receptor-like protein At1G65380 (CLV2), which was not assigned to any *Arabidopsis* subclade (Fig. 1 & Additional file 1: Table S1). The size of each GhLRR-RLK subclade varied significantly. For instance, the largest subclade XII contains 128 members, while the smallest subclade IV contains only 12 members. Broadly, the relative size of each GhLRR-RLK subclade was almost similar to *Arabidopsis*, except subclade I and subclade XII (Table 1) [38]. In *Arabidopsis*, subclade I has 44 members representing 18.8% of the total AtLRR-RLKs, but *G. hirsutum* subclade I, which contains 13 members comprises only 2.4% of the total GhLRR-RLKs. The subclade XII, 10 LRR-RLK sequences represent only 4.3% of the total *At*LRR-RLKs, while GhLRR-RLK-XII subclade is composed of 128 members representing 23.6% of the total GhLRR-RLKs.

**Fig. 2:**
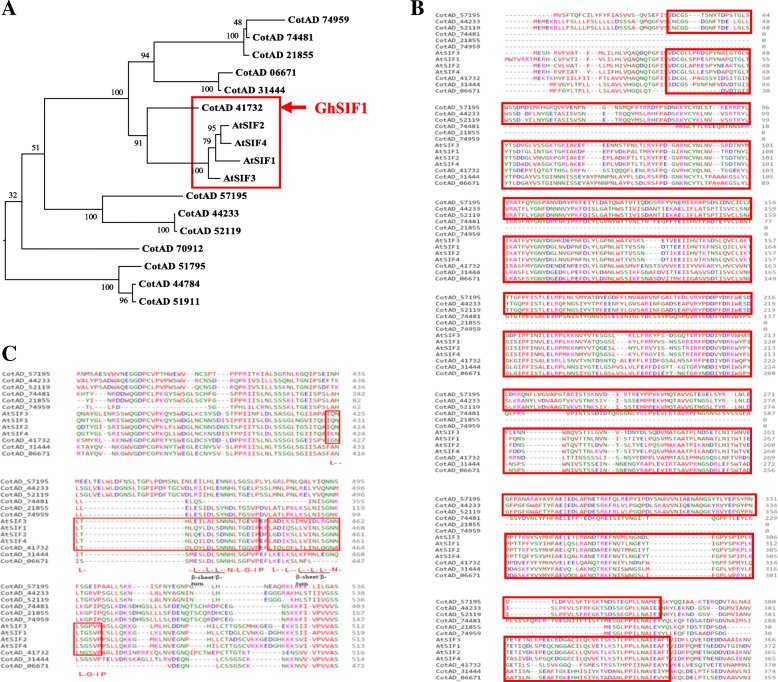
Phylogenetic tree of *Arabidopsis thaliana* SIF family and *G. hirsutum* LRR-RLK subclade I protein kinases. **a** The phylogenetic tree is constructed using the Maximum Likelihood method based on the JTT matrix-based model with MEGA 6. The analysis involved 13 *G. hirsutum* LRR-RLK subclade I protein sequences with 4 of *Arabidopsis thaliana* SIF family protein sequences. All positions containing gaps and missing data were eliminated. **b** Alignment of Malectin-like domain and (**c**) LRR domain of AtSIFs and GhLRR-RLKs protein sequences. Protein alignment analysis was conducted with Clustal Omega (https://www.ebi.ac.uk/Tools/msa/clustalo/). In the alignment, amino acid residues are depicted with different colors for distinguishing. Ellipses represent amino acid gaps. The numbers indicate the positions of amino acid residues. Malectin-like domain in (**b**) and LRR domains in (**c**) are highlighted with red boxes. In (**c**), ‘L--L--L--L-L--N-L--G-IP-’ indicates the conserved amino acid sequence of LRR domain, and the predicted β-strand/β-turn structure is underlined as --L-L--, where the ‘-’ stands for non-conserved amino acid residues, the ‘L’ represents Leu or Ile, and the ‘I’ represents Val or Ile

To investigate whether *G. hirsutum* contains homologs of *Arabidopsis* SIF subfamily genes (*AtSIF1*-*AtSIF4*) [32], we generated a Maximum Likelihood phylogenetic tree using AtSIF1-AtSIF4 proteins with *G. hirsutum* subclade I LRR-RLKs proteins which showed high homology with *AtSIF2* (*At1G51850*) (Fig. 2). The phylogenetic tree showed that 9 GhLRR-RLKs have very close evolutionary relationship with the four *Arabidopsis* LRR-RLKs (Fig. 2a). Among these 9 GhLRR-RLKs, one cotton LRR-RLK (CotAD_41732) showed very high homology with AtSIF subfamily (Fig. 2a). To further understand the protein conservation between AtSIFs and the nine cotton LRR-RLKs, multiple sequence analysis was performed (Fig. 2 & Additional file 2: Data S1). The result showed that only six proteins out of the 9 GhLRR-RLKs contain the Malectin-like domain, which is also present in AtSIFs (Fig. 2b). LRR domain is one of the most critical domains in LRR-RLKs as it offers LRR-RLKs the ability of ligand recognition and interaction [39]. Highly conserved LRR domains in LRR-RLKs usually indicate functional conservation [39]. The amino acid sequence comparison of the LRR domains in these six LRR-RLKs which contain Malectin-like domain showed that CotAD_41732 exhibited the highest similarity with the AtSIFs, as it contains two highly conserved LRR motifs in the same region of the extracellular domains (Fig. 2c). Other cotton LRR-RLKs contain either different number of LRR motifs (such as CotAD_57195, CotAD_44233, CotAD_52119, CotAD_31444) or gaps in the critical LRR domains (such as CotAD_74481, CotAD_06671), or the size is significantly shorter than that of the AtSIFs (such as CotAD_21855 and CotAD_74959) (Fig. 2c). We, therefore, refer CotAD_41732 which showed highest similarity as GhSIF1 hereafter.

### Chromosomal distribution of *GhLRR-RLKs*

**Fig. 3:**
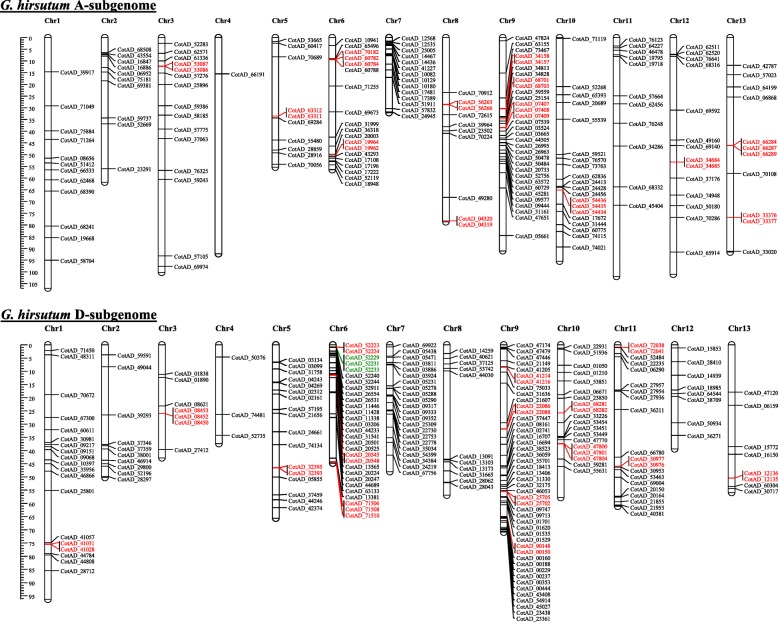
Chromosomal localization and distribution of *G. hirsutum LRR-RLKs.* Chromosomal coordinates of *GhLRR-RLK*s were plotted on the *G. hirsutum* A-subgenome and D-subgenome specific chromosomes. Genes in red color and green indicate the tandem duplication. Genes located on unanchored scaffolds are not included in this figure. All the chromosomes are drawn using the scale (in Mb) shown in the figure

To further investigate the evolutionary history of *GhSIF1* as well as other *GhLRR-RLKs*, we analyzed their chromosomal distribution on both A and D subgenomes of *G. hirsutum* (Fig. 3 & Additional file 1: Table S2). The *GhLRR-RLK* genes were distributed on all chromosomes of both subgenomes but at a different frequency (Fig. 3). Out of 543 genes, 179 and 219 genes could be confirmed at A and D subgenomes, respectively; whereas 145 genes were located on scaffolds (Additional file 3: Figure S1). A maximum of 32 and 46 genes and a minimum of one and three genes were located on chromosome 9 and chromosome 4 of A and D-subgenomes, respectively (Fig. 3 & Additional file 3: Figure S1). *GhSIF1* was located on the scaffold 1841.1 (Additional file 3: Figure S1 and Additional file 1: Table S2).

A total of 42 tandem duplication events (TDEs) were identified involving 110 genes distributed in subclades II, III, VIII_1, X_4, XI_1, XII_1 and XII_2 (Fig. 3). Subclade XII_1 showed a maximum of 14 events involving 40 genes followed by subclade XII_2 with 12 events involving 32 genes. Out of 42 TDEs, 13 were observed on 8 chromosomes (Chr. 3, 5, 6, 8, 9, 10, 12 and 13) of A-subgenome (Fig. 3), while 15 were found on 8 chromosomes (Chr. 1, 3, 5, 6, 9, 10, 11 and 13) of D-subgenome. The remaining 14 TDEs were observed on 10 unassigned scaffolds (Scaffold 2911.1 with three duplication events and scaffold 235.1 and 3068.1 with two events each). Overall, the analysis showed a high proportion of tandem duplications involving ~ 1/5th of the LRR-RLKs.

### Analysis of gene structure (exon-intron organization) of *GhLRR-RLKs*

Exon-intron structures of 543 *GhLRR-RLK* genes, including the *GhSIF1*, were analyzed and organized in different groups according to their subclades. As shown in Additional file 3: Figure S2 (A-I), the exon-intron organization of *LRR-RLK* genes showed high variation among subclades, whereas, within subclade the genes displayed comparable structure in terms of number, size and position of exons. The conservation of gene structure within clades indicates that the *LRR-RLK* genes within clades indeed have very close evolutionary relationships in the phylogenetic tree. Based on exon-intron structures, the *GhLRR-RLKs* could be classified into three groups (Additional file 3: Figure S2 A-I). All the members of subclade I, II, V, VI-2, VIII (1 & 2), and most members of subclade XIII comprised multiple but relatively short exons, while the members of subclade III, IV (1 & 2), VI-1, VII, IX, X (1–4), XI (1–4), two members of subclade XIII and *CotAD_01838* consisted of several long exons. Subclade XII (1 & 2) genes showed a unique pattern with the combination of long exons and short exons.

### Protein structure analysis

GhLRR-RLKs showed a wide variation in their length ranging from 234 to 1878 amino acid residues (aa) (Additional file 3: Figure S3 & Additional file 1: Table S1) with an average length of ~ 855.8 aa and an average molecular weight of 94.2 kDa. The CotAD_60784 protein in subclade XII was the smallest GhLRR-RLK with a length of 234 aa, while the longest protein was CotAD_44505 with a length of 1878 aa. The isoelectric point (pI) range of GhLRR-RLKs was 4.88–9.62 (Table 2 and Additional file 1: Table S1). The protein of specific interest, GhSIF1 comprised of 874 aa with a molecular weight of 98.2 kDa and pI 5.07.

**Table 2:** Molecular properties of cotton LRR-RLK gene family subclades

				Range (Group-wise)		
Subclades	No. of Genes	Gene size (Kb)	Protein size (aa)	pI	Mol. Wt. (Kda)	No. of LRRs
I	13	1.674–8.986	268–932	5.07–7.01	29.1–104.3	1
II	28	2.911–7.585	491–650	5.24–8.51	54.7–71.7	1–2
III	89	1.793–7.562	343–1067	5.56–9.46	37.4–115.7	1–6
IV_1	5	2.696–3.355	865–974	5.5–7.02	94.6–107.5	3–8
IV_2	7	2.156–2.580	640–684	6.44–8.61	69.9–75.6	1
V	18	3.730–7.210	690–835	5.56–8.79	76.1–91.1	1–3
VI_1	7	2.204–2.558	680–852	6.3–9.47	75.6–93.8	1–6
VI_2	23	2.642–6.460	573–790	4.88–9.11	63.6–88.0	1–3
VII_1	10	2.745–3.425	885–1141	5.44–6.94	96.0–124.8	1–8
VIII_1	31	3.565–13.805	661–1097	5.04–8.00	72.9–121.1	1–4
VIII_2	9	4.463–12.060	819–1343	5.49–8.68	88.4–147.2	1–3
IX	18	2.840–3.845	893–979	5.38–8.83	98.1–107.0	1–3
X_1	3	3.123–3.585	958–1099	5.46–7.03	105.9–121.0	3–5
X_2	9	2.762–3.859	920–1140	6.03–8.43	100.5–124.7	1–7
X_3	24	1.577–3.842	525–1280	5.7–7.85	58.1–139.9	1–6
X_4	7	2.745–2.939	885–944	5.71–8.91	97.3–102.8	2–4
XI_1	76	1.942–9.841	408–1878	5.16–9.13	45.5–207.6	1–5
XI_2	3	1.919–2007	639–643	8.82–9.08	71.1–71.3	2
XI_3	4	2.339–2.918	556–945	5.89–8.13	60.8–104.2	1–4
XI_4	10	3.049–4.399	808–974	6.73–8.89	88.9–99.9	2–4
XII_1	77	1.811–12.435	441–1539	5.53–9.02	48.1–170.0	1–7
XII_2	51	1.259–13.013	234–1209	4.96–9.62	26.0–133.5	1–7
XIII	14	3.211–6.506	400–1829	5.51–8.23	43.7–198.9	1–7
Others	1	3.003	948	5.54	104.9	5

To investigate the protein structure, each GhLRR-RLKs was subjected to Blast2GO server for InterProScan domain distribution analysis [40] (Additional file 3: Figure S4 & Additional file 1: Table S3). According to the result of InterProScan analysis, LRR and protein kinase-like domain (KD) were the two most conserved domains among the 543 GhLRR-RLK proteins, while KD was less conserved when compared to the LRR domain as it was absent in CotAD_01838 which was an outlier in the phylogenetic tree (Additional file 3: Figure S4). A Malectin-like domain was identified in 13 GhLRR-RLKs, including GhSIF1 (Additional file 3: Figure S4). Other protein domains, such as Cyclic nucleotide-binding domain (IPR000595), P-loop containing nucleoside triphosphate hydrolase (IPR027417), Kinesin motor domain (IPR001752), Glycoside hydrolase superfamily (IPR017853), Rho GDP-dissociation inhibitor domain (IPR024792), Galactose-binding domain-like (IPR008979), Gnk2-homologous domain (IPR002902), Ubiquitin domain (IPR000626), Ubiquitin-related domain (IPR029071), and Chlorophyll a/b binding protein domain (IPR023329) were also identified in some GhLRR-RLK sequences, indicating that GhLRR_RLKs may be involved in diverse functions such as protein binding, kinesin, glycoside hydrolase, ubiquitin-related, or light reception (Additional file 3: Figure S4 & Additional file 1: Table S3).

Motif analysis using Motif Alignment & Search Tool (http://meme-suite.org/tools/mast) with extracellular regions revealed the occurrence of 8 LRR submotifs (LRR_1, LRRNT_2, LRR_3, LRR_4, LRR_5, LRR_6, LRR_8, and LRR_9) in the LRR clan (CL0022), together with Malectin-like domain in the 13 subclades (Additional file 3: Figure S5 A-K) [41], but the distribution of these domains was highly divergent. LRR_1 and LRR_8 domains were the most abundant and were identified in 96.3% and 71.0% sequences, respectively. On the contrary, LRR_3 and LRR_5 were the rarest, which were identified in only 3.8% and 3.3% GhLRR-RLKs, respectively. Further, a significant number (69.2%) of subclade I members possess a Malectin-like domain in place of LRRNT_2 at the N-terminus. Interestingly, the N-terminal Malectin-like domain could only be found in subclade I, implying more special functions of the members in this clade than those of any other subclades. Although Malectin-like domain was also identified in four LRR-RLKs belonging to other Subclades (III, VIII-2, and XI-4), however they are located on the C-terminal not the N-terminal of the protein. A total of 391 GhLRR-RLKs consisted of various signal peptides at their N-terminal (Additional file 3: Figure S6 & Additional file 1: Table S4), however each GhLRR-RLK comprised a transmembrane domain (Additional file 1: Table S4). The protein structure analysis showed that GhSIF1 consisted of a 22-aa signal peptide, a Malectin-like domain, an LRR-8 motif, a transmembrane domain, and an intracellular kinase domain (Additional file 3: Figure S5 A and Additional file 1: Table S4).

### Functional annotation and gene ontology analysis

Cellular component analysis conducted with Blast2GO software showed that 542 *GhLRR-RLK*s were predicted to be located on the membrane system, and 538 proteins were predicted to be localized in cell part, followed by organelle (286), membrane part (209), symplast (206), and cell junction (206) (Additional file 3: Figure S7 and Additional file 1: Table S3) while some proteins were predicted to be extracellular (95). The biological processes analysis (Additional file 3: Figure S7 and Additional file 1: Table S3) showed that the *GhLRR-RLK*s are involved in ‘cellular process’ (504), ‘response to stimulus’ (502), ‘single-organism process’ (498), ‘biological regulation’ (476), ‘signaling’ (411), and ‘metabolic process’ (410). Some proteins obtained the GO terms ‘developmental process’ (335), ‘multicellular organismal process’ (321), ‘reproduction’ (261), which were followed by ‘multi-organism process’ (168), ‘cellular component organization or biogenesis’ (146), and ‘localization’ (109). Molecular function analysis showed most *GhLRR-RLK*s displayed ‘catalytic activity’ (517), ‘binding’ (513), ‘signal transducer activity’ (152) and ‘molecular transducer activity’ (127) functions (Additional file 3: Figure S7 and Additional file 1: Table S3). A detailed information on specific cellular component, biological processes, and molecular function was performed and presented in the additional information (Additional file 3: Figure S8-S10). Specifically, *GhSIF1* was predicted to be a negative regulation factor of an abscisic acid-activated signaling pathway, indicating it may play a negative role in the abiotic stress tolerance mechanism (Additional file 1: Table S3). Furthermore, the Blast2Go also indicated that *GhSIF1* could even respond to biotic stress (Additional file 1: Table S3).

### *GhLRR-RLK* gene expression analysis in various organs and across fiber developmental stages

**Fig. 4:**
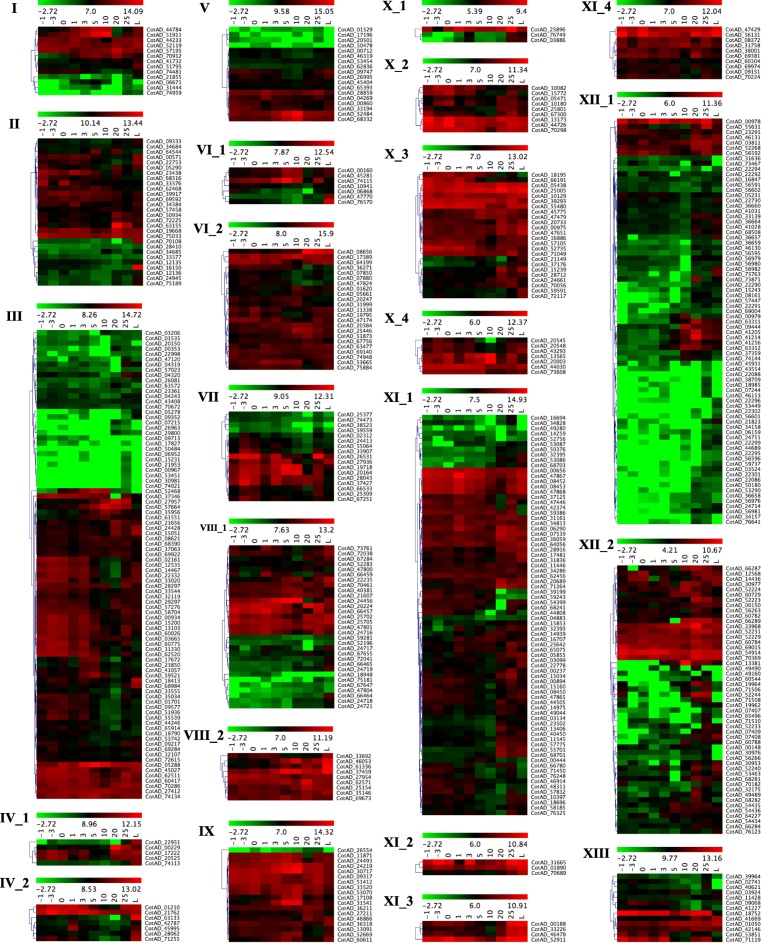
Expression analysis of *G. hirsutum* LRR-RLKs. Hierarchically clustered heatmap for individual subclades of *G. hirsutum LRR-RLK* genes in − 3 dpa ovule, − 1 dpa ovule, − 0 dpa ovule, 1 dpa ovule, 3 dpa ovule, 5 dpa fiber, 10 dpa fiber, 20 dpa fiber, 25 dpa fiber, and leaves. Scales used to prepare heatmap is included with individual subclade specific heatmaps

Publicly available cotton transcriptome datasets from *G. hirsutum* TM-1 were used to investigate the expression pattern of 543 LRR-RLK genes in leaves and across the different fiber developmental stages (− 3, − 1, 0, 1, 3, 5, 10, 20, and 25 dpa (day post anthesis)) (Fig. 4 and Additional file 1: Table S5 and S6). Subclade specific heatmaps were generated to show the expression pattern of LRR-RLK genes using the self-normalized log converted RPKM values obtained by mapping transcriptome datasets (Additional file 3: Figure S11). Most of the genes of subclades VI_2, VIII_2, IX, X_2, X_3, X_4, XI_2, XI_3 and XI_4 showed higher expression in all the stages of cotton fiber development indicating a potential role of these subclades genes in fiber development. However, members of I, II, III, IV_1, IV_2, V, VI_1, VII, VIII_1, X_1, XI_1, XII_2, and XIII subclades showed clusters of genes with low, moderate as well as high expression levels at various stages of fiber development. Most of the genes belonging to cluster XII_1 were low to moderately expressed except one small sub-cluster of highly expressed genes.

**Fig. 5:**
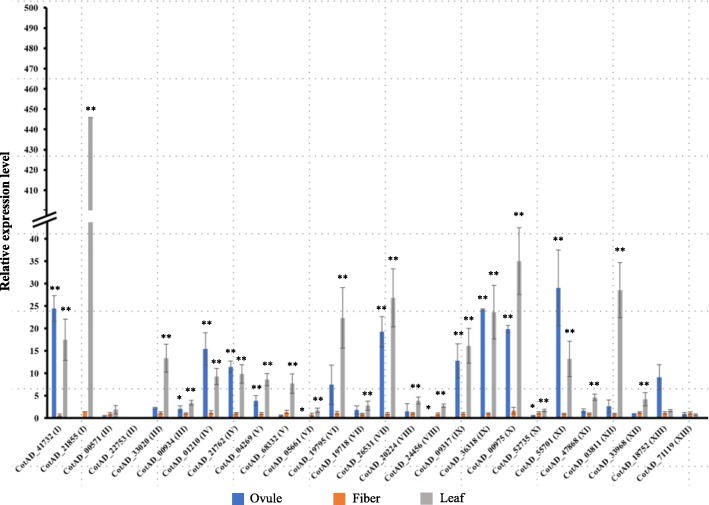
Real-time RT-PCR analysis of *G. hirsutum* LRR-RLKs expression. Ovule, fiber, and leaf tissue samples were collected at 5 dpa from cotton plants grown in the green house for real-time RT-PCR analysis. The expressions of 26 *G. hirsutum* LRR-RLKs in various subclades were analyzed. *GhActin2* was used as the internal reference gene. Data shown are an average of three technical replicates for three independent biological replicates. Error bars represent S.D. (*n* = 9). The statistically significant difference between fiber and other tissues was determined by t-test. *P* < 0.05 was marked as *. *P* < 0.01 was marked as **

To further confirm the expression of *LRR-RLK* genes, quantitative PCR analysis was performed with 26 *GhLRR-RLK* genes (two representative genes from each subclade) including *GhSIF1* (*CotAD_41732*) in leaf, 5 dpa ovule and 5 dpa fibers. As shown in Fig. 5, most of the *GhLRR-RLK* genes exhibited similar expression patterns as they had a significantly higher expression in ovule and leaf tissues than that in fiber tissue, except *CotAD_00571*, *CotAD_52735* and *CotAD_71119,* which were expressed at similar levels in all three tissues. Specifically, *CotAD_22753* could not be detected in any tissues, consistent with the transcriptome results.

### Gene expression and transient silencing of *AtSIF* homolog in cotton

**Fig. 6:**
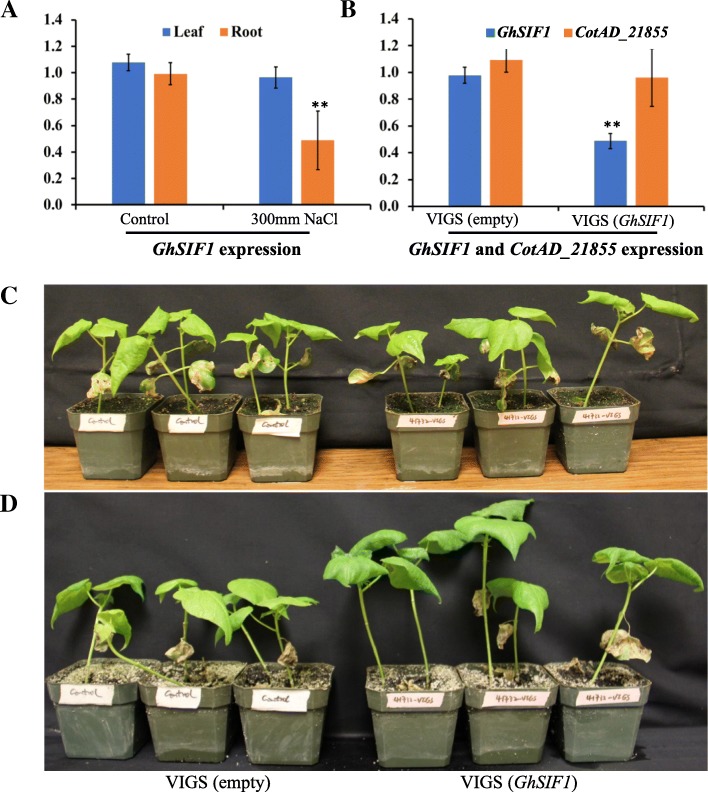
Expression and phenotypic analyses of *GhSIF1* under salt treatment and in VIGS treat cotton plants. (**a**) Cotton (*G. hirsutum*) seeds germinated on ½ MS medium were transferred to ½ MS with or without 300 mM NaCl medium. Ten days later, leaves and roots were collected for real-time PCR analysis. *GhActin2* was used as the reference gene. (**b**) 10 days old cotton plants (*G. hirsutum*) with two cotyledon leaves were infiltrated with TRV1 and empty TRV2 (as control) or TRV2-*GhSIF1* (targeting *GhSIF1* mRNA). Ten days later, leaf samples were collected for real-time PCR analysis. *GhActin2* was used as the reference gene. Data shown are an average of three technical replicates for two independent biological replicates. Error bars represent S.D. (*n* = 6). The statistically significant difference was determined by t-test. *P* < 0.05 was marked as *. *P* < 0.01 was marked as **. Pictures were taken (**c**) before salt treatment and (**d**) 18 days after salt treatment

The real-time PCR result showed that *GhSIF1* was significantly down-regulated in the salt-treated root tissue (Fig. 6a), similar to *Arabidopsis SIF1* and *SIF2* indicating a potential role of *GhSIF1* in the salt tolerance in cotton [32]. To further study the function of *GhSIF1*, we transiently silenced *GhSIF1* expression in cotton plants using Tobacco Rattle Virus (TRV) mediated virus-induced gene silencing system [42]. A 371 bp *GhSIF1* cDNA fragment was inserted in the TRV-2 to transiently silence *GhSIF1* mRNA using agroinfiltration. The region was selected from the specific 3’UTR (Untranslated Region) as the coding region showed high homology among *LRR-RLKs*. Ten days old cotton plants with two cotyledon leaves were infiltrated with pTRV1 and with pTRV2 (*GhSIF1*) along with pTRV1 and pTRV2 (empty) as a control. Leaf samples of control as well as *GhSIF1* targeting plants were collected 10 days after infiltration for gene expression analysis. The expression of *GhSIF1* was significantly down-regulated in VIGS (*GhSIF1*) infiltrated plants compared to the control plants (Fig. 6b). To insure the specificity of VIGS mediated suppression of *GhSIF1*, the expression of another gene *CotAD_21855,* which has 66% similarity with *GhSIF1* CDS (Coding Sequence) was analyzed. Gene expression analysis showed that the expression of *CotAD_21855* was not affected in the pTRV2(*GhSIF1*) silenced plant indicating the specificity of the VIGS system towards *GhSIF1* (Fig. 6b).

### Evaluation of salt tolerance of the *GhSIF1* silenced plants

**Fig. 7:**
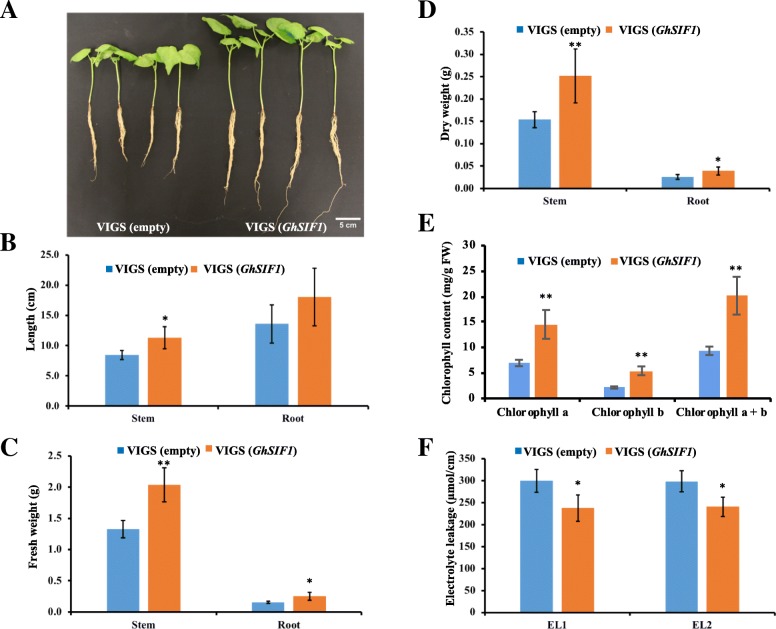
Down-regulation of *GhSIF1* leads to enhanced salt tolerance in VIGS treated cotton plants. Ten days old cotton plants (*G. hirsutum*) with two cotyledon leaves were infiltrated with TRV1 and empty TRV2 (as control) or TRV2-*GhSIF1* (targeting *GhSIF1* mRNA). Ten days later, plants were treated with 300 mM NaCl for 2 weeks. **a** Pictures were taken 18 days after salt treatment. **b** Shoot length and Root length, **c** fresh weight, **d** dry weight, **e** chlorophyll content, and **f** electrolyte leakage of control plants and *GhSIF1* targeting plants were measured. For (B-D) data shown are an average of eight independent biological replicates. Error bars represent S.D. (*n* = 8). *P* < 0.05 was marked as *. *P* < 0.01 was marked as **. For (E-F) data shown are an average of three technical replicates for five independent biological replicates. Error bars represent S.D. (*n* = 15). *P* < 0.05 was marked as *. *P* < 0.01 was marked as **. VIGS(empty): control plant. VIGS(*GhSIF1*): *GhSIF1* targeting plant

Gene silenced plants were evaluated for the salt tolerance in the presence of 300 mM NaCl for 2 weeks. Cotton plants with *GhSIF1* silencing exhibited better performance compared to control plants (Fig. 6c & d). The results showed that *GhSIF1* silenced plants displayed significantly longer shoot and more biomass than the control plants (Fig. 7). Previous studies showed that salt stress induce the reactive oxygen species, which results in chlorophyll degradation and membrane permeability leading to the reduction in chlorophyll content and high electrolyte leakage [43, 44]. The results showed that the chlorophyll content was significantly higher, while the electrolyte leakage was much lower in *GhSIF1* silenced plants than in control plants (Fig. 7e & f), indicating that knock-down of *GhSIF1* gene in cotton resulted in increased salt tolerance.

## Discussion

In plants, LRR-RLKs are one of the most important membrane-anchored receptors, which transduce the apoplastic signals into symplast and then trigger the downstream responses. Various studies have shown that LRR-RLKs involve in many fundamental biological processes in plants, such as phytohormone perception, plant development, and responses to the adverse environment [14–19]. The presence of large numbers in the LRR-RLK gene family makes the functional characterization of individual member difficult due to functional redundancy. *Arabidopsis* offers an excellent model for functional characterization of LRR-RLK genes due to their relatively fewer numbers and the availability of genetic and genomic resources. We have previously identified and characterized *Arabidopsis* SIF2, a negative regulator of salt tolerance [32]. The present investigation identified a homolog of *AtSIF* gene in cotton by phylogenetic analysis and functionally characterized for its role in salt tolerance using transient gene silencing system.

### Cotton LRR-RLK gene family constitutes one of the biggest gene families in the plant kingdom

Due to their diverse and critical roles in signal transduction, plant development, photomorphogenesis, and abiotic/biotic stress responses, LRR-RLKs constitute one of the largest gene families in the plant and animal kingdoms. The present study identified 543 LRR-RLK genes and the number is much larger than that of diploid plant species *Arabidopsis* (234) and rice (309). It is also larger than paleopolyploid soybean (467) and allohexaploid wheat (531) [12, 24, 28, 30]. This high number of genes is likely due to cotton’s complex allotetraploid genome and long evolutionary history along with complex traits such as specialized fibers. In addition to the complex genome, cotton produces longest single cell in the plant kingdom composed of ~ 96% cellulose which requires precise developmental regulation.

Cultivated cotton (*G. hirsutum*) is an allotetraploid organism which is the result of the hybridization of two diploid progenitor relatives *G. arboreum* (AA) and *G. raimondii* (DD) [45]. Each of the two progenitors provided one set of 13 chromosomes to *G. hirsutum* leading to genome doubling in the cultivated *G. hirsutum* (A_t_A_t_D_t_D_t_; 2*n* = 4*×* = 52) [46]. Analysis of chromosomal location provides the information about the position of a gene on the specific chromosome. However, it does not provide information about the nature of its origin, hence we performed gene duplication analysis. Chromosomal distribution analysis showed that the distribution patterns of *LRR-RLK* genes on A-subgenome and D-subgenome were very similar (Fig. 3 & Additional file 3: Figure S1) in terms of the number and location. Nevertheless, the numbers of LRR-RLKs on A- and D-subgenomes are not equal, as A-subgenome carries 179 genes while D-subgenome carries 219 genes, which could be due to independent evolution of the parental diploid species before hybridization to form tetraploid species.

### The diversity of LRR-RLKs protein structure and functional significance

The exon-intron structure analysis showed a conservative pattern among the subclades while, the protein motif analysis revealed that protein members within the same subclade showed similar motifs, localization pattern and potentially similar functions (Additional file 3: Figure S2 A-I & Additional file 3: Figure S5 A-K). For instance, the extracellular Malectin-like domain (IPR024788) helps in recognition of and binding to Glc-N-glycan of Endoplasmic Reticulum [47]. In *Arabidopsis*, all the LRR-RLKs having N-terminal Malectin-like domain were grouped in subclade I, and several of them have been proved to be involved in biotic stress resistance [48, 49]. The extracellular N-terminal Malectin-like domain is a complex structure offering proteins the ability to recognize and bind Glc-N-glycan of Endoplasmic Reticulum, and several *Arabidopsis* LRR-RLK proteins containing this domain have been proved to be involved in biotic stress resistance [47–49]. Similarly, in cotton, N-terminal Malectin-like domain was identified in 9 LRR-RLKs, and all of them were grouped in subclade I in the phylogenic analysis. Due to the diverse functional roles of LRR-RLK proteins, these proteins have specialized domains for functional specializations. For instance, the extracellular LRR domain allows RLK to perceive a specific ligand, and the transmembrane domain allows it to firmly anchor on the plasma membrane, while the protein kinase-like domain offers its phosphorylation ability allowing it to transduce the signal to downstream signaling pathway. In the presence of a bacterial pathogen, the LRR domain of *Arabidopsis* BAK1 will instantly form a complex with the LRR domain of another LRR-RLK protein FLS2 [13]. The conformational change caused by this extracellular complex will activate the kinase domain of BAK1 to autophosphorylate itself and then transphosphorylate kinase domain of FLS2, followed by the activation of downstream signaling cascades [50].

### LRR-RLKs are involved in multiple biological processes in cotton

LRR-RLK gene family is a multigene family involved in various functions in cotton, however, only a very few *GhLRR-RLK* genes have been functionally characterized [34–37]. Biological process analysis indicated that *GhLRR-RLK*s have multiple molecular functions such as response to stimulus (502), biological regulation (476), signaling (411), metabolic process (410), developmental process (335) and reproduction (261), which underline their potential functions in plant development, environmental stress, metabolism and reproduction through signal transduction mechanism (Additional file 3: Figure S7). In addition, cotton is unique in producing highly specialized single cells called cotton fibers from the seed coat epidermal cells. These cells follow a unique developmental pattern with primary and secondary cell wall deposition leading to the deposition of ~ 96% cellulose. LRR-RLKs have been shown to be involved in the cotton fiber development as well as cell wall biosynthesis in cotton. *GhRLK1* was induced in developing cotton fibers and was predicted to be involved in the secondary cell wall synthesis in cotton fiber [34]. The RNAseq analysis of publicly available dataset (Fig. 4 & Additional file 3: Figure S11) showed that LRR-RLKs genes belonging to subclades VI_2, VIII_2, IX, X_2, X_3, X_4, XI_2, XI_3 and XI_4 are highly abundant across most of the fiber developmental stages while genes belonging to the subclades I, II, III, IV_1, IV_2, V, VI_1, VII, VIII_1, X_1, XI_1, XII_2 and XIII showed variable expression pattern. Further, the real-time RT-PCR expression analysis of 26 genes in leaf, 5 dpa fiber and 5 dpa ovule suggested that most of these genes were expressed in all three tissues, however, expression was significantly higher in leaves followed by ovules (Fig. 5). Out of the 26 genes, *CotAD_22753* was not detectable in any of these three tissue types whereas *CotAD_00571*, *CotAD_52735*, and *CotAD_71119* exhibited consistent expression across the three tissues (Fig. 5).

### *GhSIF1* is a negative regulator of salt tolerance in cotton

Due to presence of a large number of genes in the LRR-RLK gene family and functional redundancy, the complete understanding of their role in plant growth, development and stress responses are lagging behind. In *Arabidopsis*, only 35 genes have been functionally characterized [12] which indicates the complexity involved in the functional characterization of the LRR-RLK family genes. Functional analysis of these genes in tetraploid cotton with a much bigger gene family and long life cycles coupled with transformation hindrances, it will be difficult to completely characterize all the *GhLRR-RLK* genes in cotton. The present study provides a comprehensive analysis of cotton LRR-RLKs, which will help in rapid identification and characterization of cotton genes using translational research and advanced functional genomics tools. Particularly, with the information from the characterized *Arabidopsis* genes, it is possible to predict and functionally characterize the respective cotton homologous gene. We have recently identified a subfamily of AtLRR-RLK gene family (*SIF* gene family; *SIF1-SIF4*), which is shown to be involved in both biotic and abiotic stress responses. Particularly, knocking out of *SIF1* and *SIF2* significantly enhanced the salt tolerance of *Arabidopsis* [32]. Interestingly, the phylogenetic analysis using *Arabidopsis* SIF gene family showed that only one gene, *GhSIF1* has a very close evolutionary relationship with *AtSIFs* (Fig. 2). By generating highly specific VIGS construct targeting *GhSIF1*, we have functionally characterized its role in salt tolerance in cotton paving the way for rapid functional characterization of cotton genes using translational research. The transiently silenced cotton plants showed enhanced salt tolerance, indicating that *GhSIF1*, similar to *AtSIFs* in *Arabidopsis*, is a negative regulator of plant salt tolerance (Figs. 5, 6 and 7). The transient characterization is highly practical for rapid functional characterization of genes due to the recalcitrance, laborious and time-consuming stable transformation in cotton.

## Conclusions

The present investigation performed a genome-wide analysis of LRR-RLK family genes in cultivated tetraploid cotton *G. hirsutum* leading to the identification of 543 GhLRR-RLKs. Five hundred forty-two GhLRR-RLKs were grouped into 13 subclades while remaining one protein, CotAD_01838, was not assigned to any subclade. These *GhLRR-RLK* genes were distributed on all 13 chromosomes of both A and D subgenomes but at a different frequency, and a total of 42 tandem duplication events were identified involving 110 genes. Our results also indicated that each LRR-RLKs subclade has distinctive gene structure and the protein structure. Gene expression analysis and functional annotation indicated that *GhLRR-RLK*s were spatiotemporally expressed and potentially involved in various biological processes in different tissues or cell types. Genome-wide analysis and phylogenetic analysis led to the identification of six *Arabidopsis* SIF homologs in cotton. Among them, GhSIF1 has the highest conserved amino acid sequence with AtSIF subfamily. Functional studies demonstrated that the salt tolerance function of *GhSIF1* is conserved with *AtSIF1* and AtSIF2. This offers an excellent opportunity to silence the *GhSIF1* to develop salt-tolerant cotton using genome editing technologies as *GhSIF1* is a negative regulator of salt tolerance.

## Methods

### Identification of LRR-RLK gene family in *G. hirsutum*

For the in-silico identification of LRR-RLK gene family in upland cotton, *G. hirsutum* reference genome data was downloaded from the CottonGen database (https://www.cottongen.org/data/download/genome) [46, 51]. *Arabidopsis* LRR-RLK family 234 gene ids were pooled from the previous reports and their protein sequences were retrieved from TAIR10 database (https://www.arabidopsis.org/) [11, 12, 52]. A BlastP similarity search (https://blast.ncbi.nlm.nih.gov/Blast.cgi?CMD=Web&PAGE_TYPE=BlastDocs&DOC_TYPE=Download) was performed against the *G. hirsutum* reference proteome data using *Arabidopsis* LRR-RLK family protein sequences as the query at default parameters with an e-value of 10^− 10^. Non-redundant protein sequences obtained from BlastP search were analyzed for the presence of Leucine-Rich Repeats (LRRs) and kinase domain using the online hmmscan search tool (HMMER; https://www.ebi.ac.uk/Tools/hmmer/search/hmmscan) [53] and NCBI’s Conserved Domains Database (CDD; http://www.ncbi.nlm.nih.gov/Structure/cdd/wrpsb.cgi) [54]. Further, proteins with minimum of one LRR repeat and a kinase domain were analyzed for the presence of transmembrane helices using online available TMHMM server v.2.0 (http://www.cbs.dtu.dk/services/TMHMM/) [55]. Upland cotton protein sequences with minimum of 1 LRR repeat, kinase domain and transmembrane helices were classified as *GhLRR-RLK* gene family members and were used for further characterization. For the identified *GhLRR-RLK* genes, we continued to use the original gene id provided in the reference genome [46].

### Phylogenetic analysis of GhLRR-RLK proteins

To further classify into subclades based on their sequence similarity with *Arabidopsis* LRR-RLK proteins, phylogenetic analysis of GhLRR-RLK family members was performed using Molecular Evolutionary Genetics Analysis (MEGA) v6.06. LRR-RLK proteins from cotton (543) and *Arabidopsis* (234) were subjected to multiple alignment using ClustalW sequence alignment program of MEGA v6.06 [56] with default parameters. Further, a phylogenetic tree was constructed with MEGA v6.06 using Neighbor-Joining (NJ) method. Bootstrap replicates of 1000 along with other default parameters (phylogenetic reconstruction, substitution type: amino acids, model/methods: p-distance, rates among sites: uniform rates and gap missing data treatment: partial deletion) were used to create the phylogenetic tree. Based on the presence of previously classified *Arabidopsis* LRR-RLK proteins, branches were classified into 23 LRR-RLK sub-groups. Phylogenetic analysis of AtSIF family and GhLRR-RLK subclade I was performed on the phylogeny.fr server (www.phylogeny.fr) [57].

### Physical properties, gene structure and chromosomal localization analysis

The identified cotton *LRR-RLK* genes were grouped into subclades and analyzed further for detailed characterization. Gene length, protein size, location and orientation on the chromosomes were retrieved from the reference genome dataset. Other physical properties such as theoretical pI and molecular weight of the LRR-RLK proteins were calculated using the ExPASy server’s Compute pI/Mw tool (http://web.expasy.org/compute_pi/). For the chromosomal localization analysis, chromosomal coordinates of the cotton *LRR-RLK* genes were plotted on the *G. hirsutum* A- and D-subgenome specific chromosomes separately using the Mapchart 2.30 software. For the gene structure analysis, exon-intron coordinates for each *GhLRR-RLK* genes were fetched from the *.gff* file and diagrammatically represented using the Gene Structure Display Server 2.0 [58].

### Tandem duplication among cotton LRR-RLK genes

Tandem duplication among the *LRR-RLK* gene family was analyzed by comparing their position on the chromosome/scaffold. Adjacent genes with a maximum of one gene interruption were considered as tandemly duplicated genes. In some cases, adjacent genes interrupted by a maximum of two genes were also considered tandemly duplicated if they were within 1 MB region.

### Protein structure analysis, domains distribution, and annotation analysis

InterProScan domains and Blast2GO annotation analysis were conduct with 543 *G. hirsutum* LRR-RLK protein sequences using Blast2GO tool suite according to the software instruction [40]. The extracellular structure of GhLRR-RLK proteins was analyzed with Motif Alignment & Search Tool on Motif-based sequence analysis online tools [59]. Reference motifs (LRR clade domains and Malectin-like domain) were obtained from the NCBI’s Conserved Protein Domain database (https://www.ncbi.nlm.nih.gov/Structure/index.shtml). Signal peptide identification was performed on the SignalP 4.1 Server (http://www.cbs.dtu.dk/services/SignalP/) [60]. Transmembrane domain analysis was performed using the TMHMMserver V.20 on SignalP 4.1 Server.

### In-silico gene expression analysis of *GhLRR-RLKs*

Transcriptome datasets were obtained from NCBI’s Short Read Archive (SRA) database (http://www.ncbi.nlm.nih.gov/sra) for different cotton fiber developmental stages (− 3, − 1, 0, 1, 3, 5, 10, 20, and 25 dpa) and leaves from *G. hirsutum* TM-1 (Additional file 1: Table S5). Reads from different datasets were mapped on *GhLRR-RLK* family related genes using the QSeq program of DNASTAR Lasergene package (http://www.dnastar.com/t-nextgen-qseq.aspx). Hierarchically clustered heatmaps for individual sub-groups were created with the MeV (http://mev.tm4.org/#/welcome) using the self-normalized log converted RPKM (Reads per Kilobase per Million reads) values calculated by the QSeq program. Apart from this, another heatmap showing the expression of all the cotton *LRR-RLK* genes was created using the QSeq heat map option.

### Plant growth, RNA isolation, cDNA synthesis and quantitative PCR analysis

*G. hirsutum* TM-1 seeds were germinated on soil and the plants were grown under a 16 h-light/8 h-dark photoperiod at 28 °C in the growth chamber (Percival, Perry, Iowa) and moved to the green house for maturity to produce cotton fibers. Plant total RNA was isolated with Spectrum plant total RNA kit (Sigma-Aldrich, USA) from 100 mg plant sample according to the manufacturer’s instructions. The first strand cDNA was synthesized using iScript Reverse Transcription Supermix for RT-qPCR (Bio-Rad, USA) with 1 μg total RNA according to the manufacturer’s instruction. Real-time PCR was performed with FastStart Essential DNA green Master (Roche, Swiss) according to the manufacturer’s instructions. LightCycler 96 (Roche, Swiss) was used for the real-time PCR experiments. Real-time PCR results were calculated by using the ΔΔCt method [61].

### Plasmid construction and transient gene silencing

For pTRV2(*GhSIF1*) plasmid construction, a 371 bp 3’ UTR fragment of *GhSIF1* cDNA was amplified from *G. hirsutum* cDNA pool with NEBNext Q5 High-Fidelity polymerase (NEB, U.S.A). The pTRV vectors were obtained from the TAIR (https://www.arabidopsis.org/abrc/catalog/vector_3.html) [62]. The 3’ UTR region on *GhSIF1* was carefully selected to avoid off targeting of the VIGS system. The primers used to amplify the cDNA fragment were forward primer 5’-AAATCTAGATCAAATCATTAAATTTGATGCCTTTC-3′ with X*ba*I restriction site, and reverse primer 5’-AAAGAGCTCAATTCTTATTTACAAAAAAGCCATC-3′ with *Sac*I restriction site. The PCR product was then digested with *Xba*I and *Sac*I, and sub-cloned into the binary vector pTRV2 digested with the same set of enzymes, resulting in 2 × p35S/CP/*GhSIF1*/Rbz/nos. pTRV1, pTRV2(empty), and pTRV2(*GhSIF1*) plasmids were then mobilized into *Agrobacterium tumefaciens* strain GV3101 for virus-induced gene silencing. Virus-induced gene silencing of cotton was performed following the published protocol [63].

### Determination of chlorophyll content and electrolyte leakage measurements

For determination of chlorophyll content, 300 mg of youngest leaf samples were collected from cotton plants from the growth chambers. The leaf samples were then sliced into small pieces and ground to fine powder using liquid nitrogen, which was then transferred to 15 ml Falcon tube with 5 ml of 80% acetone for chlorophyll extraction. After 30 min of incubation under room temperature, the falcon tubes were centrifuged at 4 °C for 15 min at 3000 rpm, and the supernatant was then transferred to 50 ml falcon tube with 10 ml 80% acetone and kept in the dark until chlorophyll content was determined.

Absorbance of the extract was measured at 645 nm and 663 nm by using a spectrometer, and the chlorophyll concentrations are calculated as following equation:

$$ {\displaystyle \begin{array}{l}\mathrm{Chlorophyll}\ \mathrm{a}\ \mathrm{content}\ \left(\mathrm{mg}/\mathrm{g}\right)=\left(12.7\times {\mathrm{A}}_{663}-2.69\times {\mathrm{A}}_{645}\right)\times \mathrm{V}/\mathrm{W}/1000\\ {}\mathrm{Chlorophyll}\ \mathrm{b}\ \mathrm{content}\left(\mathrm{mg}/\mathrm{g}\right)=\left(22.9\times {\mathrm{A}}_{645}-4.86\times {\mathrm{A}}_{663}\right)\times \mathrm{V}/\mathrm{W}/1000\\ {}\mathrm{Chlorophyll}\ \left(\mathrm{a}+\mathrm{b}\right)\ \mathrm{content}\ \left(\mathrm{mg}/\mathrm{g}\right)=\left(8.02\times {\mathrm{A}}_{663}-20.20\times {\mathrm{A}}_{645}\right)\times \mathrm{V}/\mathrm{W}/1000\end{array}} $$

Where: V = volume of the extract (ml); W = fresh weight of the leaf samples (mg).

For the determination of electrolyte leakage, fresh leaf disc was cut from the youngest leaf and immersed in 5 ml of deionized water. The sample was then incubated at 32 °C for 2 h, and the conductivity value was measured using a conductivity meter (Fisher Scientific) and signed as EL1. Then the sample was boiled at 95 °C–100 °C for 20 mins, and the conductivity value (EL2) was measured after the sample reached room temperature.

### Statistical analysis

Student’s t-test was used to determine the statistically significant difference between the means from different data groups. *P* < 0.05 was statistically significant and marked as *. *P* < 0.01 was statistically highly significant and marked as **.

## Additional files

Additional file 1: **Table S1**. *GhLRR-RLK*s gene list. **Table S2**. Gene Localization and duplication Data. **Table S3**. InterProScan analysis. **Table S4**. Motif composition for each subclade. **Table S5**. List of SRA datasets downloaded for expression analysis. **Table S6**. Expression analysis. (XLSX 857 kb)

Additional file 2: **Data S1.** Protein alignment of AtSIFs and GhLRR-RLKs. (PDF 2711 kb)

Additional file 3: **Figure S1.** *GhLRR-RLK*s Chromosomal distribution. **Figure S2.** Exon-intron analysis of *GhLRR-RLK*s. **Figure S3.** Protein size distribution analysis of GhLRR-RLK. **Figure S4.** InterProScan domains distribution of GhLRR-RLKs. **Figure S5.** Protein structure and domain composition of GhLRR-RLKs. **Figure S6.** Extracellular motif composition. **Figure S7.** Blas2GO annotation statistics. **Figure S8.** Cellular component analysis of *GhLRR-RLK*s. **Figure S9.** Biological processes analysis of *GhLRR-RLK*s. **Figure S10.** Molecular function analysis of *GhLRR-RLK*s. **Figure S11.** Expression analysis of *GhLRR-RLK*s. (PPTX 7480 kb)

## Abbreviations

AA: Amino acid residues
BAK1: Brassinosteroid Insensitive 1-associated receptor kinase 1
BKK1: BAK1-Like 1
CDS: Coding Sequence
CR4-class: CRINKLY4 class RLK
DPA: Day post anthesis
FLS2: Flagellin Sensitive 2
KD: Protein kinase-like domain
LRR-RLKs: Leucine Rich Repeats-RLKs
MEGA: Molecular Evolutionary Genetics Analysis
NJ: Neighbor-Joining
pI: Isoelectric point
PR5-RLK: PR5-Like RLK
RLKs: Receptor-like protein kinases
SERKs: Somatic Embryogenesis Receptor Kinase
SIF: Stress Induced Factor
SRA: Short Read Archive
S-RLK: S-domain RLK
TDE: Tandem duplication event
TRV: Tobacco Rattle Virus
UTR: Untranslated Region
VIGS: Virus-induced gene silencing
WAK: Wall Associated Kinase
