# Supplementary material for: Genome-wide identification and characterization of LRR-RLKs reveal functional conservation of the SIF subfamily in cotton (Gossypium hirsutum)
Source: BMC Plant Biol. 2018 Sep 6;18:185. doi: 10.1186/s12870-018-1395-1 (PMC6128003; doi:10.1186/s12870-018-1395-1)
Supplement: Supplementary file 2 — Data S1. Protein alignment of AtSIFs and GhLRR-RLKs. (PDF 2711 kb) [file 12870_2018_1395_MOESM2_ESM.pdf]

```
CotAD_57195      ------------MVSFTQFCILYFYFIASVVS-QVSEFISIDCGS---TSNYTDPSTGLS   44
CotAD_44233      -------MEMEKRLLFSLLLFSLLLLLDSSSA-QMQGFVSLNCGG---DGNFTDE-LGLL   48
CotAD_52119      -------MEMEKRLLFSLLLFSLLLLLDSSSA-QMQGFVSLNCGG---DGNFTDE-LGLS   48
CotAD_74481      ------------------------------------------------------------   0
CotAD_21855      ------------------------------------------------------------   0
CotAD_74959      ------------------------------------------------------------   0
AtSIF3           -------MESH-RVFVAT--F--MLILHLVQAQDQPGFINVDCGLLPRDSPYNALGTGLV   48
AtSIF1           MWTVRRTMERH-CVFVTI--F--VLILHLVQAQNQTGFISVDCGLSPPESPYNAPQTGLT   55
AtSIF2           -------MERH-CVLVAT--F--LLMLHIVHAQDQIGFISVDCGLAPRESPYNEAKTGLT   48
AtSIF4           -------MERH-FVFIAT--Y--LLIFHLVQAQNQTGFISVDCGLSLLESPYDAPQTGLT   48
CotAD_41732      -------MEKTKHYFIILFII--FTLAVVVHAQNQTGFISLDCGL-PAGSSYIDSITGIN   50
CotAD_31444      -----------MFVGYLTPLL--LSLAVLVHGQLQTGFISIDCGS-PVNFNFVDVDTGIS   46
CotAD_06671      -----------MFFGYLTPLL--LSLAVLVHGQLQT-----------------DVDTGIS   30


CotAD_57195      WSSDMDIMKHGRQVKVENPN----G--NSMQFRTRRDFPSDNKKYCYNLST--KERRRYL   96
CotAD_44233      WSSD-DFLNYGETASISVSN----E--TRQQYMSLRHFPADSKKYCYKLNV--VSRTRYL   99
CotAD_52119      WSSD-YFLNYGETASISVSN----E--TRQQYMSLRHFPADSKKYCYKLNV--VSRTRYL   99
CotAD_74481      ------------------------------------------MKNCYTLKLEQATNNSFR   18
CotAD_21855      ------------------------------------------------------------   0
CotAD_74959      ------------------------------------------------------------   0
AtSIF3           YTSDVGLVSSGKTGKIAKEF----EENNSTPNLTLRYFPD-GARNCYNLNV--SRDTNYM   101
AtSIF1           YTSDTGLINTGKTGRIAKDF----EPFVDKPALTMRYFPD-GIRNCYNLNV--TRDTNYL   108
AtSIF2           YTSDDGLVNVGKPGRIAKEF----EPLADKPTLTLRYFPE-GVRNCYNLNV--TSDTNYL   101
AtSIF4           YTSDADLVASGKTGRLAKEF----EPLVDKPTLTLRYFPE-GVRNCYNLNV--TSDTNYL   101
CotAD_41732      YTSDEPYIQTGTTHSLSRFN----SSIQQQFLENLRSFPQ-GVRHCYQLNL--TKGEKYL   103
CotAD_31444      YTPDGAYVSTGINNNISSEYAYPNNPNLAYPLSDLRSFPD-GNKNCYTLTPAAAKGSLYL   105
CotAD_06671      YTLDGAYVSTGINNNISSEYAYPNNPNLAYPLSDLRSFPD-GNKNCYTLTPAVAKGSLYL   89


CotAD_57195      VRATFQYGSPANVDAYPKFEIYLDATQWATVTIQDGSRKYVNEMIIRAPSDSIDVCICCA   156
CotAD_44233      VRATFLYGNFDNNNVYPKFDISLGATHWSTIVISDANTIEKAELIFLATSPTISVCLSNA   159
CotAD_52119      VRATFLYGNFDNNNVYPKFDISLGATHWSTIVISDANTIEKAELIFLATSPTISVCLSNA   159
CotAD_74481      IRASFAYGNYDRKNKIPKFDLYLGVNYWTTVNLTN-FEGFFYEIIHVFPADTEYVCLANT   77
CotAD_21855      ------------------------------------------------------------   0
CotAD_74959      ------------------------------------------------------------   0
AtSIF3           IKATFVYGNYDGHKDEPNFDLYLGPNLWATVSRS----ETVEEIIHVTKSDSLQVCLAKT   157
AtSIF1           IKATFVYGNYDGLNVDPNFDLYLGPNLWTTVSSN----DTTEEIIHVTKFNSLQICLVKT   164
AtSIF2           IKATFVYGNYDGLNVGPNFDLYFGPNLWTTVSSN----DTIKEIIHVTKTNSLQVCLIKT   157
AtSIF4           IKATFVYGNYDGLNVGPNFNLYLGPNLWTTVSSN----DTIEEIILVTRSNSLQVCLVKT   157
CotAD_41732      IRASFMYGNYDENDENPEFDLYLGPNLWASMVFENSTSVVVKEIIYVVKARYLHVCVVNT   163
CotAD_31444      LRASFLYGNYDGEDKLPEFDLYLDANLWSSIKFGNAFDVITTEIISASVSDTISVCLVNK   165
CotAD_06671      LRASFSYGNYDGEDKLPEFDLYLDVNLWSSIKFSNASDVITMKIIGASVSDTISVCLVNK   149
```

```
CotAD_57195      TTGFPFISTLELRPLNLSMYATDYEGDFFLNVAARVNFGALTKDLVRYPDDPYDRFWESD   216
CotAD_44233      TTGQPFISTLELRQFNGSIYYTPFEENFYLGVSARINFGADSEAPVRYPDDPFDRIWESD   219
CotAD_52119      TTGQPFISTLELRQFNGSIYYTPFEENFYLGVSARINFGADSEAPVRYPDDPFDRIWESD   219
CotAD_74481      GTGTPFISALEIRPSNISTYGNGSSLELINIGLYDLSSYPGQSPGVRYKDDIYDRVWYPS   137
CotAD_21855      ------------------------------------------------------------   0
CotAD_74959      ------------------------------------------------------------   0
AtSIF3           GDFIPFINILELRPLKKNVYVTESGSL---KLLFRKYFS-DSGQTIRYPDDIYDRVWHAS   213
AtSIF1           GISIPFINVLELRPLKKNVYATQSGSL---KYLFRMYVS-NSSRRIRFPDDVYDRKWYPI   220
AtSIF2           GISIPFINVLELRPMKKNMYVTQGESL---NYLFRVYIS-NSSTRIRFPDDVYDRKWYPY   213
AtSIF4           GISIPFINMLELRPMKKNMYVTQSGSL---KYLFRGYIS-NSSTRIRFPDDVYDRKWYPL   213
CotAD_41732      GKGIPFISALESRLLSNVTYNTDSNTQ-ALEFFLRIDFGSALNSSFRFPEDIYDRIWQPY   222
CotAD_31444      GLGIPFISALELRPLNNSIYGTELGSPVSLVLFERFDIGYT-KGTGRYNDDIYDRIWSPY   224
CotAD_06671      GLGIPFISTLELRPLNNSIYGTELGSLVSLVLFERFDIEYT-NGTGRYNDDIYDRIWSPY   208


CotAD_57195      LDRRQNFLVGVAPGTARISTSKNVD--I-RTREYPPVKVMQTAVVGTEGELSYR--LNLE   271
CotAD_44233      SLKRANYLVDVAAGTVKVSTNKSIY--I-SSSEMPPEKVMQTAVVGTNGSLTYR--LNLD   274
CotAD_52119      SLKRANYLVDVAAGTVKVSTNKSIY--I-SSTEMPPEKVMQTAVVGTNGSLTYR--LNLD   274
CotAD_74481      QLPRS----------VPINTSSDIDTQGSGNLYKLPVEVLRTAVRPFNGSNSLSYSSSFA   187
CotAD_21855      ------------------------------------------------------------   0
CotAD_74959      ------------------------------------------------------------   0
AtSIF3           FLENN---------WAQVSTTLGVN--V-TDNYDLSQDVMATGATPLNDSETLNITWNVE   261
AtSIF1           -FQNS---------WTQVTTNLNVN--I-STIYELPQSVMSTAATPLNANATLNITWTIE   267
AtSIF2           -FDNS---------WTQVTTTLDVN--T-SLTYELPQSVMAKAATPIKANDTLNITWTVE   260
AtSIF4           -FDDS---------WTQVTTNLKVN--T-SITYELPQSVMAKAATPIKANDTLNITWTVE   260
CotAD_41732      -RRND---------LTTINSSSSLI--S-NSYFDPPLVAMMTASIPMNGSQSLNFTVRDS   269
CotAD_31444      -NSPS---------WNIVSTSSEIN--NNENGYRAPLEVIRTAAVPRNGSDRLEFSWTAD   272
CotAD_06671      -NSPS---------WNIVSTSSEIN--SNENGYRAPLEVIRTSAVPRNGSDRLEFSWTAD   256


CotAD_57195      GFPANARAYAYFAEIEDLAPNETRKFQLREPYIPDYSNAVVNIAENANGSYTLYEPSYMN   331
CotAD_44233      GFPGFGWAFTYFAEIEDLAPDESRKFRLVLPGQPDLSKAVVNIQENAQGKYRVYEPGYPN   334
CotAD_52119      GFPGFGWAFTYFAEIEDLAPDESRKFRLVLPGQPDLSKAVVNIKENAQGKYRVYEPGYPN   334
CotAD_74481      -SSYDYNLYFHFAELEFF-EDKQREISITVNGI-T---------------QGPFTLEYLK   229
CotAD_21855      ------------------------------------------------------------   0
CotAD_74959      ------------------------------------------------------------   0
AtSIF3           PPTTKVYSYMHFAELETLRANDTREFNVMLNGNDL---------------FGPYSPIPLK   306
AtSIF1           PPTTPFYSYIHFAELQSLRANDTREFNVTLNGEYT---------------IGPYSPKPLK   312
AtSIF2           PPTTKFYSYMHFAELQTLRANDAREFNVTMNGIYT---------------YGPYSPKPLK   305
AtSIF4           PPTTQFYSYVHIAEIQALRANETREFNVTLNGEYT---------------FGPFSPIPLK   305
CotAD_41732      DPDVEFYFYMHVVELEELQANQTREFNIYLNDNFW---------------YGPFSPMYLQ   314
CotAD_31444      DDASKFYVYMYFAEVQ-LAKNQTRKFNISWNGSLM---------------FGPLVPRYLF   316
CotAD_06671      DDASKFYVYMYFAEVQQLAKNQTRKFNISWNGSLI---------------FGPLVPRYLF   301
```

```
CotAD_57195      V----------TLDFVLSFTFSKTNDSTEGPLLNAMEISKYQQIAA-KTERQDVTALNAI   380
CotAD_44233      I----------SLPFVLSFRFGKTSDSSKGPLLNAIEINRYLEKND-GSV--DGMVIANV   381
CotAD_52119      I----------SLPFVLSFRFGKTSDSSKGPLLNAIEINRYLEKND-GSV--DGMVIANV   381
CotAD_74481      PLSISFQRLR-TVEGNITFTISTTMESGLPPILNALEYYQLKQFTDSATDPSDVNAIMSI   288
CotAD_21855      ------------------------MESGLPPILNALEYYQLKQFTDSTTDPSDA------   30
CotAD_74959      ------------------------MESGLPPILNALEYYLLEQFTDSAADPSDG------   30
AtSIF3           TETETNLKPEECEDGACILQLVKTSKSTLPPLLNAIEAFTVIDFLQVETDEDDAAAIKNV   366
AtSIF1           TETIQDLSPEQCNGGACILQLVETLKSTLPPLLNAIEAFTVIDFPQMETNEDDVTGINDV   372
AtSIF2           TETIYDKIPEQCDGGACLLQVVKTLKSTLPPLLNAIEAFTVIDFPQMETNGDDVDAIKNV   365
AtSIF4           TASIVDLSPGQCDGGRCILQVVKTLKSTLPPLLNAIEAFTVIDFPQMETNENDVAGIKNV   365
CotAD_41732      GETILS--SLSVKGGQ--FSMESTRSSTHPPIINAFEAYKVKELVESQTVETEVNAMMNI   370
CotAD_31444      AATISN--SEAFTGKEHHISIYKTVDANLPPILNAVEIYMAKQLDELPTFSEDVAAVLNI   374
CotAD_06671      AATILN--SEAFTGKEHHISIYKTADANLPPILNAIEVYMAKQLDELPTLSEDVAAVVNI   359
                                          .:   *::**.*     :     :   :


CotAD_57195      RNMSAESVWVNEGGDPCVPTHWEWV--NCSPT---PPPRITKIALSGRNLKGQIPSEINH   435
CotAD_44233      VALYPSADWAQEGGDPCLPVPWSWV--RCNSD---RQPKIVSILLSSQNLTGNIPSEFTK   436
CotAD_52119      VALYPSADWAQEGGDPCLPVPWSWV--QCNSD---RQPKIVSILLSSKNLTGNIPSDFTK   436
CotAD_74481      KHTY---NRDDWQGDPCLPKGYSWSGLSCHFG---SPPRIISLNLSSSKLTGEISPSLAH   342
CotAD_21855      --YI---NRDDWQGDPCLPKGYSWSGLSCYFG---IPPRIISLNLSSSKLTGEISPSLAH   82
CotAD_74959      --TL---LFD--------------------SG---TPHLILCRNLSSSKLTGEISPSLAH   62
AtSIF3           QNAYGLINRSSWQGDPCVPKQYSWDGLKCSYSD-STPPIINFLDLSASGLTGIIAPAIQN   425
AtSIF1           QNTYGL-NRISWQGDPCVPKQYSWDGLNCNNSDISIPPIIISLDLSSSGLNGVITQGIQN   431
AtSIF2           QDTYGI-SRISWQGDPCVPKLFLWDGLNCNNSDNSTSPIITSLDLSSSGLTGSITQAIQN   424
AtSIF4           QGTYGL-SRISWQGDPCVPKQLLWDGLNCKNSDISTPPIITSLDLSSSGLTGIITQAIKN   424
CotAD_41732      KSMYRL--KKNWEGDPCAPRTYSWEGLDCSYDD-LDPPRIISLNLSSSGLSGEISPYIGN   427
CotAD_31444      KTAYQV--NKGWVGDPCGPKNYTWEGLECNYSV-SLPPRIISLNLTSSGLSGIISASFAN   431
CotAD_06671      RTAYQV--NKGWVGDPCGPKNYTWEGLECNYSV-SLPPRIISLNLTSSGLSGIISASFAN   416
                                                        *    *:.  *.* *   : :


CotAD_57195      FSGEIPAALLSKK----ISFNYEGNPG----LH----------NEAQRKLRFK-LILGAS   536
CotAD_44233      LTGRVPSGLLSKN----LALNYAGNIN----LH----------EGGGRAKHLSTIIVGSS   538
CotAD_52119      LTGRVPSGLLSKN----LALNYAGNIN----LH----------EGGG--KHLSTIIVGSS   536
CotAD_74481      LKGPIPQSLKDKSDHGSLLLSFDENQTSCHRDPCEG-----------NSKKFI-VPVVVS   403
CotAD_21855      LKGPIPQSLKDKSDHGSLLLSFDENQTSCQRDHCEG-----------NSKKFI-VPVVVS   167
CotAD_74959      LKGPIPQSLKDKSDHGSLLLSLDENQTSCQRDPCEG-----------NRKKFI-VPVVVS   147
AtSIF3           LSGPVPASLLQKKG---LMLHLDDNP----HILCTTGSCMHKGEG--EKKSII-VPVVAS   512
AtSIF1           LTGSVPLSLLQKKG---LKLNVEGNP----HLLCTDGLCVNKG-DGHKKKSII-APVVAS   519
AtSIF2           LSGSVPPSLLQKKG---MKLNVEGNP----HLLCTADSCVKKGEDGHKKKSVI-VPVVAS   513
AtSIF4           LSGSVPPSLLQKKG---MKLNVEGNP----HILCTTGSCVKKKEDGHKKKSVI-VPVVAS   513
CotAD_41732      LNGSVPAGLIDRINRRFLQLNVEGNQIPCTWEPCTTGTKT-------SENSVV-VTVVAS   516
CotAD_31444      LSGSVPTELVDRSKAGLLTLRVDEQN------LCSSGSCK-------NKKNVV-VPVVAS   514
CotAD_06671      --------------------VDEQN------LCSSGSCK-------NKKNVV-VPVVAS   472
                                         :                          .    :  *
```

```
CotAD_57195         MEELTELWLDFNSLTGPLPDMSNLINLEILHLENNKLSGSLPLYLGRLPNLQALYIQNNS   495
CotAD_44233         LSGLVELWLDGNSLTGPIPDFTGCVDLRIIHLENNHLTGELPSSLMNLPNLRELYVQNNM   496
CotAD_52119         LSGLVELWLDGNSLTGPIPDFTGCVDLRIIHLENNHLTGELPSSLMNLPNLRELYVQNNM   496
CotAD_74481         LL-----------------------ELQS------------------------INISGNK   355
CotAD_21855         LL-----------------------ELESIDLSYNDLTGSVPDVLATLPKLKSLNISGNK   119
CotAD_74959         LL-----------------------ELESIDLSYNDLTGSVPDVLATLPKLKSLNISGNK   99
AtSIF3              LT-----------------------HLEILALSNNNLTGEVPEFLADLKSIMVIDLRGNN   462
AtSIF1              LT-----------------------HLQYLDLSDNNLTGDIPKFLADIQSLLVINLSGNN   468
AtSIF2              LT-----------------------NLQELDLSDNNLTGEIPDFLGDIKSLLVINLSGNN   461
AtSIF4              LT-----------------------HLQILDLSDNNLTGEVPEFLADIKSLLVINLSGNN   461
CotAD_41732         LT-----------------------QLQYLDLSNNNLTGGVPEFLTQLQFLTLINLQGNA   464
CotAD_31444         IT-----------------------SMESLDLSNNHLSGPVPEFLKELKSLKFMNLEGNQ   468
CotAD_06671         IS-----------------------SMESLDLSNNHLSGPVPEFLKELKSLNFL------   447
                    :                         :.                         :

CotAD_57195         IGVLAVLLILFLGSLILLRNFRRKMSHQKCDENGNSTQPGTKQSTACSIARGGHLLDEGV   596
CotAD_44233         VGAAFLLISTILSCIILRKG-KKKHEQEQF----HHPLPIQGQRIA----SSLSNAPTEA   589
CotAD_52119         VGAAFLLISTILSCIILRKG-KKKHEQEQF----HHPLPIQGQRIA----SSLSNAPTEA   587
CotAD_74481         IIAAVAL--VI-LLSVLFIFHRKRKRK-QTE----VV--------NNTTASNKEEFLKSK   447
CotAD_21855         IIAAVAL--VI-LLSVLFIFHRKRKRK-QTE----VV--------NNTTASNKEGFLKSK   211
CotAD_74959         IIAAVAL--VI-LLSVLFIFHRKRKRK-QTE----VV--------NNTIASNKEGFLKSK   191
AtSIF3              IVSLAVI--I--GALILFLVFRKKKAS-KVE----GTLPSYMQASDGRSPRSSEPAIVTK   563
AtSIF1              IASIAIL--I--GALVLFFVLKKKT---QSK----GPPAAYVQASNGRSRRSAEPAIVTK   568
AtSIF2              IASIAVL--I--GALVLFFILRKKKSP-KVE----GPPPSYMQASDGRSPRSSEPAIVTK   564
AtSIF4              IASIAVL--I--GALVLFLILRKKRSP-KVE----GPPPSYMQASDGRLPRSSEPAIVTK   564
CotAD_41732         VASVLSV--VIIVSALLWWFKRIKTSG-KLV----LTP--------------RKLFQELK   555
CotAD_31444         LLSALVL--C-IALIILWRLRRKQKWE-ADT----SNG--------------EGRPLPSK   552
CotAD_06671         LLSALVL--C-VALIMLWRLRRKQKSE-ADT----SNG--------------EGRPLPSK   510
                    :     :        :*    : :

CotAD_57195         AYCISLSDLEEATNNFTKKIGKGSFGSVYYGKMKDGKEVAVKTMADSSSHLNKQFVTEVA   656
CotAD_44233         ANCFTLPEIEDATNNFEKKIGSGGFGVVYYGKMKDGKEIAVKVLTSNSFQGKREFTNEVS   649
CotAD_52119         ANCFTLPEIEDATNNFEKKIGSGGFGVVYYGKMKDGKEIAVKVLTSNSFQGKREFTNEVS   647
CotAD_74481         NQPFTYSEIVTITANFKTGIGEGGFGKVFHGHLNDGTPVAVKLLSSSSKQGYKEFQAEAQ   507
CotAD_21855         NQPFTYSEIVTITANFETGIGEGGFGKVFHGHLNDGTPVAVKLLSSSSKQGYKEFQAEAQ   271
CotAD_74959         NQPFTYSEIVTITANFKTGIGEGGFGKVFHGHLNDGTPVAVKLLSSSSKQGYKEFQAEVR   251
AtSIF3              NKRFTYSQVVIMTNNFQRILGKGGFGIVYHGFVNGVEQVAVKILSHSSSQGYKQFKAEVE   623
AtSIF1              NKRFTYSEVMQMTNNFQRVLGKGGFGIVYHGLVNGTEQVAIKILSHSSSQGYKQFKAEVE   628
AtSIF2              NRRFTYSQVAIMTNNFQRILGKGGFGMVYHGFVNGTEQVAVKILSHSSSQGYKEFKAEVE   624
AtSIF4              NRRFSYSQVVIMTNNFQRILGKGGFGMVYHGFVNGTEQVAVKILSHSSSQGYKQFKAEVE   624
CotAD_41732         NRQFTYSDIQRITNNFEKVIGKGGFGTVYHGFLD-DNQVAVKMLSKTSFQGYKQFEAEVE   614
CotAD_31444         NRQFTYAEVLNITNNFQDVIGKGGFGTVFRGNMKDGTQVAVKMLSTSSKQGSKEFQAEAE   612
CotAD_06671         NRQFTYAEVLNITNNFQDVIGKGGFGTVFRGNMKDETQVAVKMFSTSSKQGLKEFQAEAE   570
                       ::  ::   * **   :*.*.** *: * :.    :*:* :: .* :  ::*  *.
```

```
CotAD_57195      LLSRIHHRNLVPLIGYCEEAHQRILVYEYMHNGTLRDHIH---GSVN-QKPLDWLARLKI   712
CotAD_44233      LLSRIHHRNLVQFLGYCQEDGRSMLVYEFMHNGTLKEHLY---GPLTRERGIGWIKRLEI   706
CotAD_52119      LLSRIHHRNLVQFLGYCQEDGRSMLVYEFMHNGTLKEHLY---GPLTRERGISWIKRLEI   704
CotAD_74481      SLMIVHHKNLVSLI----------------------------ATK-TTVLNWNQRLQI   536
CotAD_21855      SLMIVHHKNLVSLIGYCKEDDHMALVYEFMSNGNLRQHLFCMDDATN-TTVLNWNQRLQI   330
CotAD_74959      CVAELERGGSERFS----------VVT--------------------------------   268
AtSIF3           LLLRVHHKNLVGLVGYCDEGENMALIYEYMANGDLKEHMS---GTRN-RFILNWETRLKI   679
AtSIF1           LLLRVHHKNLVGLVGYCDEGENLALIYEYMANGDLKEHMS---GTRN-HFILNWGTRLKI   684
AtSIF2           LLLRVHHKNLVGLVGYCDEGENMALIYEYMANGDLKEHMS---GTRN-RFTLNWGTRLKI   680
AtSIF4           LLLRVHHKNLVGLVGYCDEGDNLALIYEYMANGDLKEHMS---GTRN-RFILNWGTRLKI   680
CotAD_41732      LLLRVHHRNLTALIGYCDDGTETGLIYEFMAEGNLAEYLS---DSS--ISVLNWEGRLGI   669
CotAD_31444      LLMRVHHRNLASFIGYCDEGSNMALIYEYMANGNLKDYLS---YKS--SNPLSWEMRLRI   667
CotAD_06671      LLMRVHHRNLASLIGYCDEGSNMALIYEYMANGNFEGL----------------------   608
                  :  :.: .   :

CotAD_57195      AEDAAKGLEYLHTGCNPSIIHRDVKTSNILLDINMRAKVSDFGLSRQAE-EDLTH-VSSV   770
CotAD_44233      AEDAAKGIEYLHTGCIPAIIHRDLKTSNILLDKHMRAKVSDFGLSKLAV-DGASH-VSSI   764
CotAD_52119      AEDAAKGIEYLHTGCIPAIIHRDLKTSNILLDKHMRAKVSDFGLSKLAV-DGASH-VSSI   762
CotAD_74481      AVDAAQGLDYLHNGCKPPIVHRDLKPSNILLTENMQAKIADFGLSRAFSMDTSTSSISTA   596
CotAD_21855      AADAAQGLDYLHNGCKPPIVHRDLKPSNILLTENMRAKIADFGLSRAFSTDTSTSSISTT   390
CotAD_74959      ------------------------------------------------------------   268
AtSIF3           VIDSAQGLEYLHNGCKPLMVHRDVKTTNILLNEHFEAKLADFGLSRSFPIGGETH-VSTV   738
AtSIF1           VVESAQGLEYLHNGCKPLMVHRDIKTTNILLNEQFDAKLADFGLSRSFPIEGETH-VSTA   743
AtSIF2           VVESAQGLEYLHNGCKPPMVHRDVKTTNILLNEHFQAKLADFGLSRSFPIEGETH-VSTV   739
AtSIF4           VIESAQGLEYLHNGCKPPMVHRDVKTTNILLNEHFEAKLADFGLSRSFLIEGETH-VSTV   739
CotAD_41732      ALEAAQGLEYLHHGCKPPIIHRDVKSSNILLTDNLQAKLSDFGLSKSFEGGSHVS---TV   726
CotAD_31444      AIHAAQGLEYLHHGCKPPIIHRDVKTANILLSENMDAKIADFGLSRAIPSDDHSDVIITT   727
CotAD_06671      -------------------SLIDVKTANILLSEKMDAKIADFGLSRAIPSDDHSDVIITT   649


CotAD_57195      ARGTVGYLDPEYYASQQLTEKSDVYSFGVVLLELISGKKPVSVEDFGPE-LNIVHWARSL   829
CotAD_44233      VRGTVGYLDPEYYISQQLTDKSDVYSFGVILLELMSGQEAISNESFGVNCRNIVQWAKLH   824
CotAD_52119      VRGTVGYLDPEYYISQQLTDKSDVYSFGVILLELMSGQEAISNESFGVNCRNIVQWAKLH   822
CotAD_74481      LAGTPGYIDPELHASGTLNKKSDVYSFGIILFELITVLPVQITNT--EHIMHIVDWVTPM   654
CotAD_21855      LAGTPGYIDPELHASGTLNKKSDVYSFGIILFELITGLSVQIKNT--ERIMHIVEWVTPM   448
CotAD_74959      ------------------------------------------------------------   268
AtSIF3           VAGTPGYLDPEYYKTNRLTEKSDVYSFGIVLLEMITNRPVIDQ-S--REKPYISEWVGIM   795
AtSIF1           VAGTPGYLDPEYYRTNWLTEKSDVYSFGVVLLEIITNQPVIDP-R--REKPHIAEWVGEV   800
AtSIF2           VAGTPGYLDPEYYKTNWLTEKSDVYSFGIVLLELITNRPVIDK-S--REKPHIAEWVGVM   796
AtSIF4           VAGTPGYLDPEYHRTNWLTEKSDVYSFGILLLEIITNRHVIDQ-S--REKPHIGEWVGVM   796
CotAD_41732      IAGTPGYLDPEYSTTNRLTEKSDVYSFGVVLLEIITNRPVIARTI--DEPTHISRWVGSM   784
CotAD_31444      VMGTPGYLDPEYYNSRKLNEKSDVFSFGIVLLELITGQNAVIK-K--DESIHIVNWVSPL   784
CotAD_06671      VMGTAGYLDPEYFNSRKLNEKSDVVSLGIVSLELITGQNAIIK-K--DESIHIVHWVSPL   706
```

```
CotAD_57195     IRKGDVISIVDPFLVGNVKIESIWRIAEVAIQCVEQHGYSRPKMQEIILAIQDAMKIEKG   889
CotAD_44233     IESGDIQGIIDPYLRNDYDIQSMWKIAEKALNCVQPHGHMRPSISEVLKEIQDAILIEKE   884
CotAD_52119     IESGDIQGIIDPYLRNDYDIQSMWKIAEKALNCVQPHGHMRPSISEVLKEIQDAILIEKE   882
CotAD_74481     IEEGDIRKIIDPRLGSGFDVNAAWKAVEIAMWCAQRTSKQRPDISQVIAELKECIGRNDQ   714
CotAD_21855     IEEGDIRKIIDPRLGNDFDVNAAWKAVEIAMWCAQRTSKQRPDISQVIAELKGCIGRNDE   508
CotAD_74959     ------------------------------------------------------------   268
AtSIF3          LTKGDIISIMDPSLNGDYDSGSVWKAVELAMSCLNPSSTRRPTMSQVLIALNECLVSENS   855
AtSIF1          LTKGDIKNIMDPSLNGDYDSTSVWKAVELAMCCLNPSSARRPNMSQVVIELNECLTSENS   860
AtSIF2          LTKGDINSIMDPNLNEDYDSGSVWKAVELAMSCLNPSSARRPTMSQVVIELNECIASENS   856
AtSIF4          LTKGDIQSIMDPSLNEDYDSGSVWKAVELAMSCLNHSSARRPTMSQVVIELNECLASENA   856
CotAD_41732     LSNGDIENIVDSRLKGDFEINSVWRAIEVAMACLSSASTKRPTMNHVVTELSECLVAEIN   844
CotAD_31444     IEREDIGSIVDQRLHGEFDVSSAWKALEVAMACTRPKSLHRATMSTVLTELNQCLAMELS   844
CotAD_06671     IEREDIGSIVDQRLHGEFDVSSAWKALQVAMACTRPKSLHRATMSTVLTELNQCLAMELS   766


CotAD_57195     DEG----NAKLACGS-SRGQSSRKT---L--LASFLEIESPDLSNGSLVPSAR  932
CotAD_44233     GMMVREGNSDEMSRN-SANSSYNPGSLDMIGTDNYLSL-----DESIARPVAR  931
CotAD_52119     SMMVREGNSDEMSRN-SANSSYNPGSLDMIGTDNYLSL-----DESIARPVAR  929
CotAD_74481     NVQ------IEMT-------------------------SLEMASYSYTLPRAR  736
CotAD_21855     NVQ------SEMT-------------------------SLEMASYSYTFPEAR  530
CotAD_74959     -----------------------------------------------------  268
AtSIF3          RGG----ASRDMDSKSSLE--------------VSLTFDTDV------SPMAR  884
AtSIF1          RGG----AIRDMDSEGSIE--------------VSLTFGTEV------TPLAR  889
AtSIF2          RGG----ASRDMDSKSSIE--------------VSLTFDTEL------SPTAR  885
AtSIF4          RGG----ASRDMESKSSIE--------------VSLTFGTEV------SPNAR  885
CotAD_41732     RTR----GVDEDESRDMMSMA-------------SLNCDSDI------IPLSR  874
CotAD_31444     HNR----ETKERFSEEIYIGPYN----------SSEVCSTSTTSYSITRPFAR  883
CotAD_06671     HNR----ETKERFSEEIYSGSYH----------SSEVCSTSTASYSITSLFAR  805
```
