# Supplementary material for: Genome-wide identification and characterization of LRR-RLKs reveal functional conservation of the SIF subfamily in cotton (Gossypium hirsutum)
Source: BMC Plant Biol. 2018 Sep 6;18:185. doi: 10.1186/s12870-018-1395-1 (PMC6128003; doi:10.1186/s12870-018-1395-1)

## Slide 1
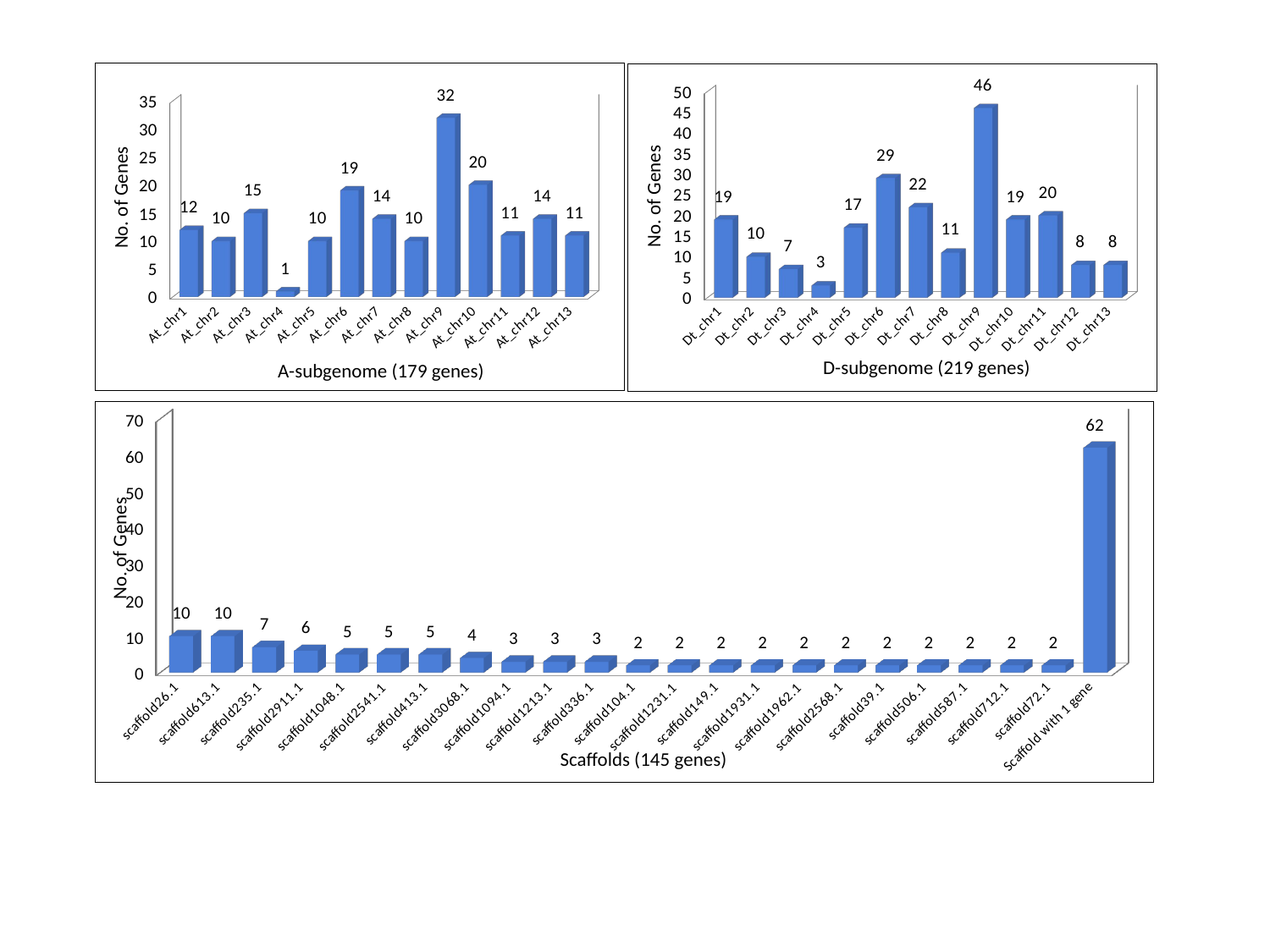

[unsupported chart]
[unsupported chart]
No. of Genes
No. of Genes
D-subgenome (219 genes)
A-subgenome (179 genes)
[unsupported chart]
No. of Genes
Scaffolds (145 genes)

## Slide 2
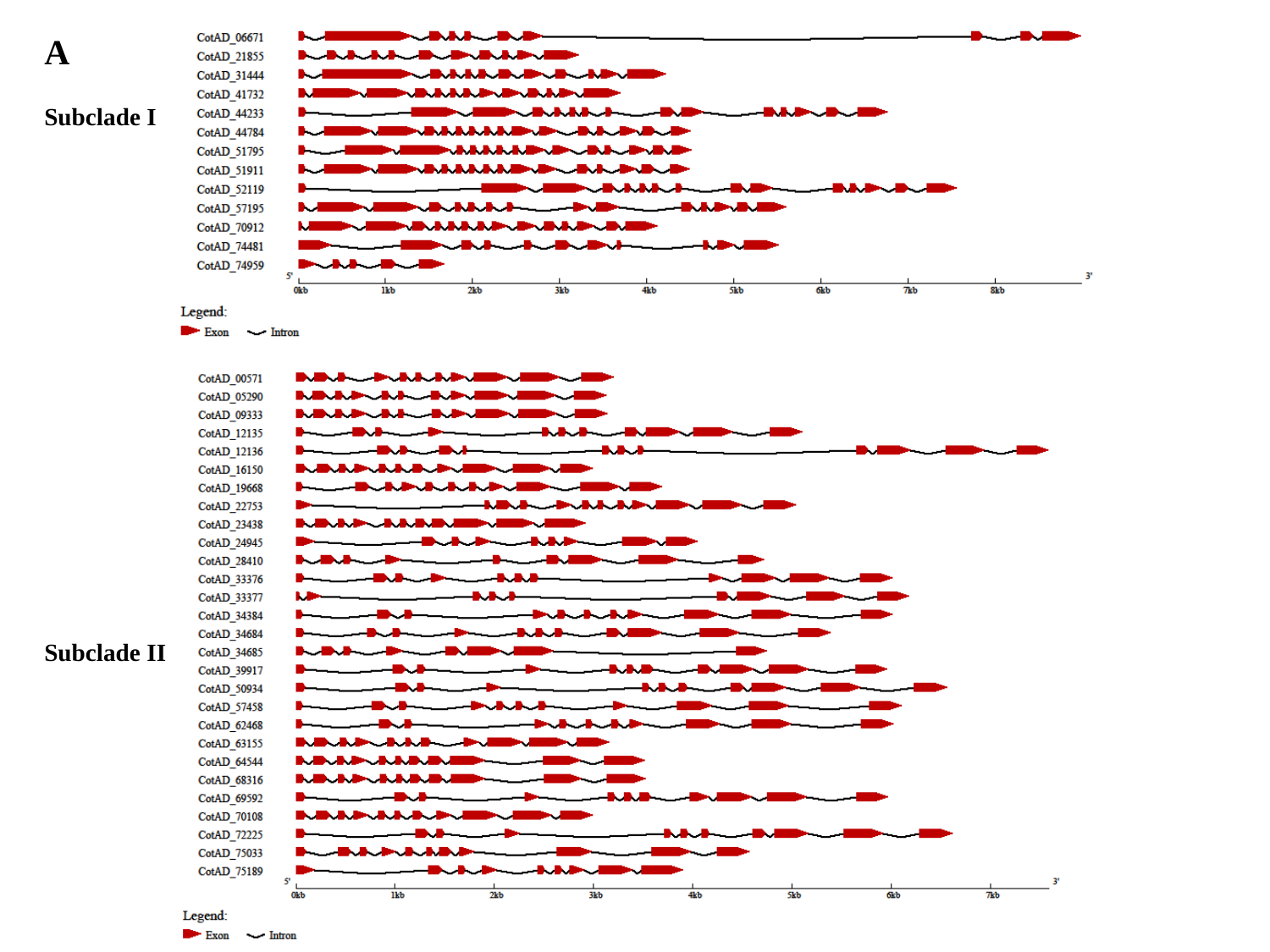

A
Subclade I
Subclade II

## Slide 3
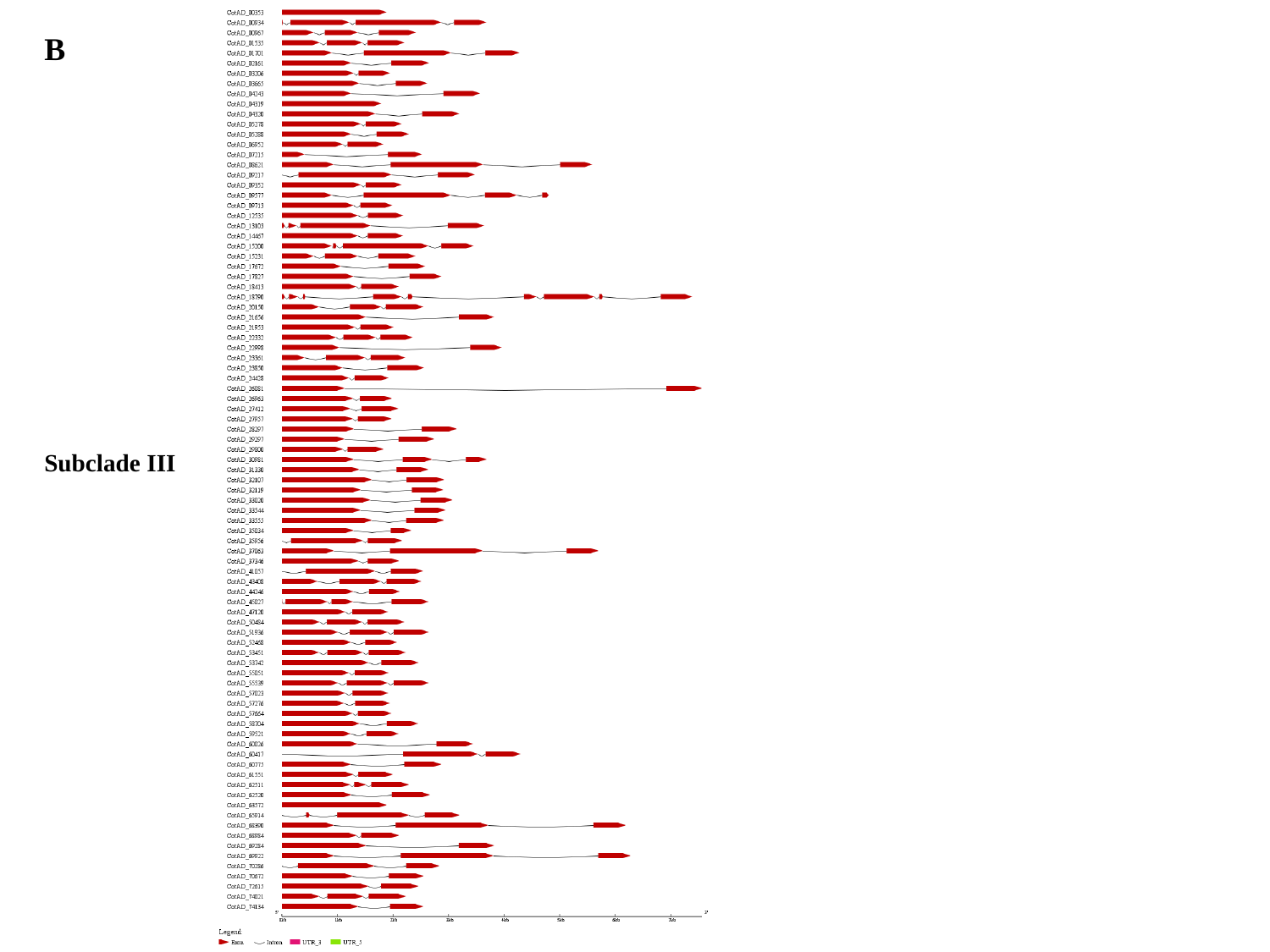

B
Subclade III

## Slide 4
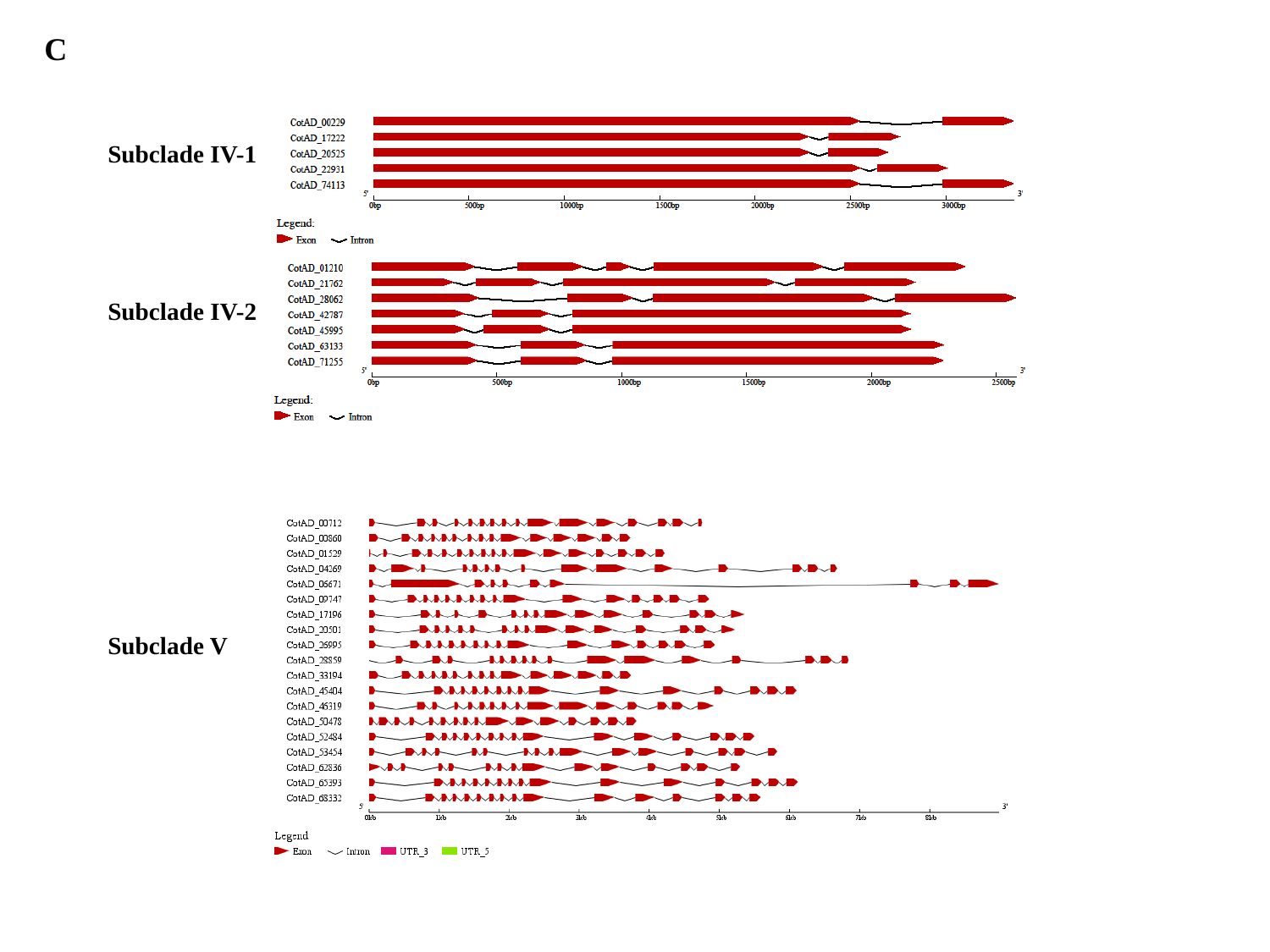

C
Subclade IV-1
Subclade IV-2
Subclade V

## Slide 5
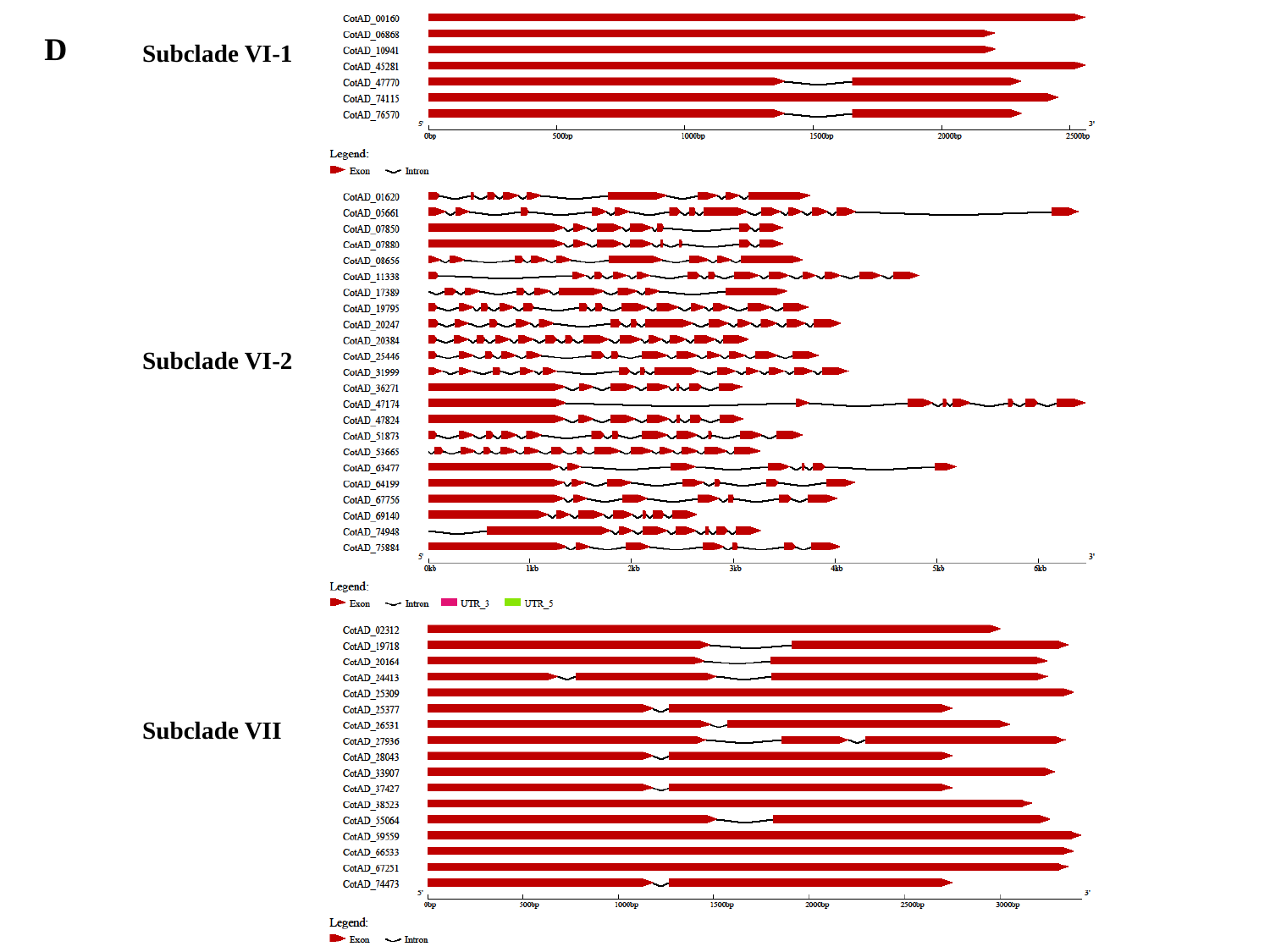

D
Subclade VI-1
Subclade VI-2
Subclade VII

## Slide 6
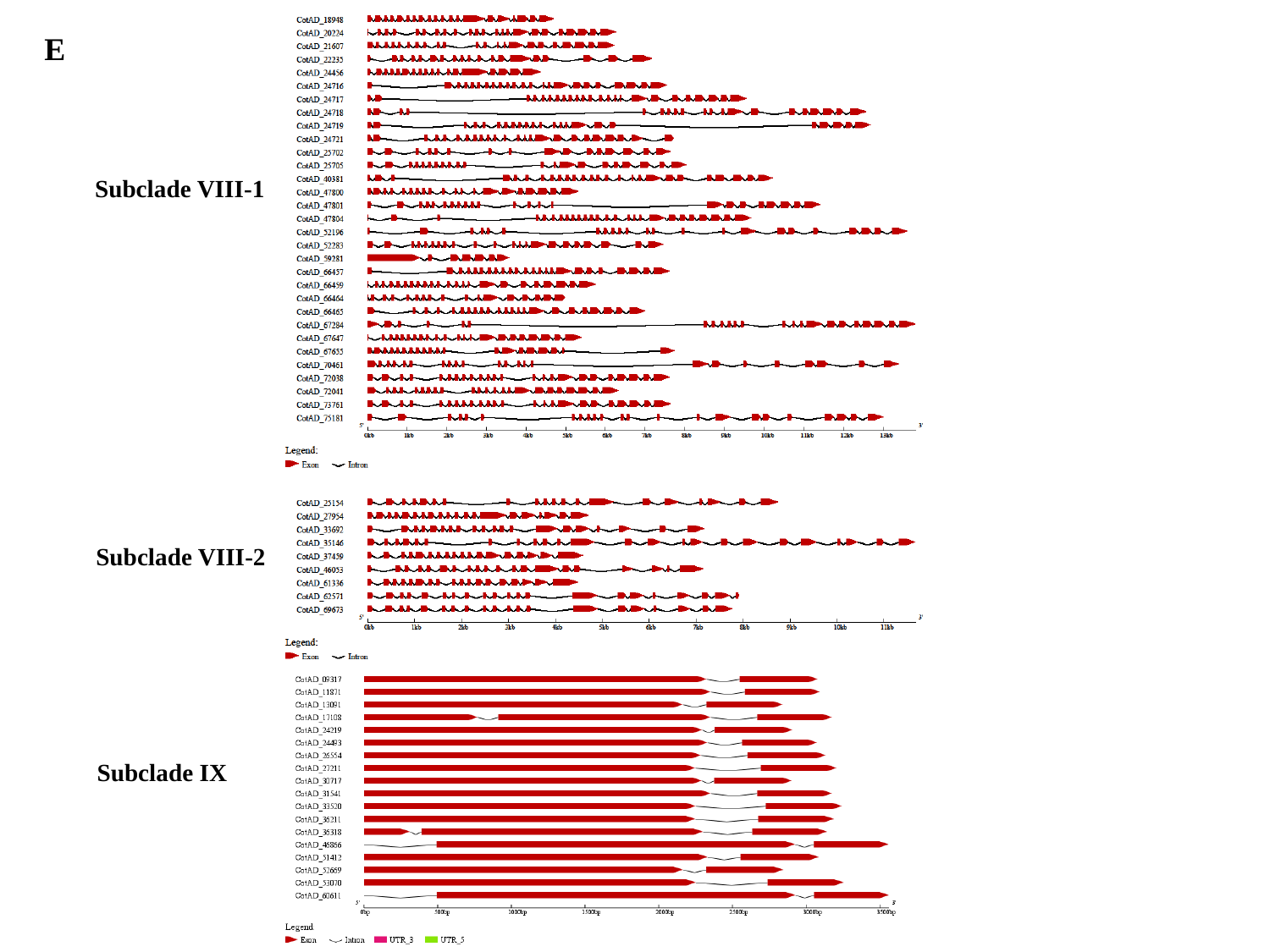

E
Subclade VIII-1
Subclade VIII-2
Subclade IX

## Slide 7
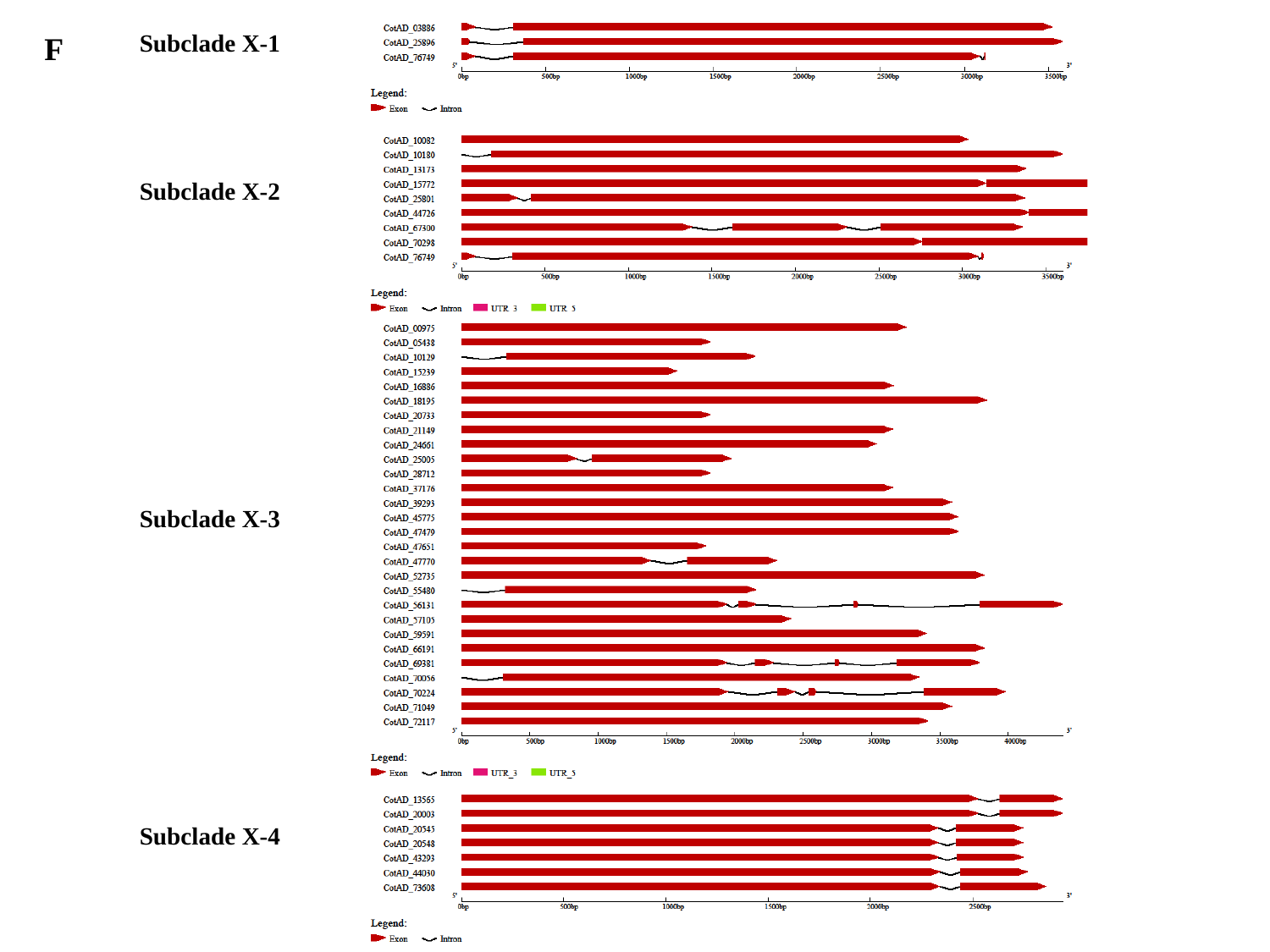

Subclade X-1
F
Subclade X-2
Subclade X-3
Subclade X-4

## Slide 8
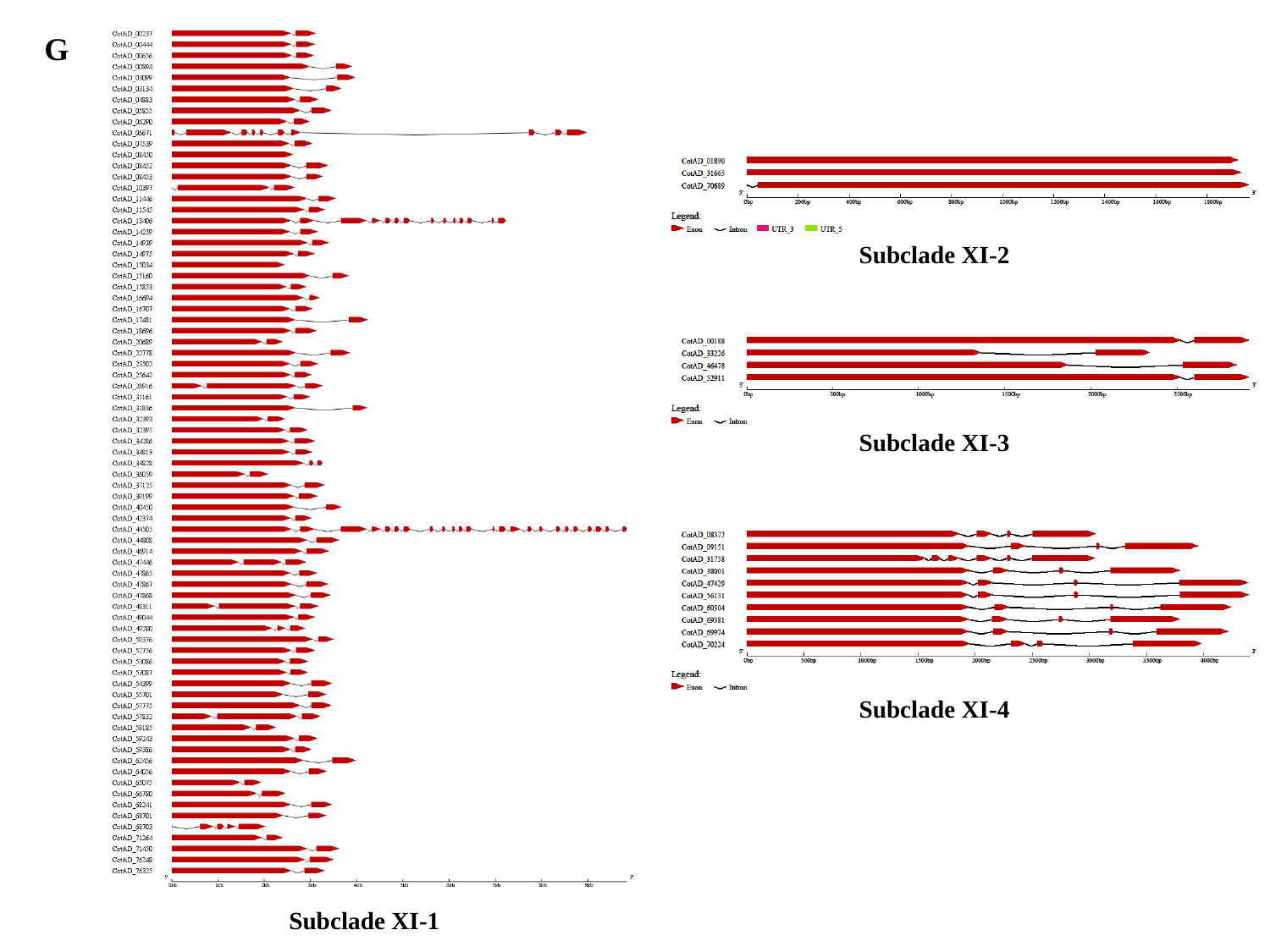

G
Subclade XI-2
Subclade XI-3
Subclade XI-4
Subclade XI-1

## Slide 9
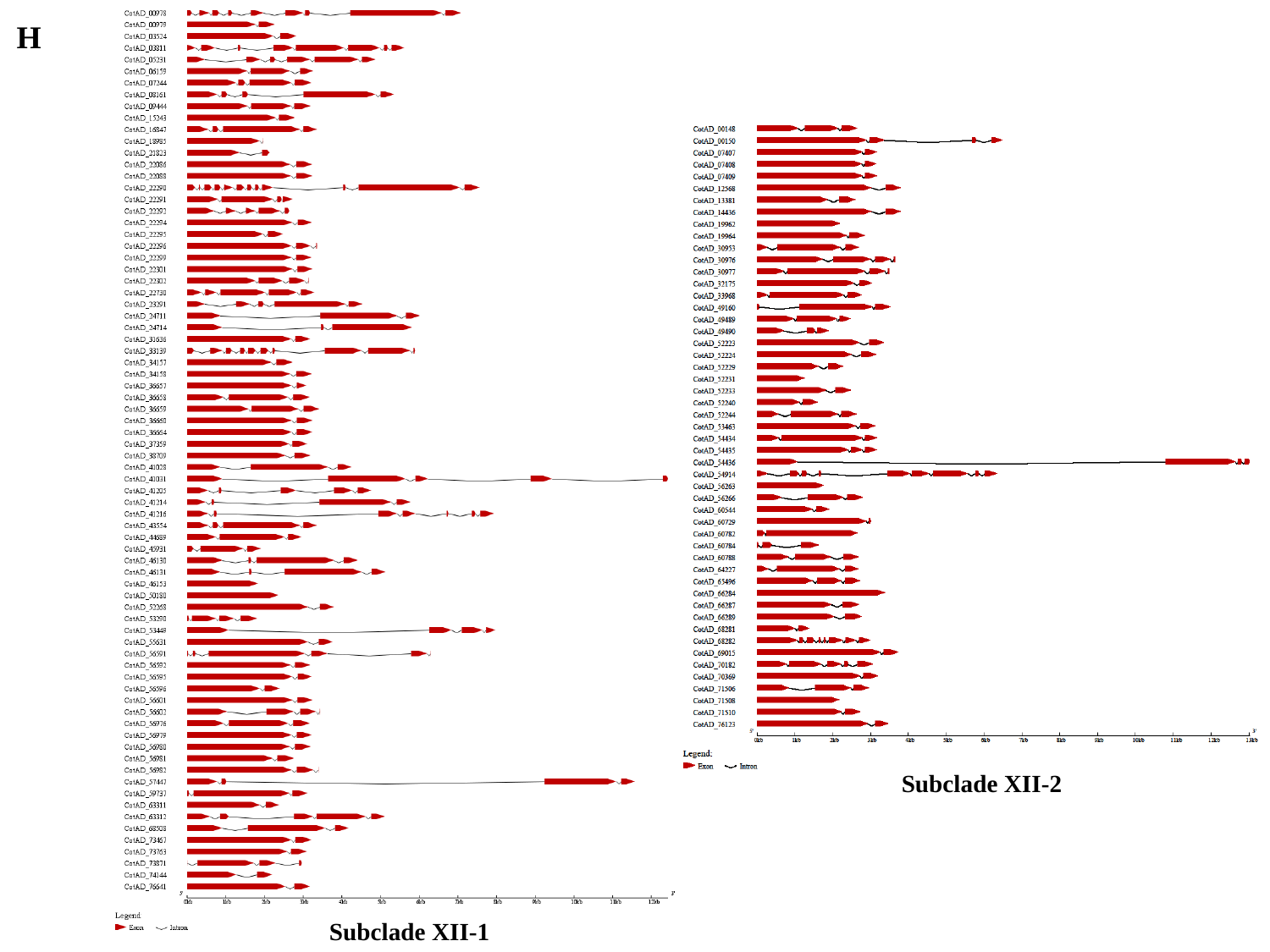

H
Subclade XII-2
Subclade XII-1

## Slide 10
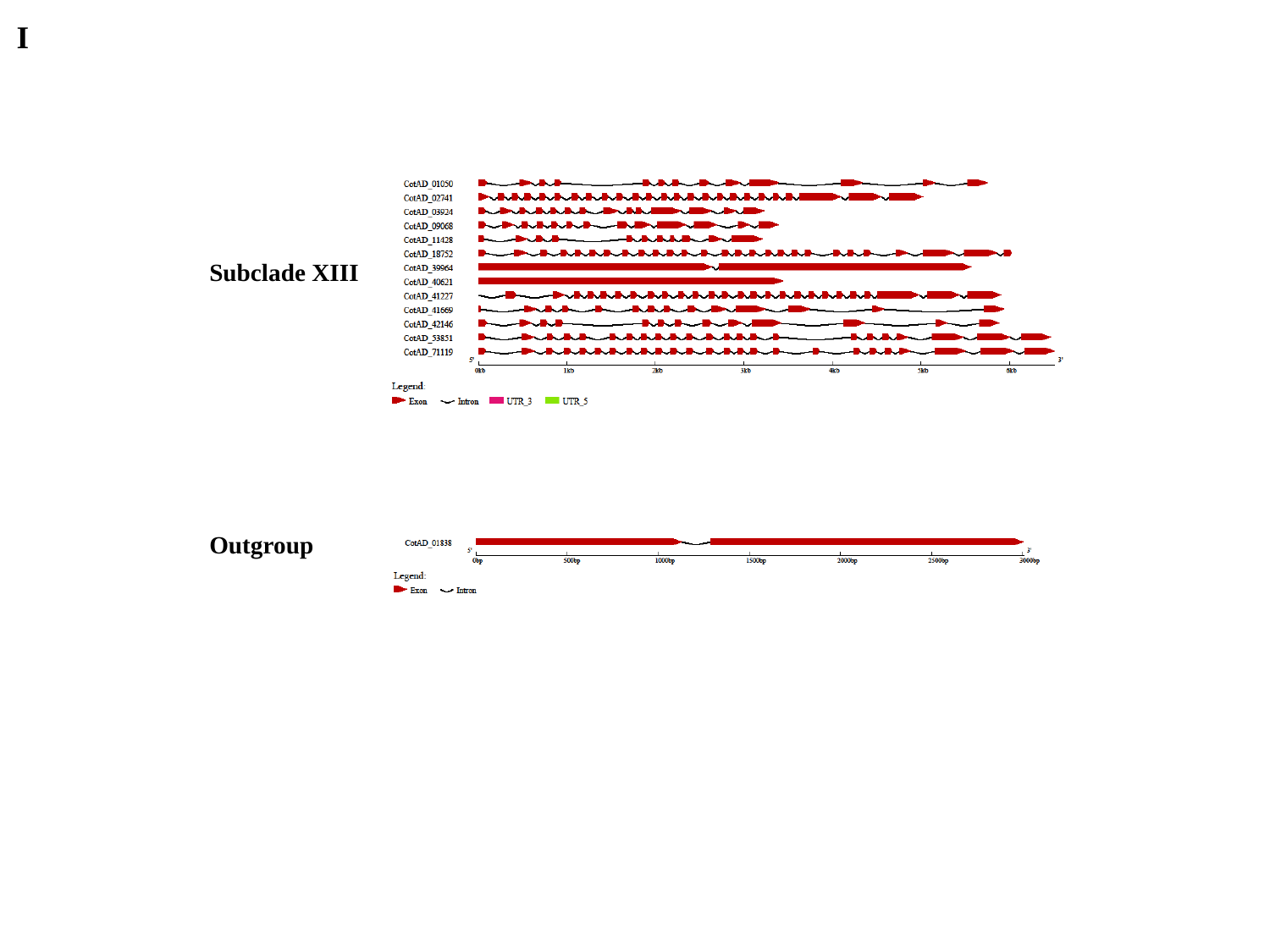

I
Subclade XIII
Outgroup

## Slide 11
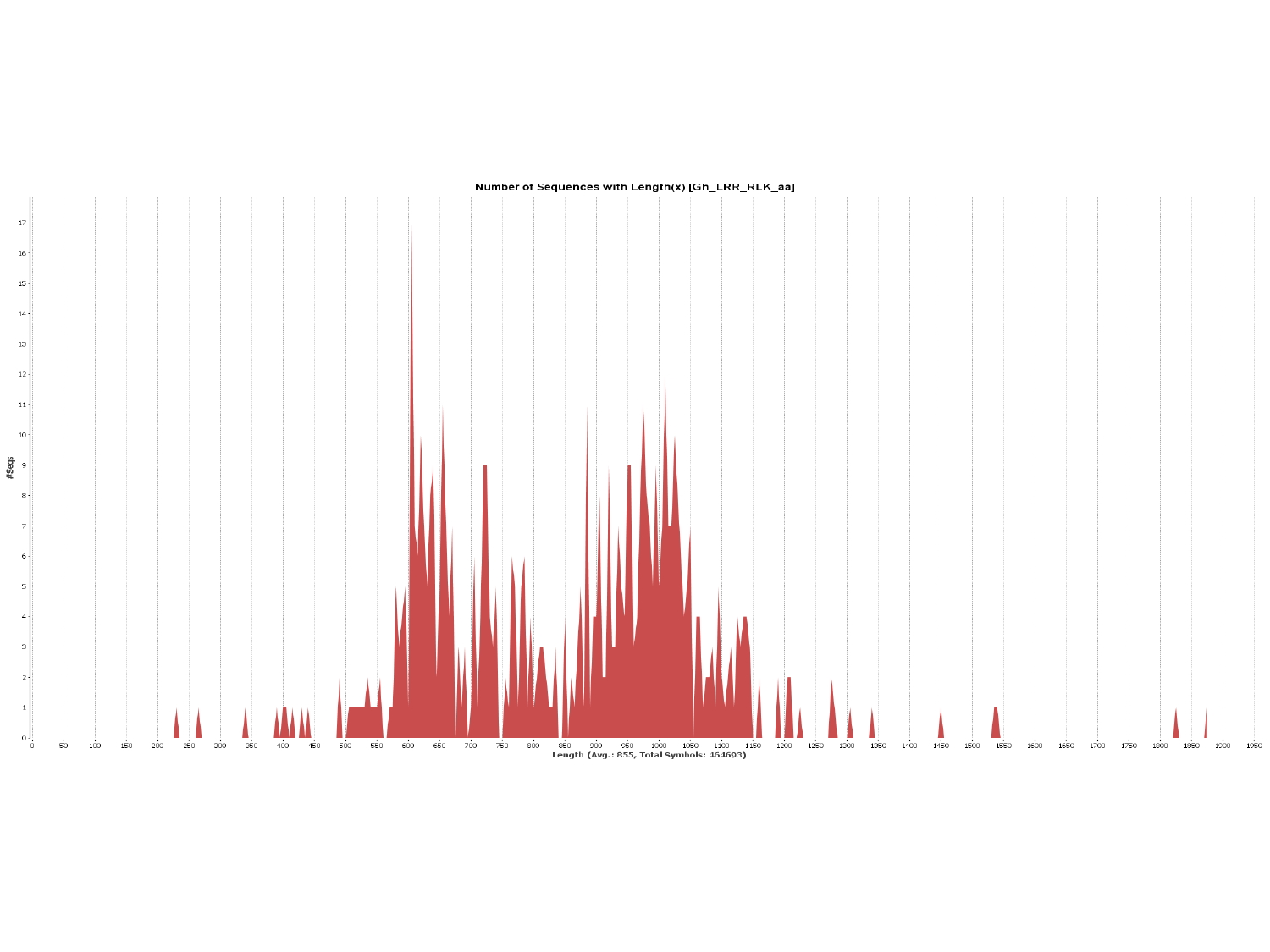

## Slide 12
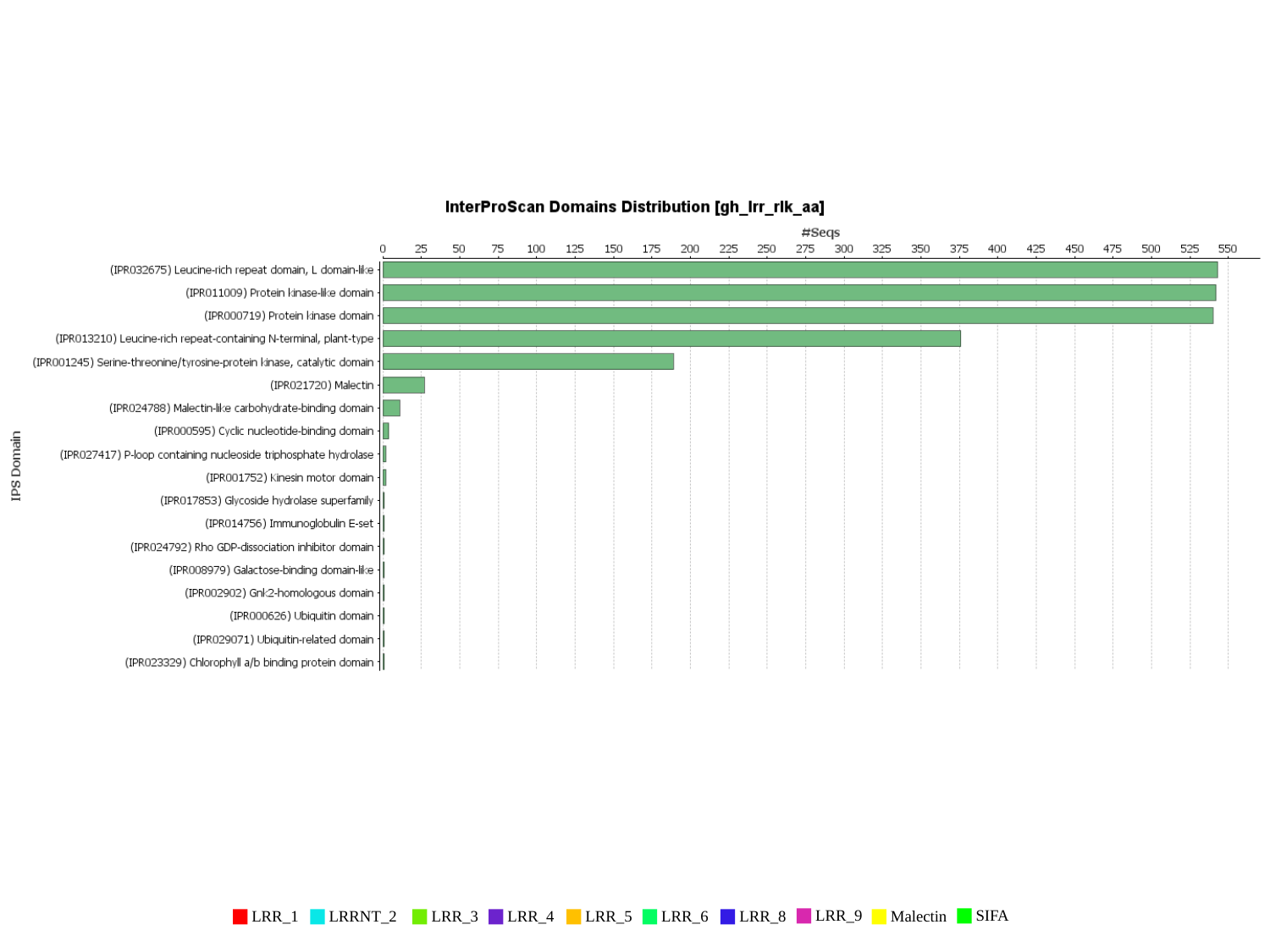

LRR_9
SIFA
LRR_1
LRRNT_2
LRR_3
LRR_4
LRR_5
LRR_6
LRR_8
Malectin

## Slide 13
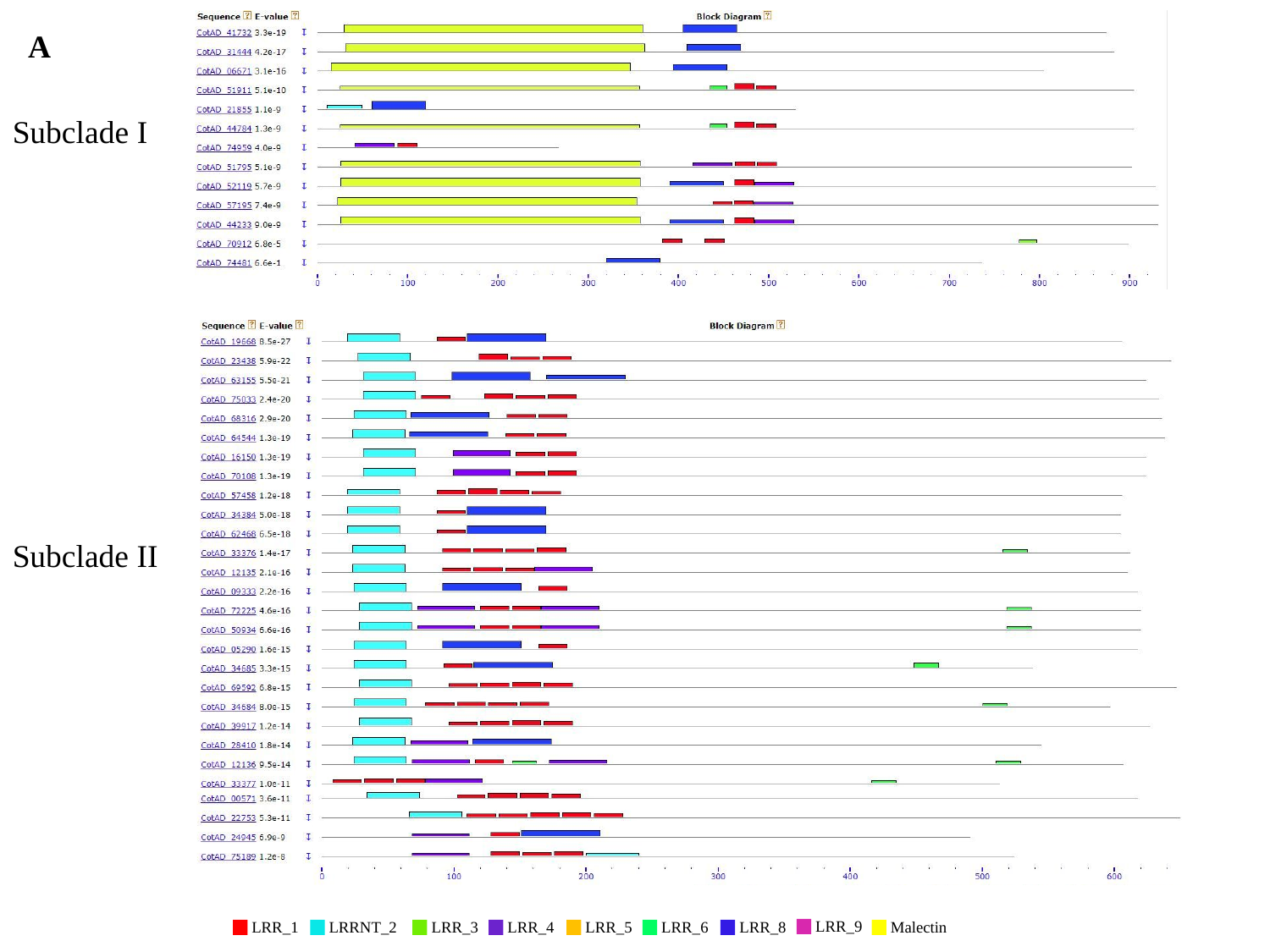

A
Subclade I
Subclade II
LRR_9
LRR_1
LRRNT_2
LRR_3
LRR_4
LRR_5
LRR_6
LRR_8
Malectin

## Slide 14
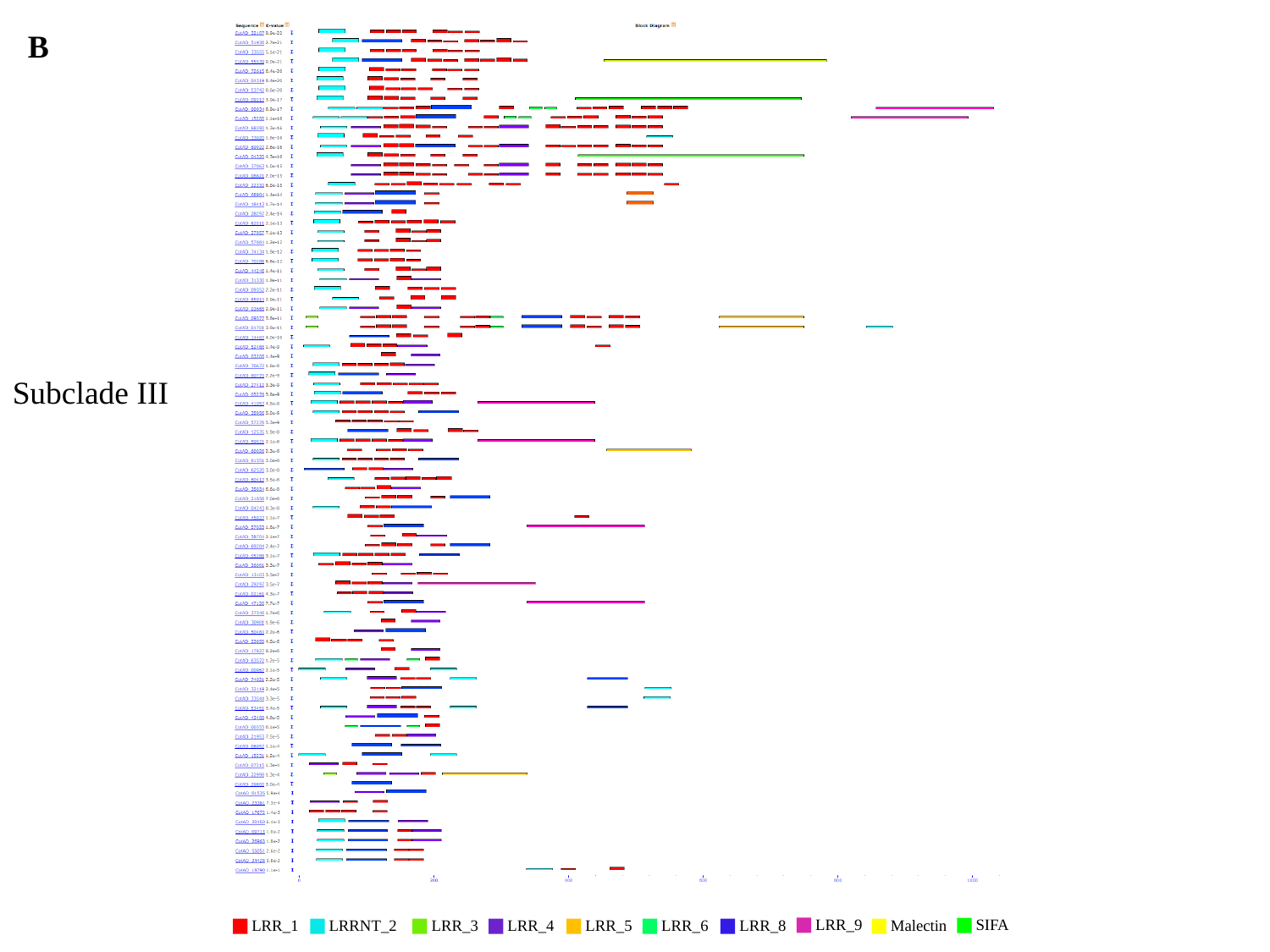

B
Subclade III
LRR_9
SIFA
LRR_1
LRRNT_2
LRR_3
LRR_4
LRR_5
LRR_6
LRR_8
Malectin

## Slide 15
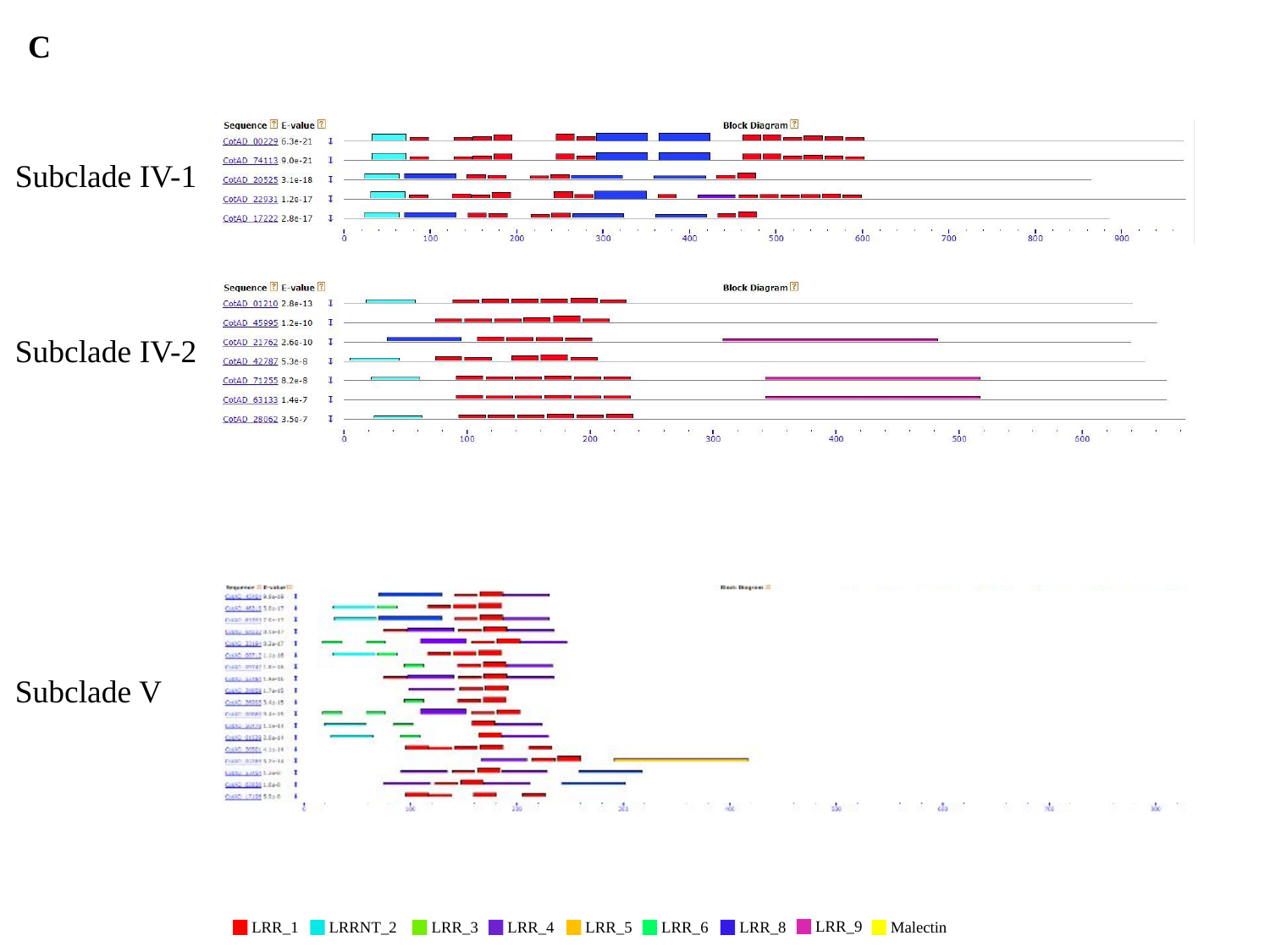

C
Subclade IV-1
Subclade IV-2
Subclade V
LRR_9
LRR_1
LRRNT_2
LRR_3
LRR_4
LRR_5
LRR_6
LRR_8
Malectin

## Slide 16
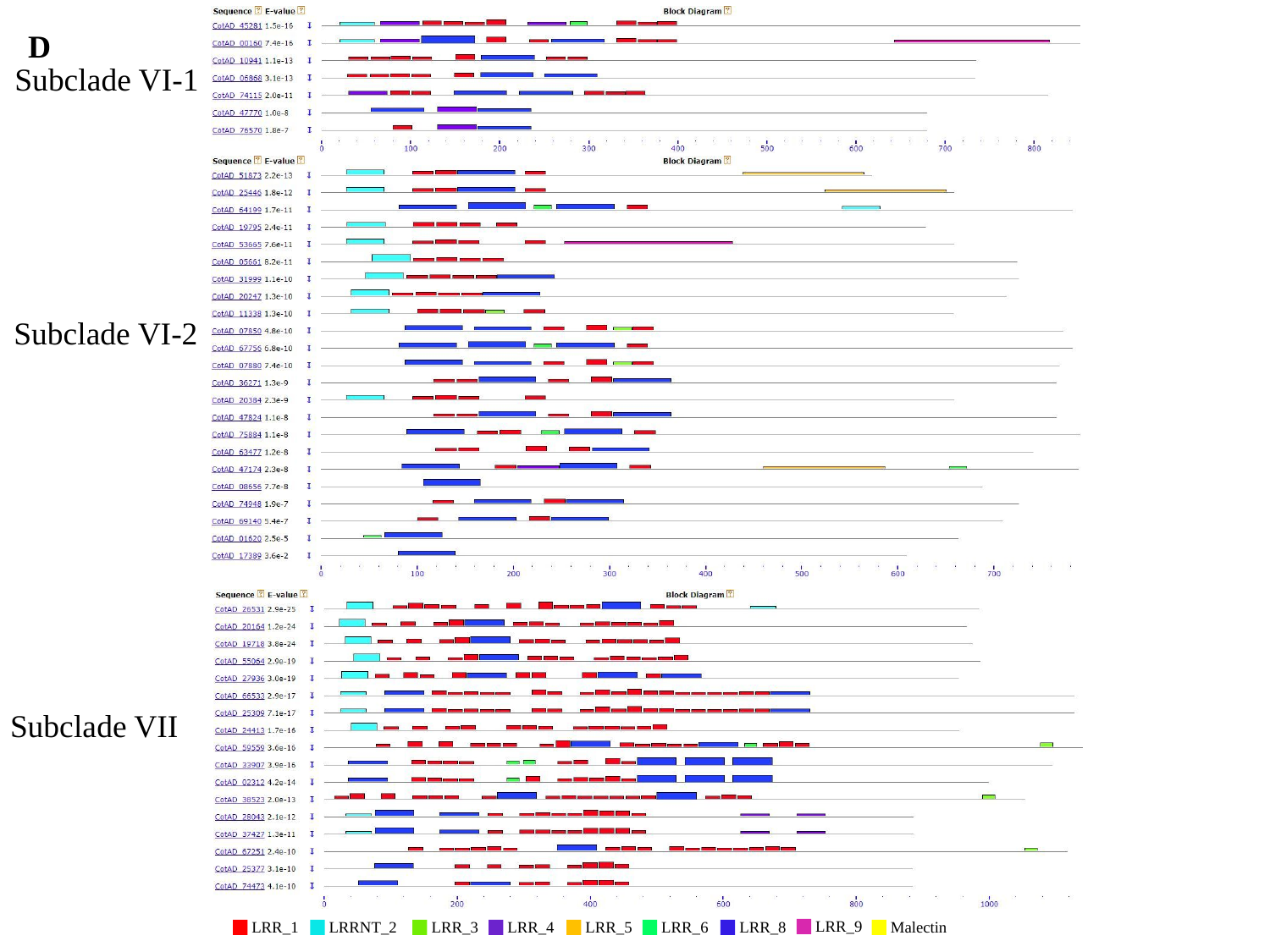

D
Subclade VI-1
Subclade VI-2
Subclade VII
LRR_9
LRR_1
LRRNT_2
LRR_3
LRR_4
LRR_5
LRR_6
LRR_8
Malectin

## Slide 17
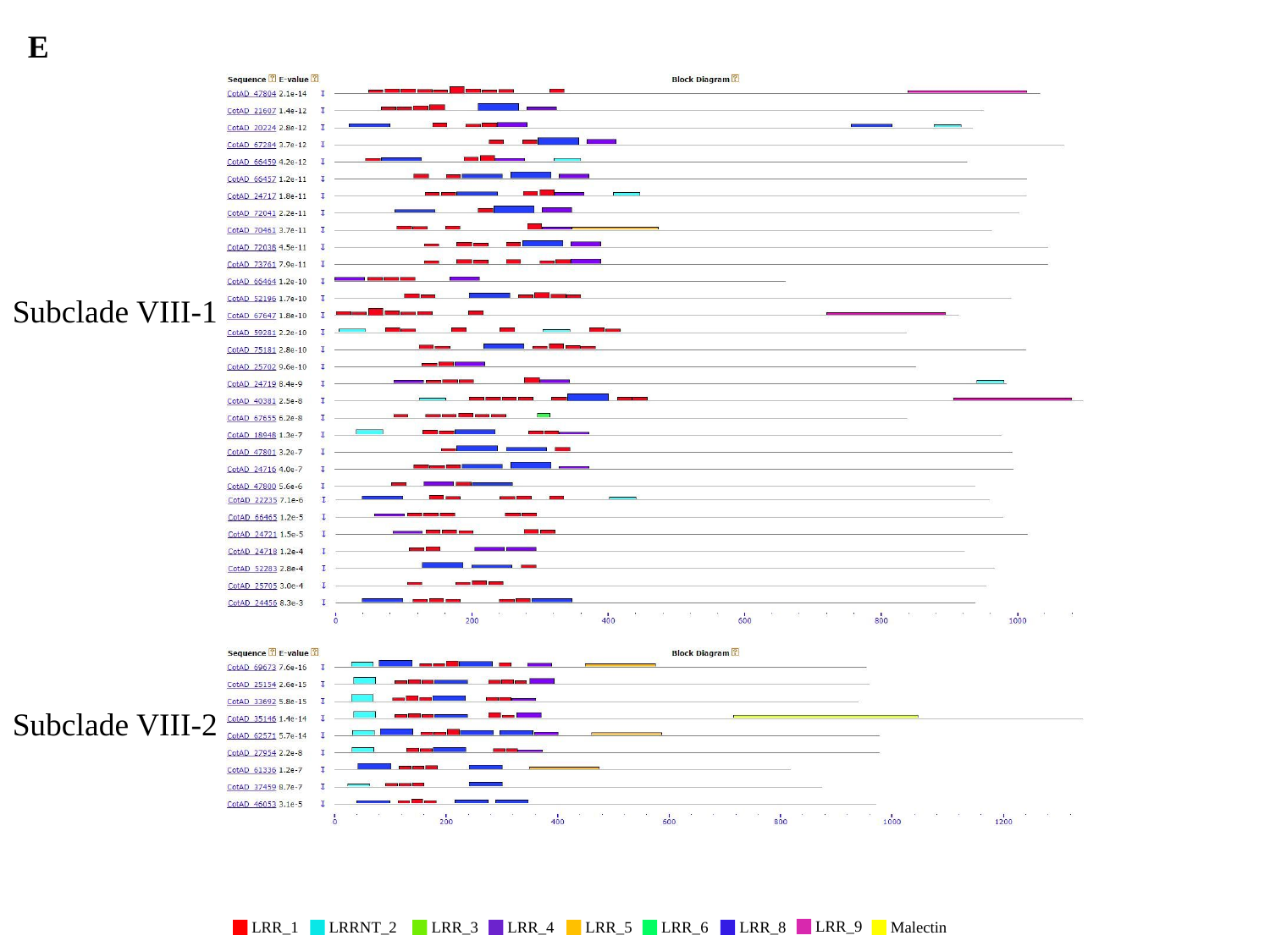

E
Subclade VIII-1
Subclade VIII-2
LRR_9
LRR_1
LRRNT_2
LRR_3
LRR_4
LRR_5
LRR_6
LRR_8
Malectin

## Slide 18
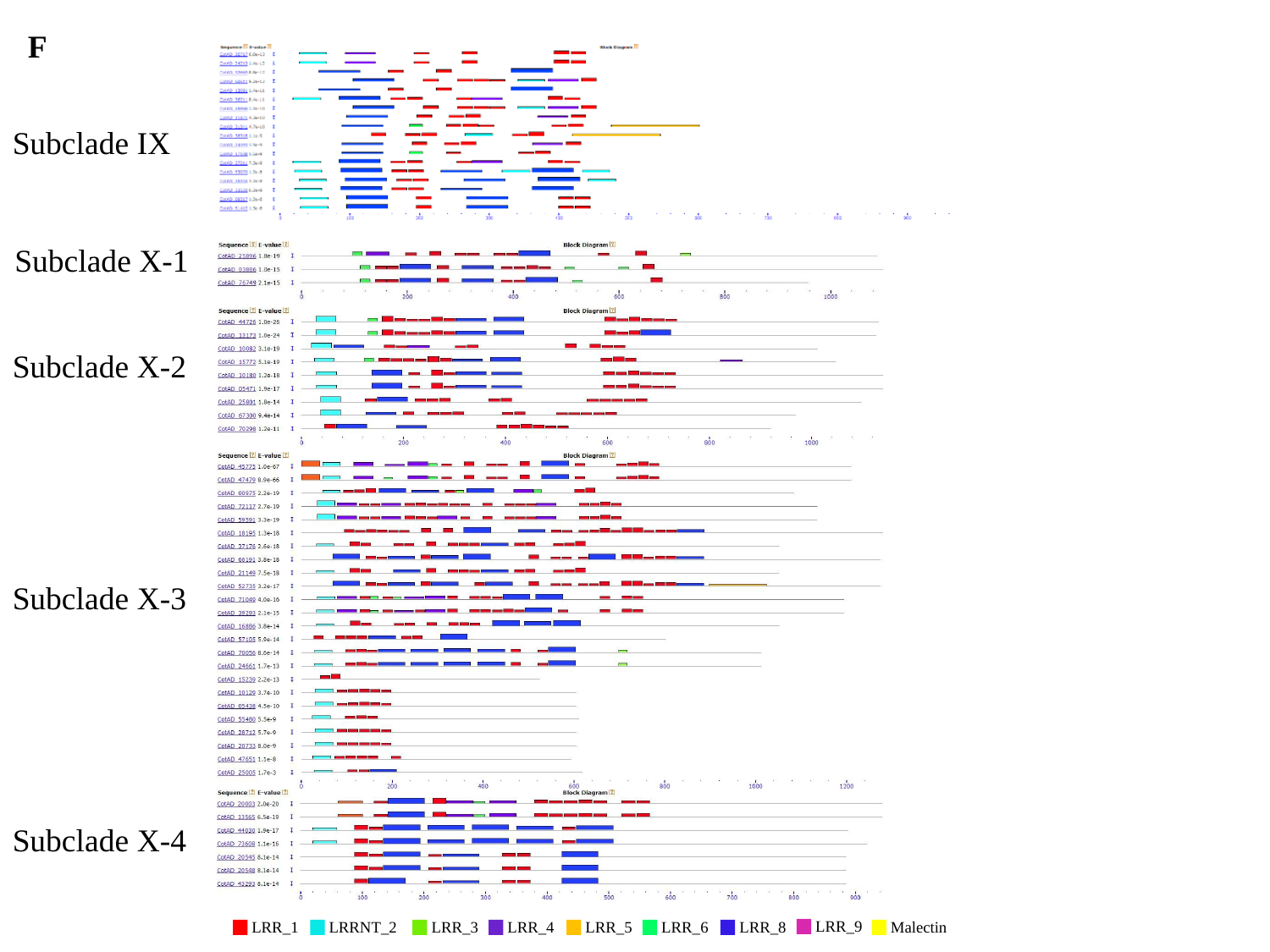

F
Subclade IX
Subclade X-1
Subclade X-2
Subclade X-3
Subclade X-4
LRR_9
LRR_1
LRRNT_2
LRR_3
LRR_4
LRR_5
LRR_6
LRR_8
Malectin

## Slide 19
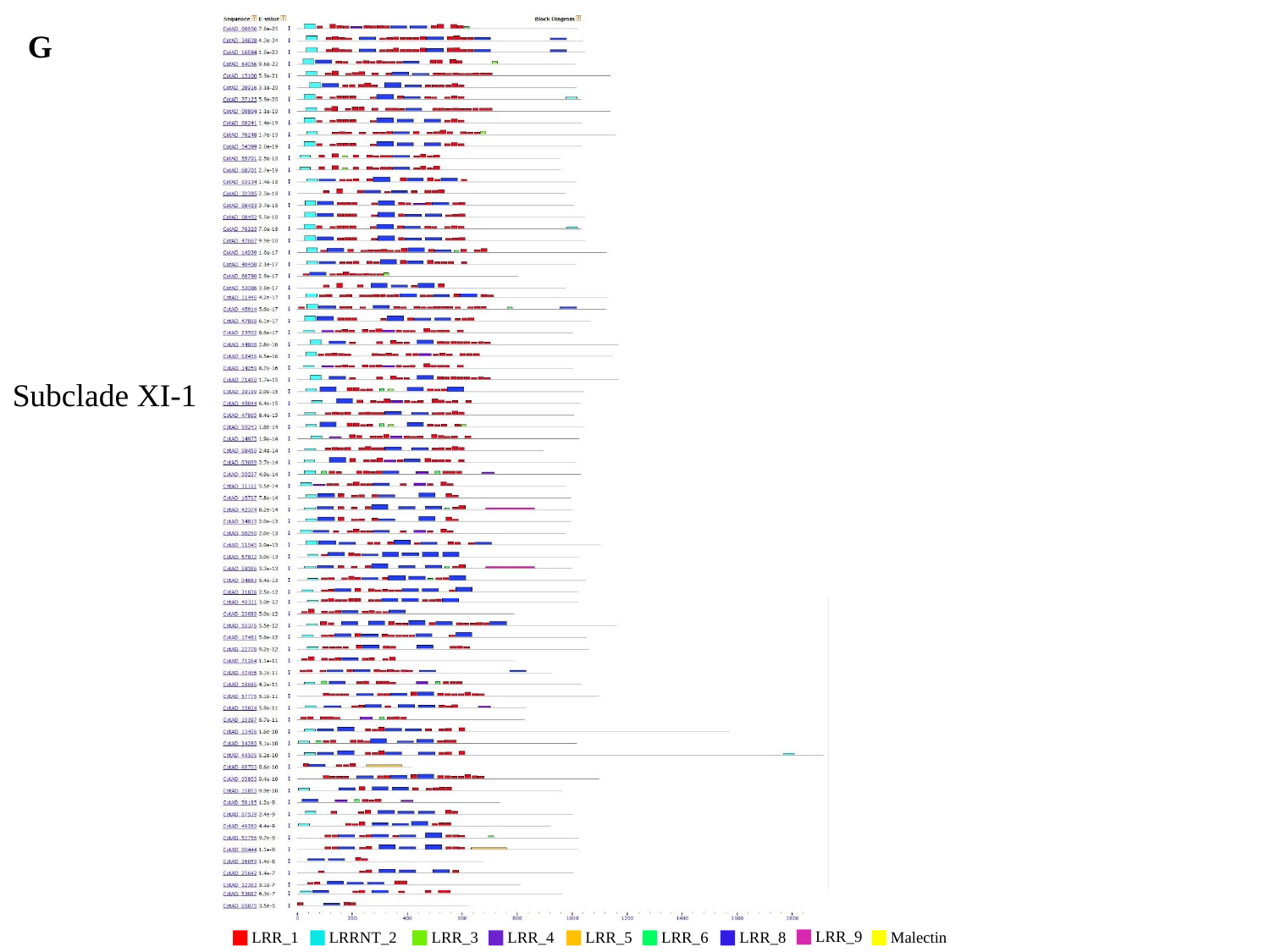

G
Subclade XI-1
LRR_9
LRR_1
LRRNT_2
LRR_3
LRR_4
LRR_5
LRR_6
LRR_8
Malectin

## Slide 20
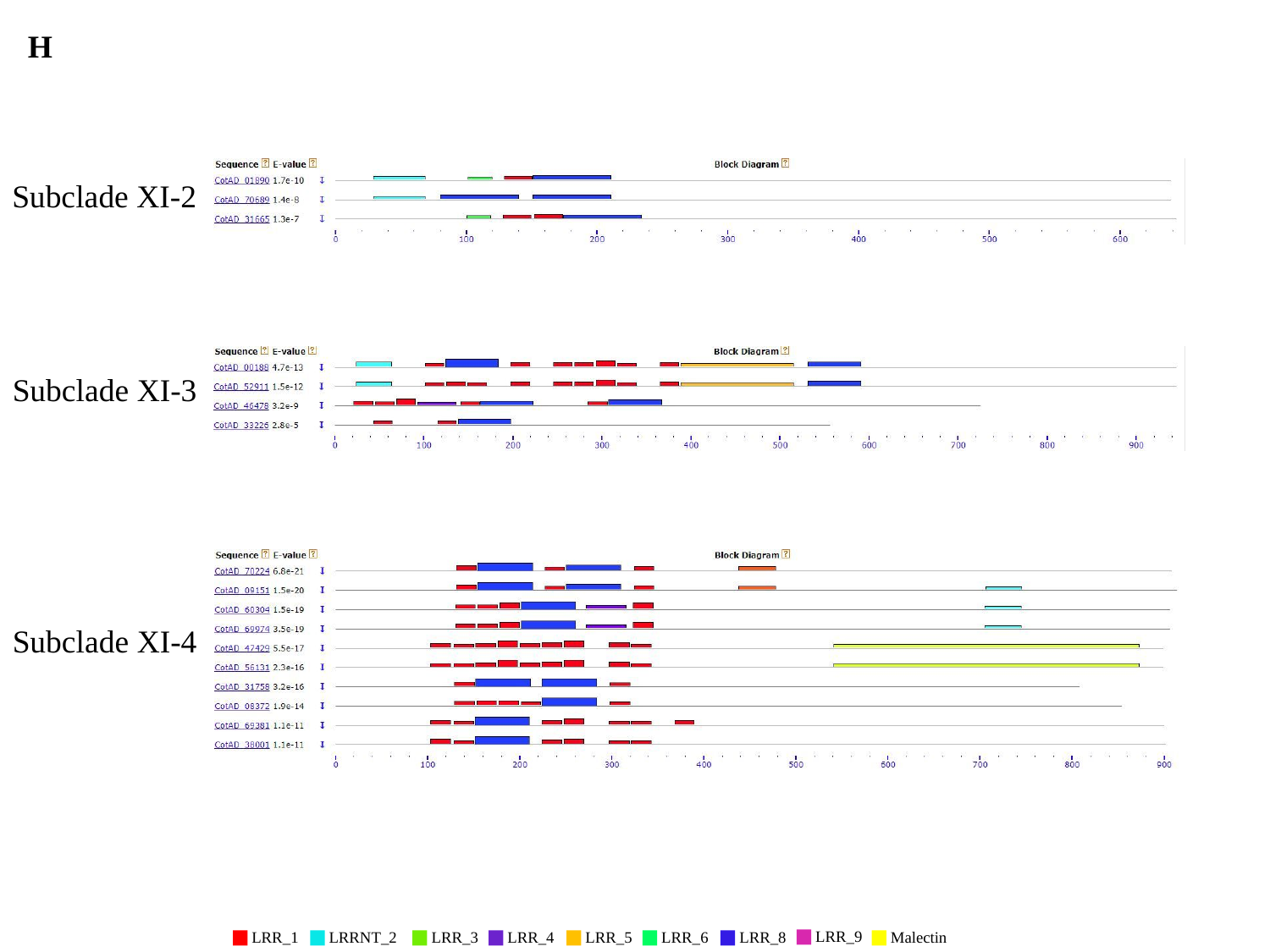

H
Subclade XI-2
Subclade XI-3
Subclade XI-4
LRR_9
LRR_1
LRRNT_2
LRR_3
LRR_4
LRR_5
LRR_6
LRR_8
Malectin

## Slide 21
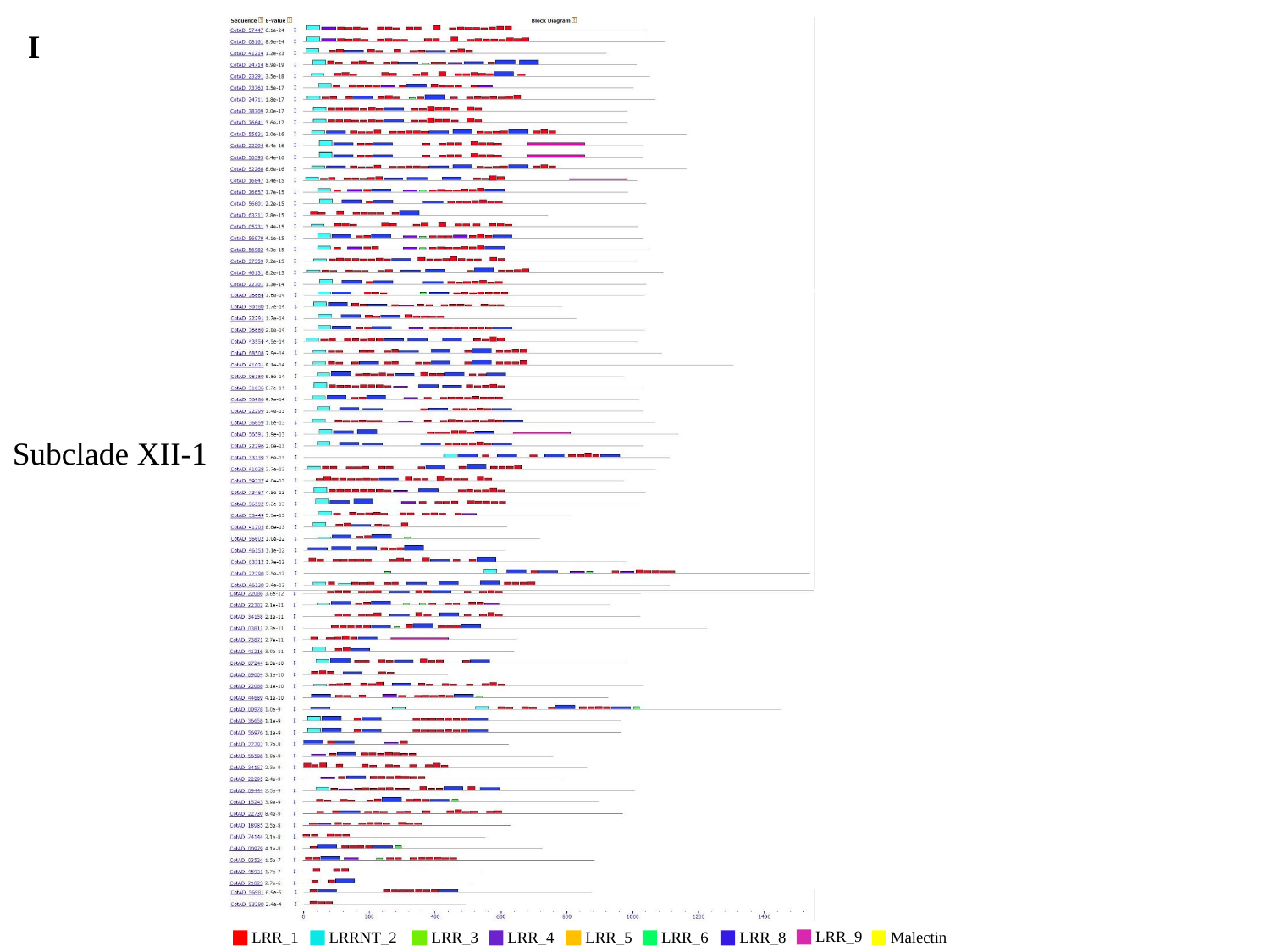

I
Subclade XII-1
LRR_9
LRR_1
LRRNT_2
LRR_3
LRR_4
LRR_5
LRR_6
LRR_8
Malectin

## Slide 22
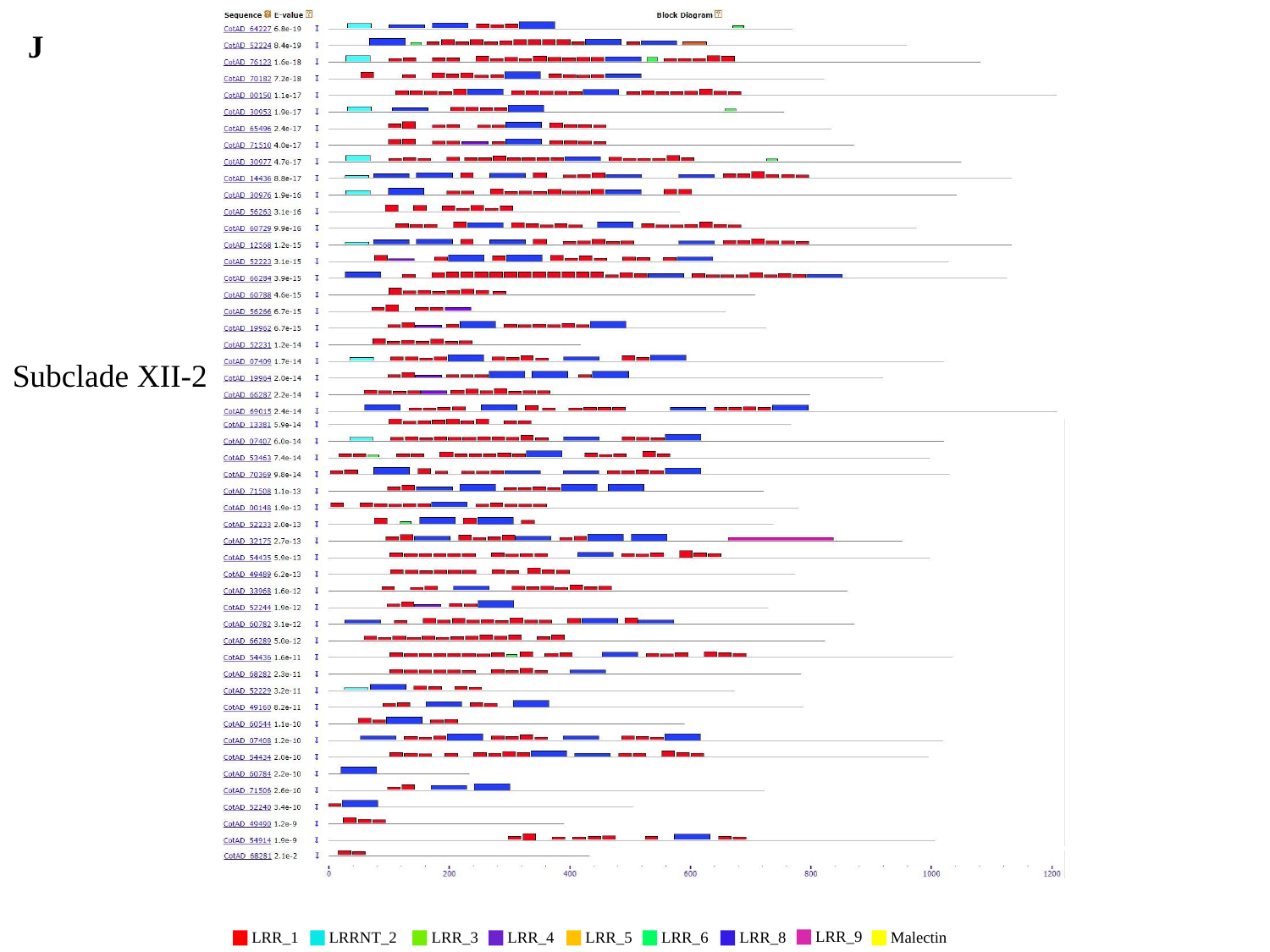

J
Subclade XII-2
LRR_9
LRR_1
LRRNT_2
LRR_3
LRR_4
LRR_5
LRR_6
LRR_8
Malectin

## Slide 23
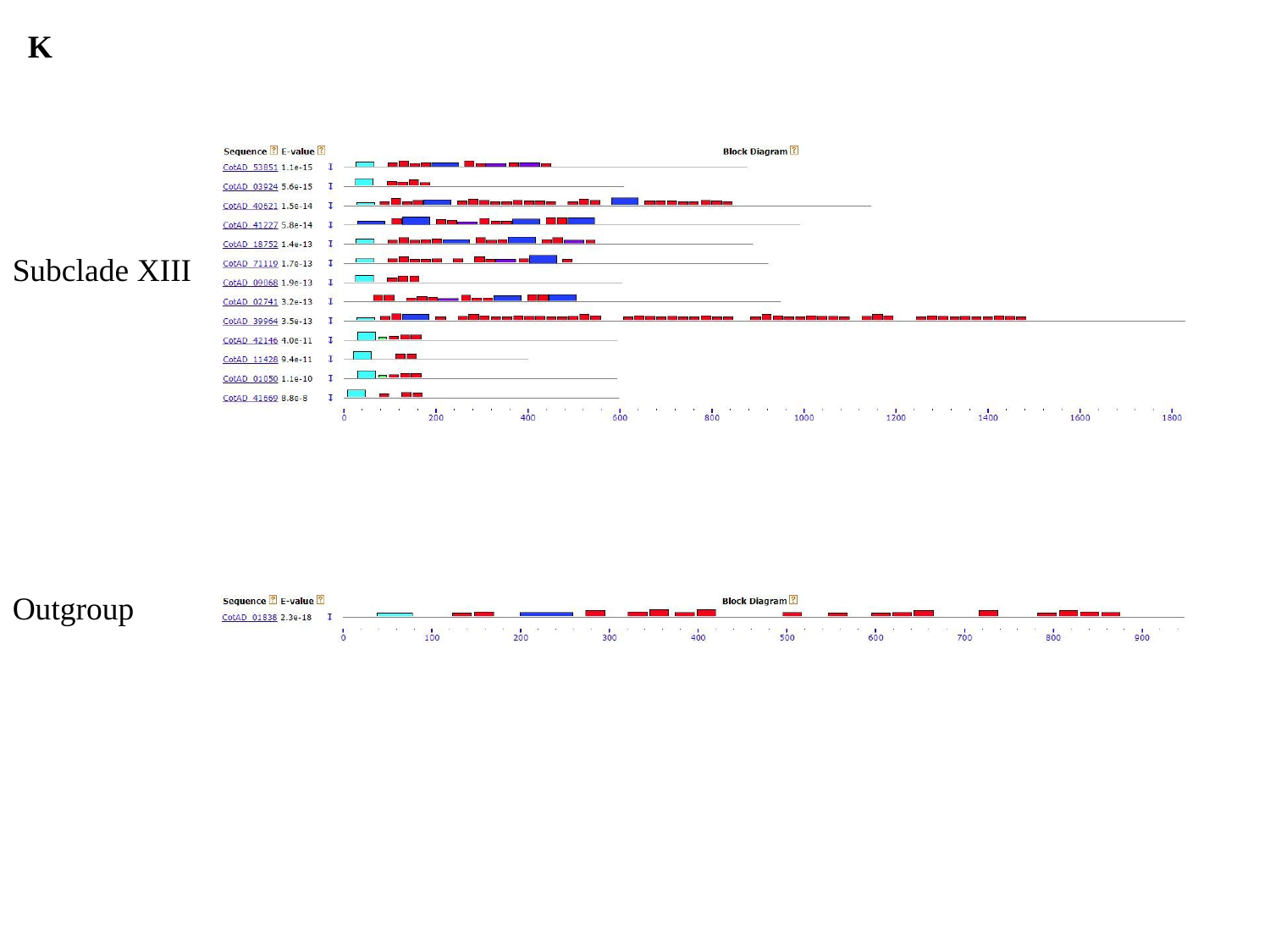

K
Subclade XIII
Outgroup

## Slide 24
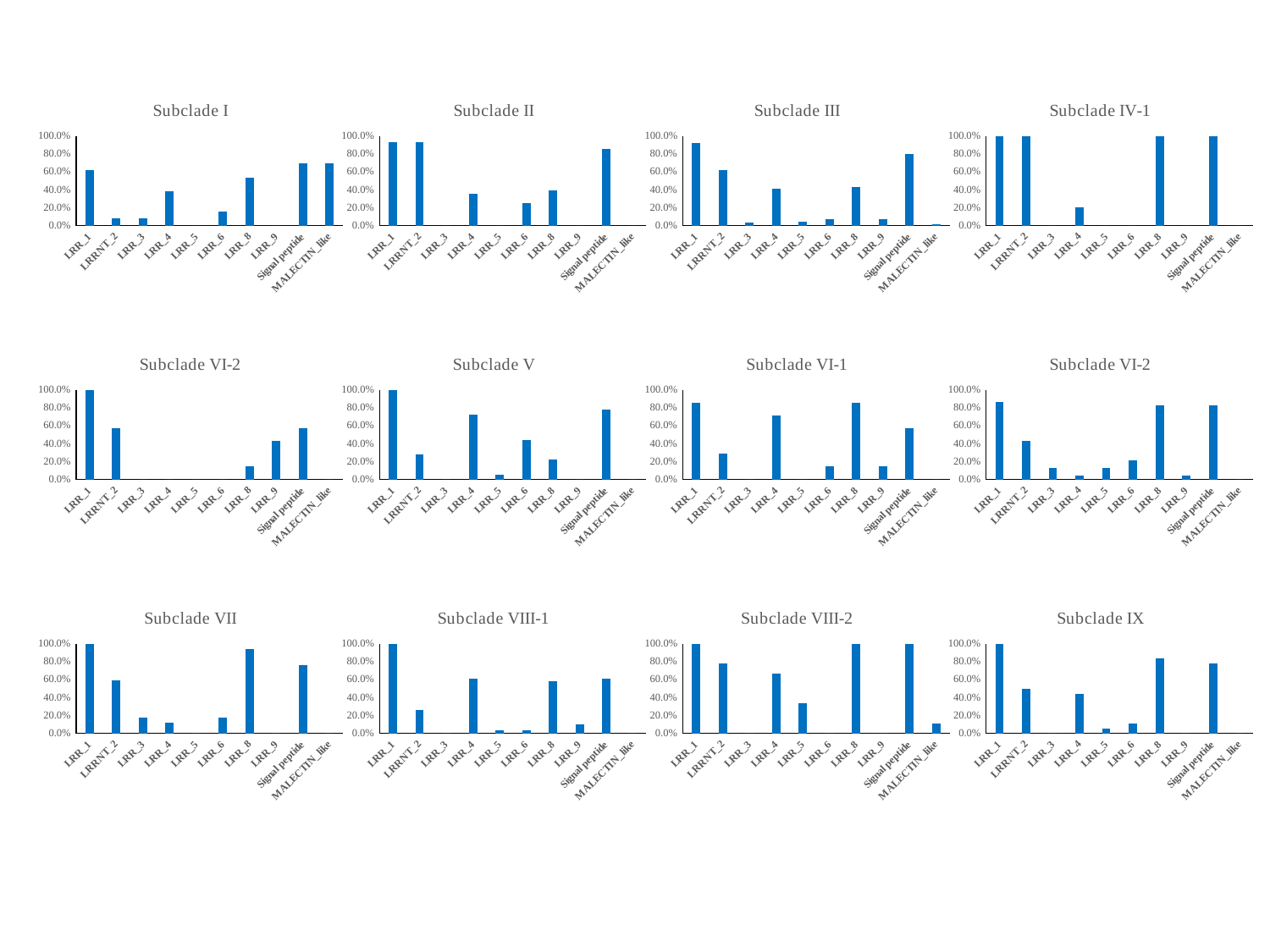

### Chart: Subclade I
| Category | |
|---|---|
| LRR_1 | 0.6153846153846154 |
| LRRNT_2 | 0.07692307692307693 |
| LRR_3 | 0.07692307692307693 |
| LRR_4 | 0.38461538461538464 |
| LRR_5 | 0.0 |
| LRR_6 | 0.15384615384615385 |
| LRR_8 | 0.5384615384615384 |
| LRR_9 | 0.0 |
| Signal peptide | 0.6923076923076923 |
| MALECTIN_like | 0.6923076923076923 |
### Chart: Subclade II
| Category | |
|---|---|
| LRR_1 | 0.9285714285714286 |
| LRRNT_2 | 0.9285714285714286 |
| LRR_3 | 0.0 |
| LRR_4 | 0.35714285714285715 |
| LRR_5 | 0.0 |
| LRR_6 | 0.25 |
| LRR_8 | 0.39285714285714285 |
| LRR_9 | 0.0 |
| Signal peptide | 0.8571428571428571 |
| MALECTIN_like | 0.0 |
### Chart: Subclade III
| Category | |
|---|---|
| LRR_1 | 0.9213483146067416 |
| LRRNT_2 | 0.6179775280898876 |
| LRR_3 | 0.033707865168539325 |
| LRR_4 | 0.4157303370786517 |
| LRR_5 | 0.0449438202247191 |
| LRR_6 | 0.06741573033707865 |
| LRR_8 | 0.42696629213483145 |
| LRR_9 | 0.06741573033707865 |
| Signal peptide | 0.797752808988764 |
| MALECTIN_like | 0.011235955056179775 |
### Chart: Subclade IV-1
| Category | |
|---|---|
| LRR_1 | 1.0 |
| LRRNT_2 | 1.0 |
| LRR_3 | 0.0 |
| LRR_4 | 0.2 |
| LRR_5 | 0.0 |
| LRR_6 | 0.0 |
| LRR_8 | 1.0 |
| LRR_9 | 0.0 |
| Signal peptide | 1.0 |
| MALECTIN_like | 0.0 |
### Chart: Subclade VI-2
| Category | |
|---|---|
| LRR_1 | 1.0 |
| LRRNT_2 | 0.5714285714285714 |
| LRR_3 | 0.0 |
| LRR_4 | 0.0 |
| LRR_5 | 0.0 |
| LRR_6 | 0.0 |
| LRR_8 | 0.14285714285714285 |
| LRR_9 | 0.42857142857142855 |
| Signal peptide | 0.5714285714285714 |
| MALECTIN_like | 0.0 |
### Chart: Subclade V
| Category | |
|---|---|
| LRR_1 | 1.0 |
| LRRNT_2 | 0.2777777777777778 |
| LRR_3 | 0.0 |
| LRR_4 | 0.7222222222222222 |
| LRR_5 | 0.05555555555555555 |
| LRR_6 | 0.4444444444444444 |
| LRR_8 | 0.2222222222222222 |
| LRR_9 | 0.0 |
| Signal peptide | 0.7777777777777778 |
| MALECTIN_like | 0.0 |
### Chart: Subclade VI-1
| Category | |
|---|---|
| LRR_1 | 0.8571428571428571 |
| LRRNT_2 | 0.2857142857142857 |
| LRR_3 | 0.0 |
| LRR_4 | 0.7142857142857143 |
| LRR_5 | 0.0 |
| LRR_6 | 0.14285714285714285 |
| LRR_8 | 0.8571428571428571 |
| LRR_9 | 0.14285714285714285 |
| Signal peptide | 0.5714285714285714 |
| MALECTIN_like | 0.0 |
### Chart: Subclade VI-2
| Category | |
|---|---|
| LRR_1 | 0.8695652173913043 |
| LRRNT_2 | 0.43478260869565216 |
| LRR_3 | 0.13043478260869565 |
| LRR_4 | 0.043478260869565216 |
| LRR_5 | 0.13043478260869565 |
| LRR_6 | 0.21739130434782608 |
| LRR_8 | 0.8260869565217391 |
| LRR_9 | 0.043478260869565216 |
| Signal peptide | 0.8260869565217391 |
| MALECTIN_like | 0.0 |
### Chart: Subclade VII
| Category | |
|---|---|
| LRR_1 | 1.0 |
| LRRNT_2 | 0.5882352941176471 |
| LRR_3 | 0.17647058823529413 |
| LRR_4 | 0.11764705882352941 |
| LRR_5 | 0.0 |
| LRR_6 | 0.17647058823529413 |
| LRR_8 | 0.9411764705882353 |
| LRR_9 | 0.0 |
| Signal peptide | 0.7647058823529411 |
| MALECTIN_like | 0.0 |
### Chart: Subclade VIII-1
| Category | |
|---|---|
| LRR_1 | 1.0 |
| LRRNT_2 | 0.25806451612903225 |
| LRR_3 | 0.0 |
| LRR_4 | 0.6129032258064516 |
| LRR_5 | 0.03225806451612903 |
| LRR_6 | 0.03225806451612903 |
| LRR_8 | 0.5806451612903226 |
| LRR_9 | 0.0967741935483871 |
| Signal peptide | 0.6129032258064516 |
| MALECTIN_like | 0.0 |
### Chart: Subclade VIII-2
| Category | |
|---|---|
| LRR_1 | 1.0 |
| LRRNT_2 | 0.7777777777777778 |
| LRR_3 | 0.0 |
| LRR_4 | 0.6666666666666666 |
| LRR_5 | 0.3333333333333333 |
| LRR_6 | 0.0 |
| LRR_8 | 1.0 |
| LRR_9 | 0.0 |
| Signal peptide | 1.0 |
| MALECTIN_like | 0.1111111111111111 |
### Chart: Subclade IX
| Category | |
|---|---|
| LRR_1 | 1.0 |
| LRRNT_2 | 0.5 |
| LRR_3 | 0.0 |
| LRR_4 | 0.4444444444444444 |
| LRR_5 | 0.05555555555555555 |
| LRR_6 | 0.1111111111111111 |
| LRR_8 | 0.8333333333333334 |
| LRR_9 | 0.0 |
| Signal peptide | 0.7777777777777778 |
| MALECTIN_like | 0.0 |

## Slide 25
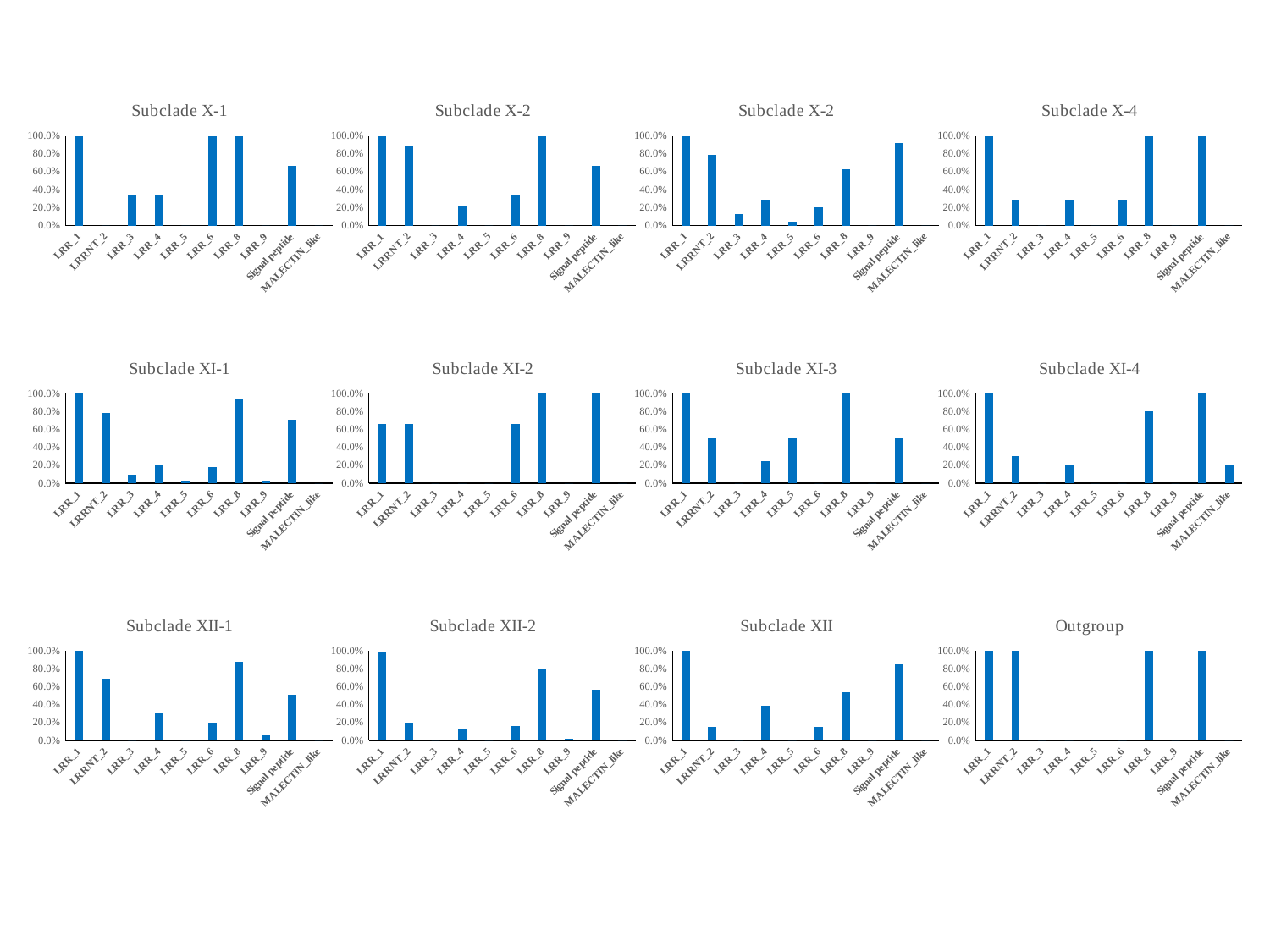

### Chart: Subclade X-1
| Category | |
|---|---|
| LRR_1 | 1.0 |
| LRRNT_2 | 0.0 |
| LRR_3 | 0.3333333333333333 |
| LRR_4 | 0.3333333333333333 |
| LRR_5 | 0.0 |
| LRR_6 | 1.0 |
| LRR_8 | 1.0 |
| LRR_9 | 0.0 |
| Signal peptide | 0.6666666666666666 |
| MALECTIN_like | 0.0 |
### Chart: Subclade X-2
| Category | |
|---|---|
| LRR_1 | 1.0 |
| LRRNT_2 | 0.8888888888888888 |
| LRR_3 | 0.0 |
| LRR_4 | 0.2222222222222222 |
| LRR_5 | 0.0 |
| LRR_6 | 0.3333333333333333 |
| LRR_8 | 1.0 |
| LRR_9 | 0.0 |
| Signal peptide | 0.6666666666666666 |
| MALECTIN_like | 0.0 |
### Chart: Subclade X-2
| Category | |
|---|---|
| LRR_1 | 1.0 |
| LRRNT_2 | 0.7916666666666666 |
| LRR_3 | 0.125 |
| LRR_4 | 0.2916666666666667 |
| LRR_5 | 0.041666666666666664 |
| LRR_6 | 0.20833333333333334 |
| LRR_8 | 0.625 |
| LRR_9 | 0.0 |
| Signal peptide | 0.9166666666666666 |
| MALECTIN_like | 0.0 |
### Chart: Subclade X-4
| Category | |
|---|---|
| LRR_1 | 1.0 |
| LRRNT_2 | 0.2857142857142857 |
| LRR_3 | 0.0 |
| LRR_4 | 0.2857142857142857 |
| LRR_5 | 0.0 |
| LRR_6 | 0.2857142857142857 |
| LRR_8 | 1.0 |
| LRR_9 | 0.0 |
| Signal peptide | 1.0 |
| MALECTIN_like | 0.0 |
### Chart: Subclade XI-1
| Category | |
|---|---|
| LRR_1 | 1.0 |
| LRRNT_2 | 0.7894736842105263 |
| LRR_3 | 0.09210526315789473 |
| LRR_4 | 0.19736842105263158 |
| LRR_5 | 0.02631578947368421 |
| LRR_6 | 0.18421052631578946 |
| LRR_8 | 0.9342105263157895 |
| LRR_9 | 0.02631578947368421 |
| Signal peptide | 0.7105263157894737 |
| MALECTIN_like | 0.0 |
### Chart: Subclade XI-2
| Category | |
|---|---|
| LRR_1 | 0.6666666666666666 |
| LRRNT_2 | 0.6666666666666666 |
| LRR_3 | 0.0 |
| LRR_4 | 0.0 |
| LRR_5 | 0.0 |
| LRR_6 | 0.6666666666666666 |
| LRR_8 | 1.0 |
| LRR_9 | 0.0 |
| Signal peptide | 1.0 |
| MALECTIN_like | 0.0 |
### Chart: Subclade XI-3
| Category | |
|---|---|
| LRR_1 | 1.0 |
| LRRNT_2 | 0.5 |
| LRR_3 | 0.0 |
| LRR_4 | 0.25 |
| LRR_5 | 0.5 |
| LRR_6 | 0.0 |
| LRR_8 | 1.0 |
| LRR_9 | 0.0 |
| Signal peptide | 0.5 |
| MALECTIN_like | 0.0 |
### Chart: Subclade XI-4
| Category | |
|---|---|
| LRR_1 | 1.0 |
| LRRNT_2 | 0.3 |
| LRR_3 | 0.0 |
| LRR_4 | 0.2 |
| LRR_5 | 0.0 |
| LRR_6 | 0.0 |
| LRR_8 | 0.8 |
| LRR_9 | 0.0 |
| Signal peptide | 1.0 |
| MALECTIN_like | 0.2 |
### Chart: Subclade XII-1
| Category | |
|---|---|
| LRR_1 | 1.0 |
| LRRNT_2 | 0.6883116883116883 |
| LRR_3 | 0.0 |
| LRR_4 | 0.3116883116883117 |
| LRR_5 | 0.0 |
| LRR_6 | 0.19480519480519481 |
| LRR_8 | 0.8831168831168831 |
| LRR_9 | 0.06493506493506493 |
| Signal peptide | 0.5064935064935064 |
| MALECTIN_like | 0.0 |
### Chart: Subclade XII-2
| Category | |
|---|---|
| LRR_1 | 0.9803921568627451 |
| LRRNT_2 | 0.19607843137254902 |
| LRR_3 | 0.0 |
| LRR_4 | 0.13725490196078433 |
| LRR_5 | 0.0 |
| LRR_6 | 0.1568627450980392 |
| LRR_8 | 0.803921568627451 |
| LRR_9 | 0.0196078431372549 |
| Signal peptide | 0.5686274509803921 |
| MALECTIN_like | 0.0 |
### Chart: Subclade XII
| Category | |
|---|---|
| LRR_1 | 1.0 |
| LRRNT_2 | 0.15384615384615385 |
| LRR_3 | 0.0 |
| LRR_4 | 0.38461538461538464 |
| LRR_5 | 0.0 |
| LRR_6 | 0.15384615384615385 |
| LRR_8 | 0.5384615384615384 |
| LRR_9 | 0.0 |
| Signal peptide | 0.8461538461538461 |
| MALECTIN_like | 0.0 |
### Chart: Outgroup
| Category | |
|---|---|
| LRR_1 | 1.0 |
| LRRNT_2 | 1.0 |
| LRR_3 | 0.0 |
| LRR_4 | 0.0 |
| LRR_5 | 0.0 |
| LRR_6 | 0.0 |
| LRR_8 | 1.0 |
| LRR_9 | 0.0 |
| Signal peptide | 1.0 |
| MALECTIN_like | 0.0 |

## Slide 26
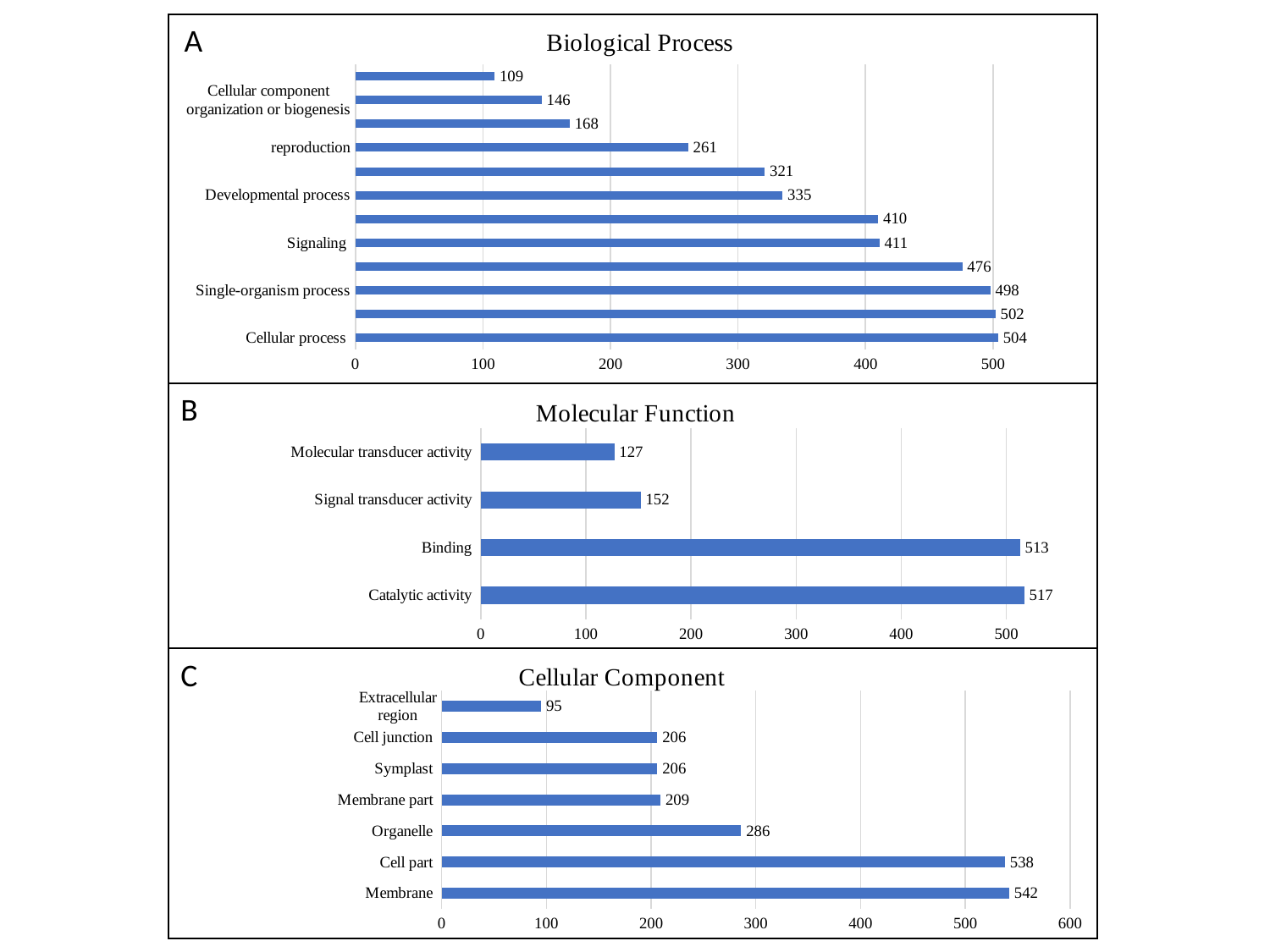

### Chart: Biological Process
| Category | |
|---|---|
| Cellular process | 504.0 |
| Response to stimulus | 502.0 |
| Single-organism process | 498.0 |
| Biological regulation | 476.0 |
| Signaling | 411.0 |
| Metabolic process | 410.0 |
| Developmental process | 335.0 |
| Multicellular organismal process | 321.0 |
| reproduction | 261.0 |
| Multi-organism process | 168.0 |
| Cellular component organization or biogenesis | 146.0 |
| Localization | 109.0 |
### Chart: Molecular Function
| Category | |
|---|---|
| Catalytic activity | 517.0 |
| Binding | 513.0 |
| Signal transducer activity | 152.0 |
| Molecular transducer activity | 127.0 |
### Chart: Cellular Component
| Category | |
|---|---|
| Membrane | 542.0 |
| Cell part | 538.0 |
| Organelle | 286.0 |
| Membrane part | 209.0 |
| Symplast | 206.0 |
| Cell junction | 206.0 |
| Extracellular region | 95.0 |
A
B
C

## Slide 27
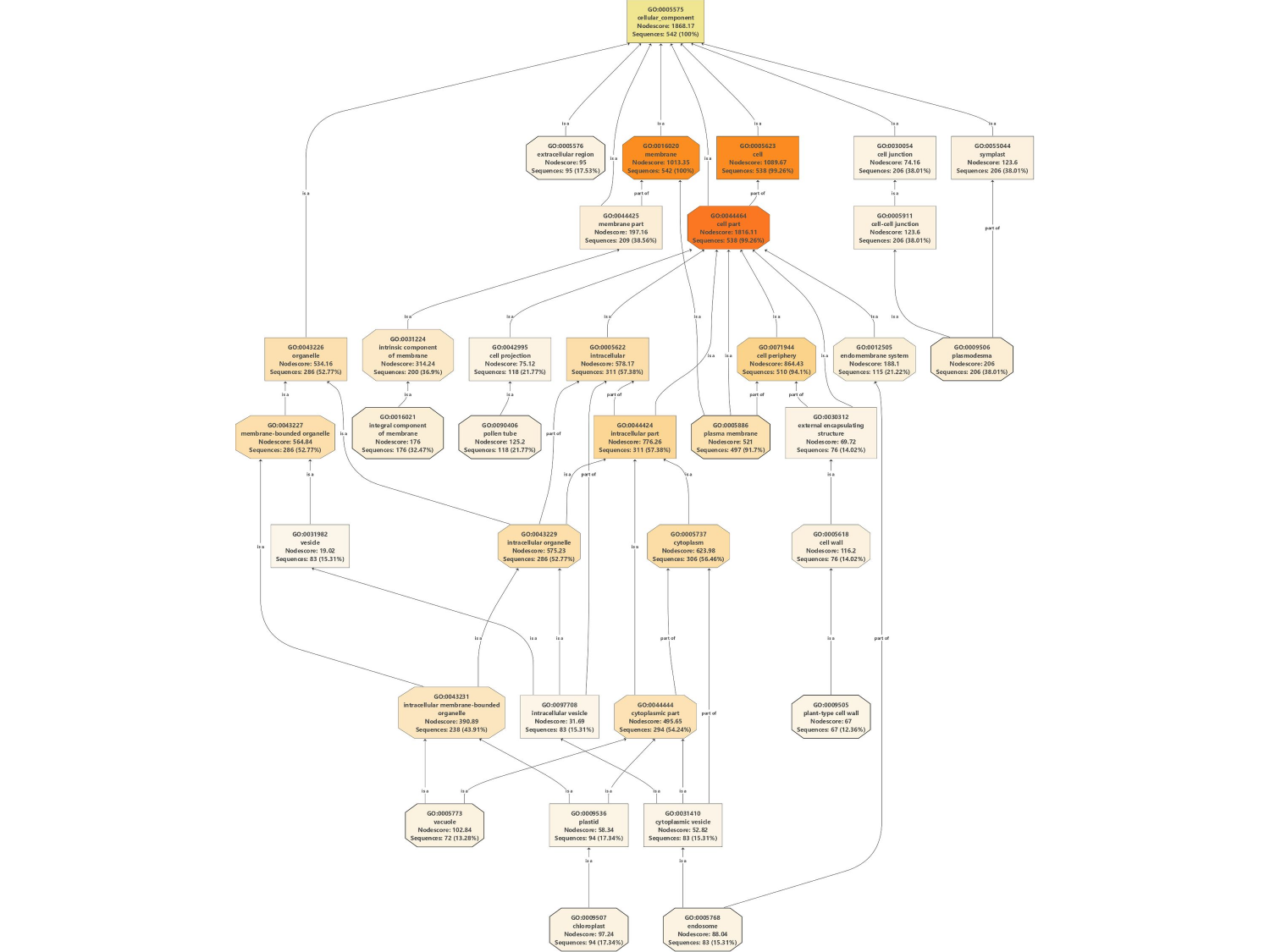

## Slide 28
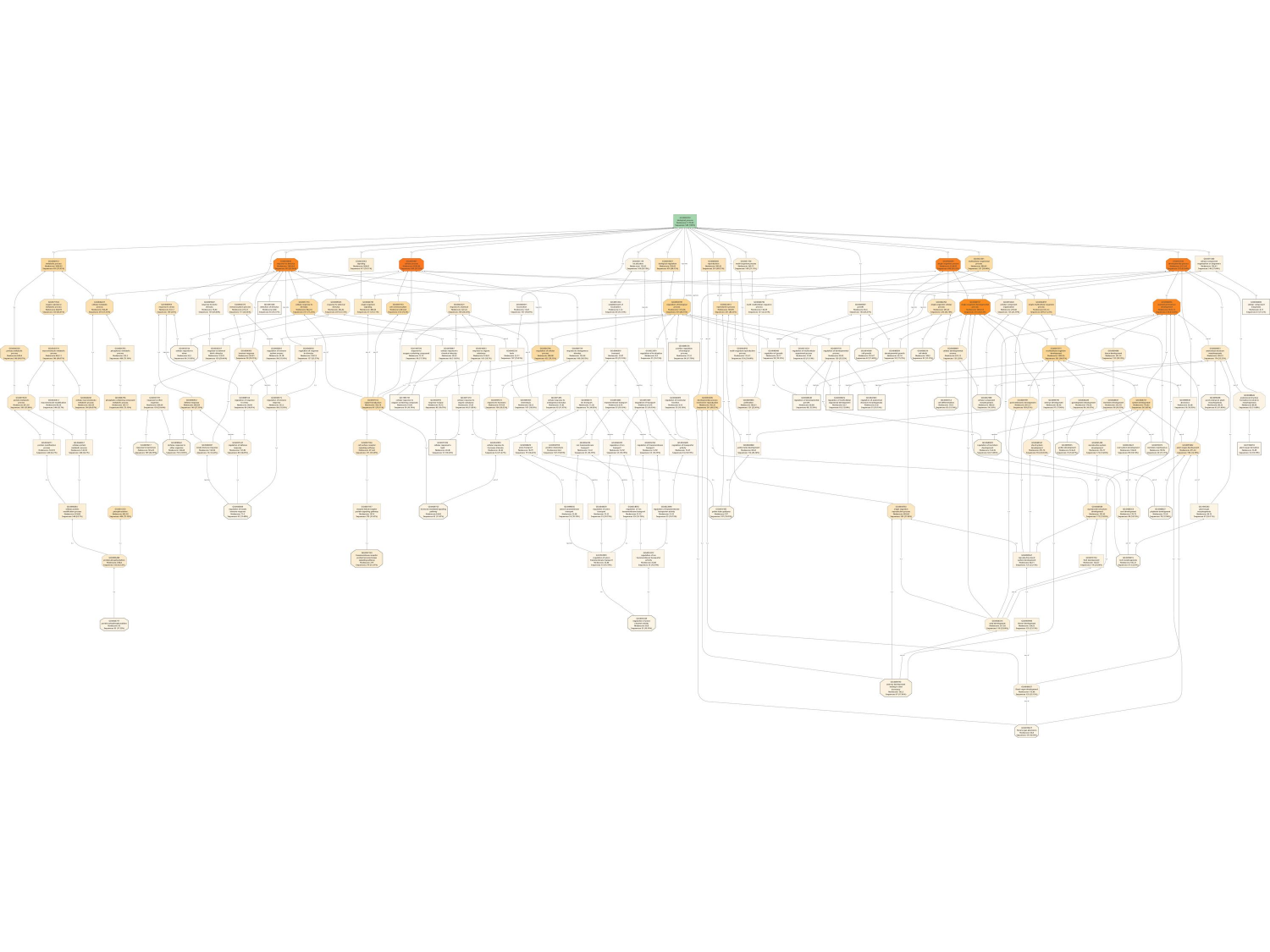

## Slide 29
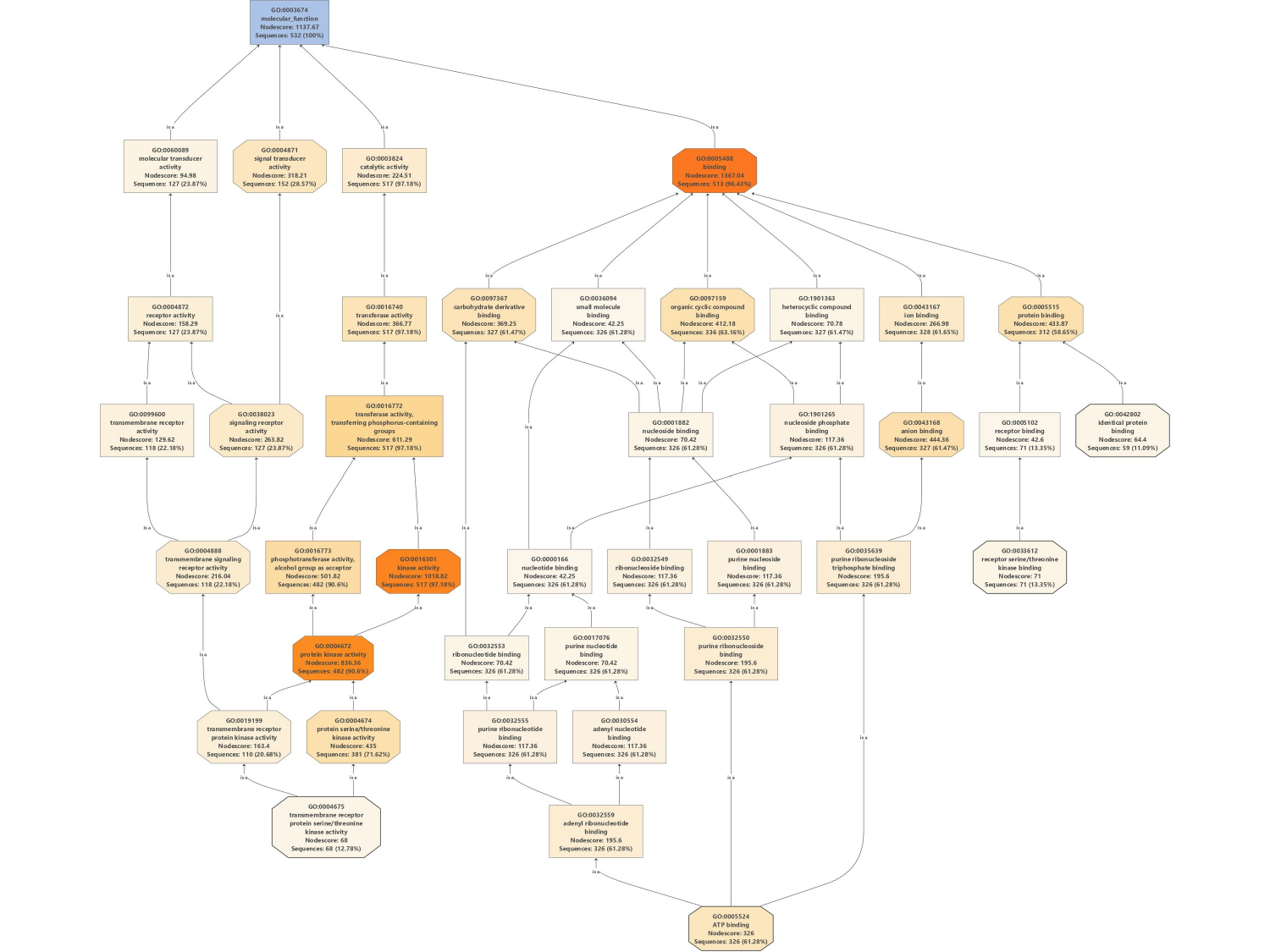

## Slide 30
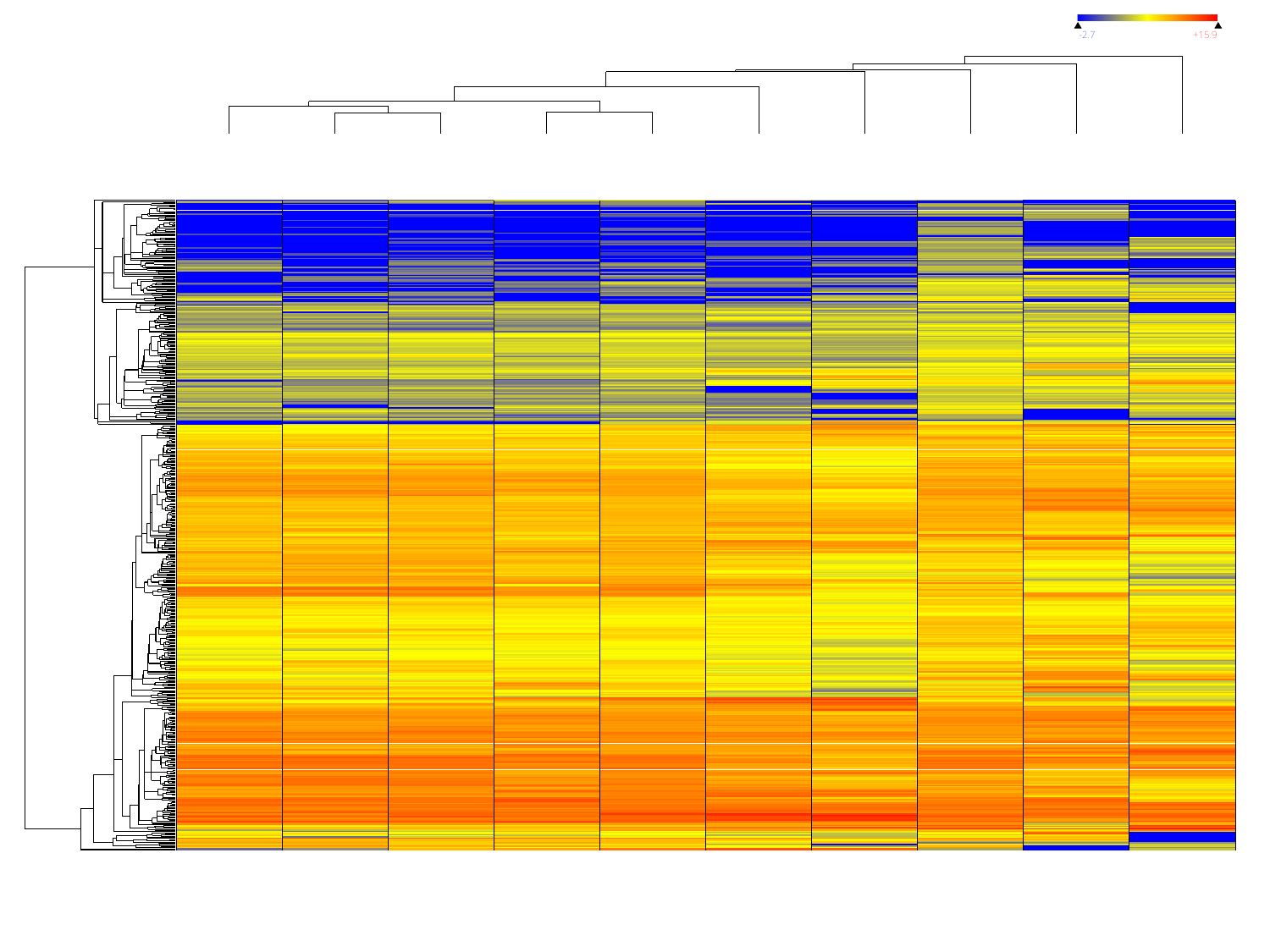

Supplement: Supplementary file 3 — Figure S1. GhLRR-RLKs Chromosomal distribution. Figure S2. Exon-intron analysis of GhLRR-RLKs. Figure S3. Protein size distribution analysis of GhLRR-RLK. Figure S4. InterProScan domains distribution of GhLRR-RLKs. Figure S5. Protein structure and domain composition of GhLRR-RLKs. Figure S6. Extracellular motif composition. Figure S7. Blas2GO annotation statistics. Figure S8. Cellular component analysis of GhLRR-RLKs. Figure S9. Biological processes analysis of GhLRR-RLKs. Figure S10. Molecular function analysis of GhLRR-RLKs. Figure S11. Expression analysis of GhLRR-RLKs. (PPTX 7480 kb) [file 12870_2018_1395_MOESM3_ESM.pptx]
